# DRIMSeq: a Dirichlet-multinomial framework for multivariate count outcomes in genomics

**DOI:** 10.12688/f1000research.8900.2

**Published:** 2016-12-06

**Authors:** Malgorzata Nowicka, Mark D. Robinson

**Affiliations:** 1 Institute for Molecular Life Sciences, University of Zurich, Zurich, 8057, Switzerland; 2 SIB Swiss Institute of Bioinformatics, University of Zurich, Zurich, 8057, Switzerland

**Keywords:** DRIMSeq, genomics, single nucleotide polymorphism, RNA-seq, splicing, statistical framework

## Abstract

There are many instances in genomics data analyses where measurements are made on a multivariate response. For example, alternative splicing can lead to multiple expressed isoforms from the same primary transcript. There are situations where differences (e.g. between normal and disease state) in the relative ratio of expressed isoforms may have significant phenotypic consequences or lead to prognostic capabilities. Similarly, knowledge of single nucleotide polymorphisms (SNPs) that affect splicing, so-called splicing quantitative trait loci (sQTL) will help to characterize the effects of genetic variation on gene expression. RNA sequencing (RNA-seq) has provided an attractive toolbox to carefully unravel alternative splicing outcomes and recently, fast and accurate methods for transcript quantification have become available. We propose a statistical framework based on the Dirichlet-multinomial distribution that can discover changes in isoform usage between conditions and SNPs that affect relative expression of transcripts using these quantifications. The Dirichlet-multinomial model naturally accounts for the differential gene expression without losing information about overall gene abundance and by joint modeling of isoform expression, it has the capability to account for their correlated nature. The main challenge in this approach is to get robust estimates of model parameters with limited numbers of replicates. We approach this by sharing information and show that our method improves on existing approaches in terms of standard statistical performance metrics. The framework is applicable to other multivariate scenarios, such as Poly-A-seq or where beta-binomial models have been applied (e.g., differential DNA methylation). Our method is available as a Bioconductor R package called DRIMSeq.

## Introduction

With the development of digital high-throughput sequencing technologies, the analysis of count data in genomics has become an important theme motivating the investigation of new, more powerful and robust approaches that handle complex overdispersion patterns while accommodating the typical small numbers of experimental units.

The basic distribution for modeling univariate count responses is the Poisson distribution, which also approximates the binomial distribution. One important limitation of the Poisson distribution is that the mean is equal to the variance, which is not sufficient for modeling, for example, gene expression from RNA sequencing (RNA-seq) data where the variance is higher than the mean due to technical sources and biological variability ^1– 5^. A natural extension of the Poisson distribution that accounts for overdispersion is the negative-binomial distribution, which has been extensively studied in the small-sample situation and has become an essential tool in genomics applications ^1– 3^.

Analogously, the fundamental distribution for modeling multivariate count data is the multinomial distribution, which models proportions across multiple features. To account for overdispersion, the multinomial can be extended to the Dirichlet-multinomial (DM) distribution ^6^. Because of its flexibility, the DM distribution has found applications in forensic genetics ^7^, microbiome data analysis ^8^, the analysis of single-cell data ^9^ and for identifying nucleosome positions ^10^. Another extension of the multinomial is the Dirichlet negative multinomial distribution ^11^, which allows modeling of correlated count data and was applied in the analysis of clinical trial recruitment ^12^. Notably, the beta-binomial distribution, such as those used in differential methylation from bisulphite sequencing data ^13– 15^, represent a special case of the DM.

Genes may express diverse transcript isoforms (mRNA variants) as a consequence of alternative splicing or due to the differences in transcription start sites and polyadenylation sites ^16^. Hence, gene expression can be viewed as a multivariate expression of transcripts or exons and such a representation allows the study of not only the overall gene expression, but also the expressed variant composition. Differences in the relative expression of isoforms can have significant phenotypic consequences and aberrations are associated with disease ^17, 18^. Thus, biologists are interested in using RNA-seq data to discover differences in transcript usage between conditions or to study the specific molecular mechanisms that mediate these changes, for example, alternative splice site usage. In general terms, we collect all these together under the term “differential splicing” (DS) ^19^.

Alternative splicing is a process regulated by complex protein-RNA interactions that can be altered by genetic variation. Knowledge of single nucleotide polymorphisms (SNPs) that affect splicing, known as splicing quantitative trait loci (sQTL), can help to characterize this layer of regulation.

In this article, we propose the DM distribution to model relative usage of isoforms. The DM model treats transcript expression as a multivariate response and allows for flexible small-sample estimation of overdispersion. We address the challenge of obtaining robust estimates of the model parameters, especially dispersion, when only a small number of replicates is available by applying an empirical Bayes approach to share information, similar to those proven successful in negative-binomial frameworks ^1, 20^. In particular, weighted likelihood is used to moderate the gene-wise dispersion toward a common or trended value.

The Dirichlet-multinomial framework, implemented as a *Bioconductor* R package called *DRIMSeq*, is applicable to both differential transcript usage (DTU) analysis between conditions and transcript usage quantitative trait loci (tuQTL) analysis. It has been evaluated and compared to the current best methods in extensive simulations and in real RNA-seq data analysis using transcript and exon counts, highlighting that *DRIMSeq* performs best with transcript counts. Furthermore, the framework can be applied to other types of emerging multivariate genomic data, such as PolyA-seq where the collection of polyadenylated sites for a given gene are measured ^21^ and to settings where the beta-binomial is already applied (e.g., differential methylation, allele-specific differential gene expression).

## Approaches to DS and sQTL analyses

RNA-seq has provided an attractive toolbox to unravel alternative splicing outcomes. There are various methods designed explicitly to detect DS based on samples from different experimental conditions ^19, 22, 23^. Independently, a set of methods was developed for detecting genetic variation associated with changes in splicing (sQTLs). While sQTL detection represents a different application, it is essentially DS between groups defined by genotypes. In the following overview, we do not distinguish between applications but rather between the general concepts used to detect differences in splicing.

**Figure 1.**
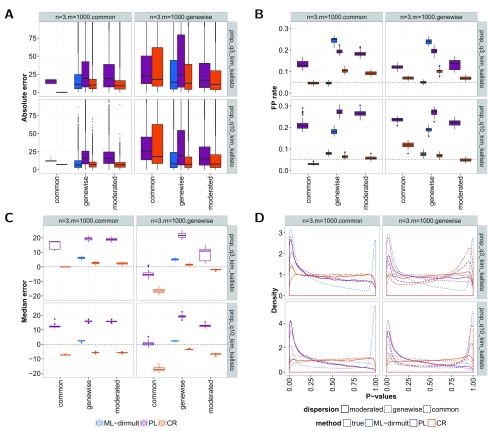
Results of two-group (3 versus 3 samples) DS analyses on data simulated from the DM null model. In the first scenario, all genes have the same (common) dispersion, and in the second one, each gene has a different (genewise) dispersion. All genes have expression equal to 1000 and 3 or 10 features with the same proportions estimated from *kallisto* counts from Kim *et al*. data set. For each of the scenarios, common, genewise, with and without moderation to common dispersion is estimated with maximum likelihood using the *dirmult* R package, the raw profile likelihood and the Cox-Reid APL. **A**: Absolute error of concentration γ _+_ estimates. **B**: False positive (FP) rate for the p-value threshold of 0.05 of the null two-group comparisons based on the likelihood ratio statistics. Dashed line indicates the 0.05 level. **C**: Median raw error of γ _+_ estimates. **D**: Distributions of p-values of the null two-group comparisons based on the likelihood ratio statistics. Additionally, results when true concentration estimates are used are indicated with the gray color.

DS can be studied in three main ways: as differential transcript usage (DTU) or, in a more local context, as differential exon or exon junction usage (DEU) or as specific splicing events (e.g., exon skipping), and all have their advantages and disadvantages. A survey of the main methods can be found in Table S1 ( Supplementary File). From the quantification perspective, exon-level abundance estimation is straightforward since it is based on counting read-region overlaps (e.g., *featureCounts* ^24^). Exons from different isoforms may have different boundaries, thus the authors of *DEXSeq* ^25^ quantify with *HTSeq* ^26^ non-overlapping windows defined by projecting all exons to the linear genome. However, this strategy does not utilize the full information from junction reads. Such reads are counted multiple times (in all exons that they overlap with), artificially increasing the total number of counts per gene and ignoring that junction reads support the isoforms that explicitly contain the combinations of exons spanned by these reads. This issue is captured in *Altrans* ^27^, which quantifies exon-links (exon junctions) or in *MISO* ^28^, *rMATS* ^29^, *SUPPA* ^30^ and *SGSeq* ^31^, all of which calculate splicing event inclusion levels expressed as percentage spliced in (PSI). Such events capture not only cassette exons but also alternative 3’ and 5’ splice sites, mutually exclusive exons or intron retention. *GLiMMPS* ^32^ and Jia *et al.* ^33^, with quantification from *PennSeq* ^34^, use event inclusion levels for detecting SNPs that are associated with differential splicing. However, there are (hypothetical) instances where changes in splicing pattern may not be captured by exon-level quantifications (Figure 1A in the paper by Monlog *et al.* ^35^). Furthermore, detection of more complex transcript variations remains a challenge for exon junction or PSI methods (see Figure S5 in the paper by Ongen *et al.* ^27^). Soneson *et al.* ^23^ considered counting which accommodates various types of local splicing events, such as exon paths traced out by paired reads, junction counts or events that correspond to combinations of isoforms; in general, the default exon-based counting resulted in strongest performance for DS gene detection.

The above methods allow for detection of differential usage of local splicing features, which can serve as an indicator of differential transcript usage but often without knowing specifically which isoforms are differentially regulated. This can be a disadvantage in cases where knowing the isoform ratio changes is important, since isoforms are the ultimate determinants of proteins. Moreover, exons are not independent transcriptional units but building blocks of transcripts. Thus, the main alternative is to make a calculation of DS using isoform-level quantitations. A vast number of methods is available for gene isoform quantification, such as *MISO* ^28^, *BitSeq* ^36^, *casper* ^37^, *Cufflinks* ^38^, *RSEM* ^39^, *FlipFlop* ^40^ and more recent, extremely fast pseudoalignment-based methods, such as *Sailfish* ^41^, *kallisto* ^42^ and *Salmon* ^43^. Additionally, *Cufflinks*, *casper* and *FlipFlop* allow for *de novo* transcriptome assembly. Recently, performance of various methods was extensively studied ^44, 45^, including a webtool ^45^ to allow further comparisons. Regardless of this progress, it remains a complex undertaking to quantify isoform expression from short cDNA fragments since there is a high degree of overlap between transcripts in complex genes; this is a limitation of the technology, not the algorithms. In the case of incomplete transcript annotation, local approaches may be more robust and can detect differential changes due to transcripts that are not in the catalog ^23, 27^. Nevertheless, DS at the resolution of isoforms is the ultimate goal within the *DRIMSeq* framework, and with the emergence of longer reads (fragments), transcript quantifications will become more accurate and methods for multivariate transcript abundances will be needed.

Whether the differential analysis is done at the transcript or local level, modeling and testing independently each transcript ^46, 47^ or exon ratio ^48^ ignores the correlated structure of these quantities (e.g., proportions must sum to 1). Similarly, separate modeling and testing of exon junctions ( *Altrans* ^27^) or splicing events ( *rMATS* ^29^, *GLiMMPS* ^32^, Jia *et al.* ^33^, Montgomery *et al.* ^49^) of a gene leads to non-independent statistical tests, although the full effect of this on calibration (e.g., controlling the rate of false discoveries) is not known. Nevertheless, with the larger number of tests, the multiple testing correction becomes more extreme. In sQTL analyses, this burden is even larger since there are many SNPs tested for each gene. There, the issue of multiple comparisons is usually accounted for by applying a permutation scheme in combination with the false discovery rate (FDR) estimation ^27, 32, 35, 46, 48– 50^.

*DEXSeq* and *voom-diffSplice* ^4, 5^ undertake another approach, where the modeling is done per gene. *DEXSeq* fits a generalized linear model (GLM), assuming that (exonic) read counts follow the negative-binomial distribution. A bin is deemed differentially used when its corresponding group-bin interaction is significantly different. The exact details of *voom-diffSplice* are not published. Nevertheless, exons are again treated as independent in the gene-level model.

In contrast, *MISO* ^28^, *Cuffdiff* ^38, 51^ and *sQTLseekeR* ^35^ model alternative splicing as a multivariate response. *MISO* is designed for DS analyses only between two samples and does not handle replicates. Variability among replicates is captured within *Cuffdiff* via the Jensen-Shannon divergence metric on probability distributions of isoform proportions as a measure of changes in isoform relative abundances between samples. *sQTLseekeR* tests for the association between genotype and transcript composition, using an approach similar to a multivariate analysis of variance (MANOVA) without assuming any probabilistic distribution and Hellinger distance as a dissimilarity measure between transcript ratios. Very recently, *LeafCutter* ^52^ gives intron usage quantifications that can be used for both DS analyses (also using the DM model) and sQTL analyses via a correlation-based approach with *FastQTL* ^50^.

*sQTLseekeR*, *Altrans*, *LeafCutter* and other earlier methods for the sQTL analysis ^35, 46– 48^ employ feature ratios to account for the overall gene expression. A potential drawback of this approach is that feature ratios do not take into account whether they are based on high or low expression, while the latter have more uncertainty in them. *DRIMSeq* naturally builds this in *via* the multinomial model.

## Dirichlet-multinomial model for relative transcript usage

In the application of the DM model to DS, we refer to *features* of a gene. These features can be transcripts, exons, exonic bins or other multivariate measurable units, which for DS, contain information about isoform usage and can be quantified with (estimated) counts.

Assume that a gene has *q* features with relative expression defined by a vector of proportions ***π*** = ( *π*_1_,…, *π _q_*), and the feature counts ***Y*** = ( *Y*_1_, …, *Y _q_*) are random variables. Let ***y*** = ( *y*_1_, …, *y _q_*) be the observed counts and $m=\sum_{j=1}^{q}\;y_{j}.$ Here, *m* is treated as an ancillary statistic since it depends on the sequencing depth and gene expression, but not on the model parameters. The simplest way to model feature counts is with the multinomial distribution with probability function defined as:

$$f_{M}\left(\text{y};\pi \right)=\left(\begin{array}{l} m\\ y \end{array}\right)\prod_{j=1}^{q}\pi_{j}^{y_{j}},\;(1)$$

where the mean and the covariance matrix of ***Y*** are ( ***Y***) = *m* ***π*** and ( ***Y***) = diag( ***π***) – ***ππ**^T^*, respectively.

To account for overdispersion due to true biological variation between experimental units as well as technical variation, such as library preparation and errors in transcript quantification, we assume the feature proportions, **Π**, follow the (conjugate) Dirichlet distribution, with density function:

$$f_{D}\left(\pi ;\gamma \right)=\frac{\Gamma \left(\gamma_{+}\right)}{\prod_{j=1}^{q}\Gamma (\gamma_{j})}\prod_{j=1}^{q}\pi_{j}^{\gamma_{j}–1},\;(2)$$

where *γ _j_*, *j* = 1, …, *q* are the Dirichlet parameters and $\gamma_{+}=\sum_{j=1}^{q}\gamma_{j}.$ The mean and covariance matrix of random proportions **Π** are ( **Π**) = ***γ***/ *γ*_+_ = ***π*** and $(\prod )=\{\gamma_{+}\text{diag}(\gamma )-\gamma \gamma^{T}\}/\{\gamma_{+}^{2}(\gamma_{+}+1)\},$ respectively. We can see that proportions **Π** are proportional to ***γ*** and their variance is inversely proportional to *γ*_+_, which is called the concentration or precision parameter. As *γ*_+_ gets larger, the proportions are more concentrated around their means.

We can derive the marginal distribution of ***Y*** by multiplying densities ( 1) and ( 2) and integrating over ***π***. Then, feature counts ***Y*** follow the DM distribution ^6^ with probability function defined as:

$$\begin{array}{l} f_{DM}\left(y;\gamma \right)=\int_{\pi }f_{M}\left(y;\pi \right)f_{D}\left(\pi ;\gamma \right)d\pi \\ \;=\left(\begin{array}{l} m\\ y \end{array}\right)\frac{\Gamma \left(\gamma_{+}\right)}{\Gamma \left(m+\gamma_{+}\right)}\prod_{j=1}^{q}\frac{\Gamma \left(y_{j}+\gamma_{j}\right)}{\Gamma \left(\gamma_{j}\right)}. \end{array}\;(3)$$

The mean of ***Y*** is unchanged at ( ***Y***) = {( ***Y***| **Π**)} = ( *m* **Π**) = *m **γ***/ *γ*_+_ = *m **π***, while the covariance matrix of ***Y*** is given by ( ***Y***) = *cm*{diag( ***π***) − ***ππ*** ^*T*^}, where *c* = ( *m*+ *γ*_+_)/(1+ *γ*_+_) is an additional factor when representing the Dirichlet-multinomial covariance to the ordinary multinomial covariance. *c* depends on concentration parameter *γ*_+_ which controls the degree of overdispersion and is inversely proportional to variance of ***Y***.

We can represent the DM distribution using an alternative parameterization: ***π*** = ***γ***/ *γ*_+_ and *θ* = 1/(1 + *γ*_+_); then, the covariance of ***Y*** can be represented as ( ***Y***) = *n*{diag( ***π***) − ***ππ*** ^*T*^} {1 + *θ*( *n* − 1)}, where *θ* can be interpreted as a dispersion parameter. When *θ* grows ( *γ*_+_ gets smaller), the variance becomes larger. From the knowledge of the gamma function, *x*Γ( *x*) = Γ( *x* + 1), we can write $\frac{\Gamma \left(\alpha +x\right)}{\Gamma \left(\alpha \right)}=\prod_{r=1}^{x}\left\{\alpha +\left(r–1\right)\right\}.$ Hence, the DM density function becomes:

$$f_{DM}\left(y;\pi ,\theta \right)=\left(\begin{array}{l} m\\ y \end{array}\right)\frac{\prod_{j=1}^{q}\prod_{r=1}^{y_{j}}\left\{\pi_{j}\left(1-\theta \right)+\left(r-1\right)\theta \right\}}{\prod_{r=1}^{m}\left\{1-\theta +\left(r-1\right)\theta \right\}},\;(4)$$

such that for *θ* = 0, DM reduces to multinomial.

## Detecting DTU and tuQTLs with the Dirichlet-multinomial model

Within *DRIMSeq*, the DM method can be used to detect the differential usage of gene features between two or more conditions. For simplicity, suppose that features of a gene are transcripts and the comparison is done between two groups. The aim is to determine whether transcript ratios of a gene are different in these two conditions. Formally, we want to test the hypothesis *H*_0_ : ***π***_1_ = ***π***_2_ against the alternative *H*_1_ : ***π***_1_ ≠ ***π***_2_. For the convenience of parameter estimation, we decide to use the DM parameterization with precision parameter *γ*_+_, which can take any non-negative value, instead of dispersion parameter *θ*, which is bounded to values between 0 and 1. Because our goal is to compare the proportions from two groups, *γ*_+_ is a nuisance parameter that gets estimated in the first step (see the following Section). Let *l*( ***π*_1_**, ***π*_2_**, *γ*_+_) be the joint log-likelihood function. Assuming $\gamma_{+}={\hat{\gamma }}_{+},$ the maximum likelihood (ML) estimates of ***π*_1_**, ***π*_2_** are the solution of $\frac{dl}{d\left(\pi_{1},\pi_{2}\right)}\left(\pi_{1},\pi_{2},\gamma_{+}={\hat{\gamma }}_{+}\right)=0.$ Under the hypothesis *H*_1_ : ***π*_1_** = ***π*_2_** = ***π***, the ML estimate of ***π*** is the solution of $\frac{dl}{d\pi }\left(\pi_{1}=\pi ,\pi_{2}=\pi ,\gamma_{+}={\hat{\gamma }}_{+}\right)=0.$ We test the null hypothesis using a likelihood ratio statistic of the form

$$\begin{array}{l} D=2l\left(\pi_{1}={\hat{\pi }}_{1},\pi_{2}={\hat{\pi }}_{2},\gamma_{+}={\hat{\gamma }}_{+}\right)\\ \;–2l\left(\pi_{1}=\hat{\pi },\pi_{2}=\hat{\pi },\gamma_{+}={\hat{\gamma }}_{+}\right), \end{array}\;(5)$$

which asymptotically follows the chi-squared distribution $\chi_{q–1}^{2}$ with q − 1 degrees of freedom. In comparisons across *c* groups, the number of degrees of freedom is ( *c* − 1) × ( *q* − 1). After all genes are tested, p-values can be adjusted for multiple comparisons with the Benjamini-Hochberg method.

In a DTU analysis, groups are defined by the design of an experiment and are the same for each gene. In tuQTL analyses, the aim is to find nearby (bi-allelic) SNPs associated with transcript usage of a gene. Model fitting and testing is performed for each gene-SNP pair, and grouping of samples is defined by the genotype, typically translated into the number of minor alleles (0, 1 or 2). Thus, tuQTL analyses are similar to DTU analyses with the difference that multiple models are fitted and tested for each gene. Additional challenges to be handled in tuQTL analyses include a large number of tests per gene with highly variable allele frequencies (models) and linkage disequilibrium, which can be accounted for in the multiple testing corrections. As in other sQTL studies ^35, 49, 50^, we apply a permutation approach to empirically assess the null distribution of associations and use it for the adjustment of nominal p-values (see Supplementary Note 2 in Supplementary File). For computational efficiency, SNPs within a given gene that exhibit the same genotypes are grouped into blocks. In this way, blocks define unique models to be fit, reducing computation and the degree of multiple testing correction.

## Dispersion estimation with adjusted profile likelihood and moderation

Accurate parameter estimation is a challenge when only a small number of replicates is available. Following the *edgeR* strategy ^1, 2, 53^, we propose multiple approaches for dispersion estimation, all based on the maximization and adjustment of the profile likelihood, since standard maximum likelihood (ML) is known to produce biased estimates as it tends to underestimate variance parameters by not allowing for the fact that other unknown parameters are estimated from the same data ^54, 55^.

In the DM model parameterization of our choice, we are interested in estimating the precision (concentration) parameter, *γ*_+_ (inverse proportional to dispersion *θ*). Hence, at this stage, proportions ***π*_1_** and ***π*_2_** can be considered nuisance parameters and the profile log-likelihood ( *PL*) for *γ*_+_ can be constructed by maximizing the log-likelihood function with respect to proportions ***π*_1_** and ***π*_2_** for fixed *γ*_+_:

$$PL\left(\gamma_{+};{\hat{\pi }}_{1},{\hat{\pi }}_{2},y\right)=\underset{\pi_{1},\pi_{2}}{\max}l\left(\pi_{1},\pi_{2},\gamma_{+},y\right).\;(6)$$

The profile likelihood is then treated as an ordinary likelihood function for estimation and inference about parameters of interest. Unfortunately, with large numbers of nuisance parameters, this approach can produce inefficient or even inconsistent estimates ^54, 55^. To correct for that, one can apply an adjustment proposed by Cox and Reid ^56^ and obtain an adjusted profile likelihood ( *APL*):

$$APL\left(\gamma_{+};{\hat{\pi }}_{1},{\hat{\pi }}_{2},y\right)=PL\left(\gamma_{+};{\hat{\pi }}_{1},{\hat{\pi }}_{2},y\right)–\frac{1}{2}\log\left(\det mI\right),\;(7)$$

where *det* denotes determinant and *I* is the observed information matrix for **π _1_** and **π _2_**. The interpretation of the correction term in *APL* is that it penalizes values of *γ*_+_ for which the information about **π _1_** and **π _2_** is relatively large. When data consists of many samples, one can use gene-wise dispersion estimates, i.e., the dispersion is estimated for each gene *g* = 1,…, *G* separately:

argmax{APLg(γ+g)}=argmax{AP L(γ+g;π^1g,π^2g,yg)}.(8)

These estimates become more unstable as the sample size decreases. At the other extreme, one can assume a common dispersion for all genes and use all genes to estimate it:

argmax{1G∑g=1GAP Lg(γ+g)}.(9)

Common dispersion estimates are more stable but the assumption of a single dispersion for all genes is rather strong, given that some genes are under tighter regulation than others ^57, 58^. Thus, moderated dispersion is a trade-off between gene-wise and common dispersion and estimates are calculated with an empirical Bayes approach, which uses a weighted combination of the common and individual likelihood:

argmax{AP Lg(γ+g)+W.1G∑g=1GAPLg(γ+g)}.(10)

If a dispersion-mean trend is present (see Figure S16, Figure S17, Figure S28 and Figure S29 in Supplementary File), as commonly observed in gene-level differential expression analyses ^1, 3^, one can apply shrinkage towards this trend instead of to the common dispersion:

$$\text{arg}\;\text{max}\left\{APL_{g}\left(\gamma_{+}^{g}\right)+W.\frac{1}{\left|C\right|}\sum_{g\in C}^{}APL_{g}\left(\gamma_{+}^{g}\right)\right\},\;(11)$$

where *C* is a set of genes that have similar gene expression as gene *g* and *W* is a weight defining the strength of moderation (see Supplementary Note 1 for further details).

## Estimation and inference: simulations from the Dirichlet-multinomial model

We first investigated the performance of the DM model and the approach for parameter estimation and inference in the case where only few replicates are available. We performed simulations that correspond to a two-group comparison with no DTU (i.e. null model) where feature counts were generated from the DM distribution with identical parameters in both groups. Simulations were repeated 50 times for 1000 genes. In these simulations, we can vary the overall expression (m), number of features (q), proportions (prop) and sample size in one condition (n). Proportions follow a uniform or decaying distribution or are estimated based on *kallisto* transcripts or *HTSeq* exon counts from Kim *et al*. and Brooks *et al*. data (more details on these datasets below). In the first case, all genes have the same (common) dispersion, and in the second one, each gene has different (genewise) dispersion. Simulations for evaluating the dispersion moderation are intended to better resemble a real dataset. For these instances (repeated 25 times for 5000 genes), genes have expression, dispersion and proportions that were estimated from the real data. See Supplementary Note 3 for the additional details.

Figure 1A and Figure S1 confirm that using the Cox-Reid adjustment (CR) improves the estimation (in terms of median absolute error and extreme error values) of the concentration parameter *γ*_+_ in comparison to raw profile likelihood (PL) estimates. Additionally, the median error of concentration estimates for Cox-Reid APL is always lower than for PL or maximum likelihood (ML) used in the *dirmult* package ^7^ ( Figure 1C, Figure S2). This translates directly into the inference performance where the CR approach leads to lower false positive (FP) rate than other approaches ( Figure 1B, Figure S3).

Accurate estimates of dispersion do not always lead to expected control of FP rate. Notably, using the true concentration parameters in genes with many features (with decaying proportions) results in higher than expected nominal FP rates ( Figure 1B, Figure S3 and Figure S6A). Meanwhile, for genes with uniform proportions, even with many features, the FP rate for true dispersion is controlled ( Figure S3 and Figure S6B). Also, the Cox-Reid adjustment tends to underestimate the concentration (overestimate dispersion) for genes with many features and decaying proportions, especially for very small sample size ( Figure 1C, Figure S2, Figure S5A, Figure S5E), which leads to accurate FP rate control not achieved even with the true dispersion ( Figure S6A).

As expected, common dispersion estimation is effective when all genes indeed have the same dispersion, though this cannot be generally assumed in most real RNA-seq datasets (see results of simulations in the following section). In contrast, pure gene-wise estimates of dispersion lead to relatively high estimation error in small sample sizes ( Figure 1A, Figure S1 and Figure S8). Thus, sharing information about concentration (dispersion) between genes by moderating the gene-wise APL is applied. This improves concentration estimation in terms of median error ( Figure 1C and Figure S8) and by shrinking extremely large values (on the boundary of the parameter space, see Figure S7) toward common or trended concentration. Therefore, moderated gene-wise estimates lead to better control of the nominal FP rate ( Figure 1B and Figure S10).

In these simulations, the overall best performance of the DM model is achieved when dispersion parameters are estimated with the Cox-Reid APL and the dispersion moderation is applied. This strategy leads to p-value distributions that in most of the cases are closer to the uniform distribution ( Figure 1D, Figure S4 and Figure S11).

## Comparison on simulations that mimic real RNA-seq data

Next, we use the simulated data from Soneson *et al*. ^23^, where RNA-seq reads were generated such that 1000 genes had isoform switches between two conditions of the two most abundant transcripts. For each condition three replicates were simulated resulting in 3 versus 3 comparisons. Altogether, we summarize results for three scenarios: i) Drosophila melanogaster with no differential gene expression; ii) Homo sapiens without differential gene expression; iii) Homo sapiens with differential gene expression.

The aim of these analyses is to compare the performance of *DRIMSeq* against *DEXSeq*, which emerged among the top performing methods for detection of DTU from RNA-seq data ^23^. For *DRIMSeq*, we consider different dispersion estimates: common, gene-wise with no moderation and with moderation-to-common and to-trended dispersion. We use the exonic bin counts provided by *HTSeq* (same input to the *DEXSeq* pipeline), and transcript counts obtained with *kallisto*. Additionally, we use *HTSeq* and *kallisto* counts that are re-estimated after the removal of lowly expressed transcripts (less than 5% in all samples) from the gene annotation (pre-filtering) as proposed by Soneson *et al*. ^23^ and *kallisto* filtered counts that exclude the lowly expressed transcripts (also less than 5% in all samples). *DRIMSeq* returns a p-value per gene. To make results comparable, we used the module within *DEXSeq* that summarizes exon-level p-values to a gene-level adjusted p-value.

**Figure 2.**
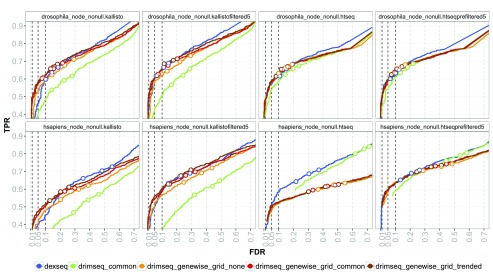
True positive rate (TPR) versus achieved false discovery rate (FDR) for three FDR thresholds (0.01, 0.05 and 0.1) obtained by *DEXSeq* and *DRIMSeq*. *DRIMSeq* was run with different dispersion estimation strategies: common dispersion and genewise dispersion with no moderation (genewise_grid_none), moderation to common dispersion (genewise_grid_common) and moderation to trended dispersion (genewise_grid_trended). Results presented for Drosophila melanogaster and Homo sapiens simulations with DTU (nonull) and no differential gene expression (node) using transcript counts from *kallisto* and exonic counts from *HTSeq*. Additionally, filtered counts (kallistofiltered5, htseqprefiltered5) are used. When the achieved FDR is smaller than the threshold, circles are filled with the corresponding color, otherwise, they are white.

As expected, common dispersion estimates lead to worse performance (lower power and higher FDR) compared to gene-wise dispersions. *DRIMSeq* achieves the best performance with moderated gene-wise dispersion estimates, while the difference in performance between moderating to common or to trended dispersion is quite small, with moderated-to-trend dispersion estimates being slightly more conservative ( Figure 2 and Figure S15).

As noted by Soneson *et al*. ^23^, detecting DTU in human is harder than in fruit fly due to the more complex transcriptome of the first one; all methods have much smaller false discovery rate (FDR). Nevertheless, none of the methods manages to control the FDR at a given threshold in either of the simulations.

Annotation pre-filtering, suggested as a solution to mitigate high FDRs ^23^, affects *DEXSeq* and *DRIMSeq* in a different way. For *DEXSeq*, it strongly reduces the FDR. For *DRIMSeq*, it increases power without a strong reduction of FDR. Moreover, the results for *kallisto* filtered and pre-filtered are almost identical ( Figure S15 and Figure S24), which means that the re-estimation step based on the reduced annotation is not necessary for *kallisto* when used with *DRIMSeq* or *DEXSeq*. Additionally, we have considered how other filtering approaches affect DTU detection.

From Figure S24, we can see that DS analysis based on transcript counts are more robust to different variations of filtering and indeed some filtering improves the inference. For exonic counts, filtering should be less stringent and the pre-filtering approach is the best performing strategy.

*DRIMSeq* performs well when coupled with transcript counts from *kallisto*. In the case when no filtering is applied to the data, it outperforms *DEXSeq*. When transcript counts are pre-filtered, both methods have very similar performance ( Figure S15). For both differential engines, the performance decreases substantially with increasing number of transcripts per gene, with *DRIMSeq* having slightly more power when genes have only a few transcripts ( Figure S17). *DRIMSeq* has poor performance for the exonic counts in the human simulation, where achieved FDRs of more than 50% are observed for an expected 5%; consequently, we recommend the use of *DRIMSeq* on transcript counts only. On the other hand, the concordance of *DRIMSeq* and *DEXSeq* top-ranked genes is quite high and similar even for exonic counts ( Figure S16).

The p-value distributions highlight a better fit of the DM model to transcript counts compared to exonic counts (it is more uniform with a sharp peak close to zero). Similarly, dispersion estimation gives better results for transcript counts ( Figure S19 and Figure S20). In particular, for exonic counts, a large number of genes have concentration parameter estimates at the boundary of the parameter space, unlike the situation for transcript counts ( Figure S19 and Figure S20).

## DS analyses on real datasets

To compare the methods further, we consider two public RNA-seq data sets. The first is the pasilla dataset ^59^ (Brooks *et al.*). The aim was to identify genes regulated by *pasilla*, the Drosophila ortholog of mammalian splicing factors *NOVA1* and *NOVA2*. In this experiment, libraries were prepared from seven biologically independent samples: four control samples and three samples in which *pasilla* was knocked down. Libraries were sequenced using a mixture of single-end and paired-end reads as well as different read lengths. The second data set is from matched human lung normal and adenocarcinoma samples from six Korean female nonsmoking patients ^60^, using paired-end reads (Kim *et al.*).

Both datasets have a more complex design than those used in the simulations; in addition to the grouping variable of interest, there are additional covariates to adjust for (e.g., library layout for the fruit fly data, patient identifier for the paired human study). In order to account for such effects, one should rather use a regression approach, which currently is not supported by *DRIMSeq*, but can be applied within *DEXSeq*’s GLM framework. To make the comparison fair, we fit multiple models. For the pasilla dataset, we compare four control samples versus three pasilla knock-down samples without taking into account the library layout (model full) as well as compare only the paired-end samples, which removes the covariate. To not diminish *DEXSeq* for its ability to fit more complex models, we run it using a model that does the four control versus three knock-down comparison with library layout as an additional covariate (model full 2). For the adenocarcinoma data, we do a two-group comparison of six normal versus six cancer samples (model full) and for *DEXSeq*, we fit an extra model that takes into account patient effects (model full 2). Additionally, we do so-called "mock" analyses where samples from the same condition are compared (model null), and the expectation is to detect no DS since it is a within-condition comparison (see Supplementary Note 5 for the exact definition of these null models).

In the full comparisons with transcript counts, *DRIMSeq* calls similar or fewer DS genes than *DEXSeq*, and a majority of them are contained within the *DEXSeq* calls ( Figure S26, Figure S27) showing high concordance between *DRIMSeq* and *DEXSeq* and slightly more conservative nature of *DRIMSeq*. Accounting for covariates in *DEXSeq* (model full 2) or performing the analysis on a subgroup without covariates (model full paired) results in more DS genes detected ( Figure S28, Figure S29 and Figure S30).

In the "mock" analyses, as expected, both methods detect considerably fewer DS genes, except in two cases. First, for the pasilla data (model null 3), where the two *versus* two control samples were from single-end library in one group and from paired-end library in the second group, leading to a comparison between batches in which both of the methods found more DS genes than in the comparison of control versus knock-down showing that the "batch" effect is very strong. Second, in the adenocarcinoma data (model null normal 1), where the two groups of individuals (each consisting of three women) happened to be very distinct ( Figure S25). Therefore, we do not include these two cases when referring to the null models.

Overall, in the full comparisons, there are more DS genes detected based on differential transcript usage than differential exon usage ( Figure S26). For *DEXSeq*, this is also the case in the null comparisons, which shows that *DEXSeq* works better with exonic counts than with transcript counts. *DRIMSeq*, on the other hand, has better performance on transcript counts, for which it calls less DS genes in the null analysis than with exon counts. In particular, the p-value distributions under the null indicate that DM fits better to transcript counts than exon counts ( Figure S14, Figure S31 and Figure S32).

**Figure 3.**
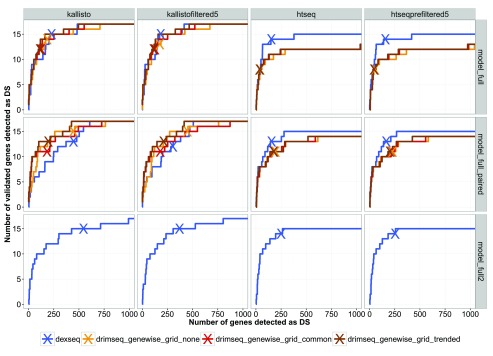
Results of DS analysis on the pasilla dataset showing how many of the 16 validated genes are called by *DRIMSeq* and *DEXSeq* using different counting strategies and different models. On each curve, "X" indicates the number of DS genes detected for the FDR of 0.05. Model full - comparison of 4 control samples versus 3 knock-down. Model full paired - comparison of 2 versus 2 paired-end samples. Model full 2 - as model full but including the information about library layout (no results for *DRIMSeq* because currently, it is not able to fit models with multiple covariates).

Method comparisons based on real data are very challenging as the truth is simply not known. In this sense the pasilla data is very precious, as the authors of this study have validated alternative usage of exons in 16 genes using RT-PCR. Of course, these validations represent an incomplete truth, and ideally, large-scale independent validation would be needed to comprehensively compare the DTU detection methods. In Figure 3, Figure S33, Figure S34 and Figure S35 again it is shown that *DRIMSeq* is slightly more conservative than *DEXSeq*. *DRIMSeq* performs poorly on exon-level but returns strong performance on transcript-level quantification (e.g., *kallisto*) and even outperforms *DEXSeq* when the sample size is very small (model full paired).

## tuQTL analyses

To demonstrate the application of *DRIMSeq* to tuQTL analysis, we use the data from the GEUVADIS project ^46^ where 465 RNA-seq samples from lymphoblastoid cell lines were sequenced, 422 of which were sequenced in the 1000 Genomes Project Phase 1. Here, we present the analysis of 91 samples corresponding to the CEU population and 89 samples from the YRI population. Expected transcript counts (obtained with Flux Capacitor) and genotype data were downloaded from the GEUVADIS project website. We choose to compare the performance of *DRIMSeq* with *sQTLseekeR*, because it is a very recent tool that performs well ^35^, can be directly applied to transcript count data and models transcript usage as a multivariate outcome.

**Figure 4.**
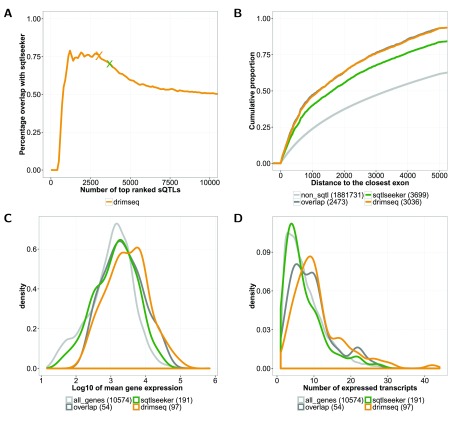
Results of the tuQTL analysis on the CEU population from the GEUVADIS data. **A**: Concordance between *sQTLseekeR* and *DRIMSeq*. "X" indicates number of tuQTLs for FDR = 0.05. Panel **B**, **C** and **D** show characteristics of tuQTLs and genes detected by *sQTLseekeR* or *DRIMSeq* for FDR = 0.05. Values in brackets indicate numbers of tuQTLs or genes in a given set. Dark gray line corresponds to tuQTLs or genes that were identified by both of the methods (overlap). **B**: Distance to the closest exon of intronic tuQTLs. The light gray line (non_sqtl) corresponds to intronic tuQTLs that were not called by any of the methods. **C**: Distribution of mean gene expression for genes that are associated with tuQTLs. **D**: Distribution of the number of expressed transcripts for genes that are associated with tuQTLs. The light gray lines (all_genes) represent corresponding features for all the analyzed genes.

For both of the methods, we investigate only the bi-allelic SNPs with a minor allele present in at least five samples (minor allele frequency approximately equal to 5%) and at least two alleles present in a population. Given a gene, we keep the SNPs that are located within 5 Kb upstream or downstream of the gene. We use the same pre-filtered counts in *DRIMSeq* and *sQTLseekeR* to have the same baseline for the comparison of the statistical engines offered by these packages. We keep the protein coding genes that have at least 10 counts in 70 or more samples and at least two transcripts left after the transcript filtering, which keeps the one that has at least 10 counts and proportion of at least 5% in 5 or more samples. The numbers of tested and associated genes and tuQTLs are indicated in Figure 4, Figure S38 and Figure S39.

In Figure 4A and Figure S40 we can see that the concordance between *DRIMSeq* and *sQTLseekeR* is quite high and reaches 75%. Nevertheless, there is considerable difference between the number and type of genes that are uniquely identified by each method. *sQTLseekeR* finds more genes with alternative splicing associated to genetic variation ( Figure S38 and Figure S39), but these genes have fewer transcripts expressed and lower overall expression in comparison to genes detected by *DRIMSeq* ( Figure 4C, Figure 4D, Figure S40C and Figure S40D). To further investigate characteristics of detected tuQTLs, we measured enrichment of splicing-related features as used in a previous comparison ^35^. This includes the location of tuQTLs within exons, within splice sites, in the surrounding of GWAS SNPs and distance to the closest exon. tuQTLs detected by *DRIMSeq* show higher enrichment for all splicing related features ( Table 1 and Figure 4B), than *sQTLseekeR* tuQTLs, suggesting that by accounting for the overall gene expression, one can detect more meaningful associations.

**Table 1.** Enrichment in splicing related features for tuQTLs detected by *DRIMSeq* and *sQTLseekeR* in CEU and YRI populations for FDR = 0.05.

	% within exons		% within splice sites		% within 1Kb of a GWAS	
	CEU	YRI	CEU	YRI	CEU	YRI
DRIMSeq	26.09	35.89	19.76	21.42	12.75	15.43
sQTLseekeR	20.95	25.43	13.52	17.4	10.22	10.09
Overlap	26.85	40.58	16.17	25.36	13.42	18.14
Non tuQTLs	5.25	5.24	1.75	1.53	1.15	0.97

## Discussion

We have created a statistical framework called *DRIMSeq* based on the Dirichlet-multinomial distribution to model alternative usage of transcript isoforms from RNA-seq data. We have shown that this framework can be used for detecting differential isoform usage between experimental conditions as well as for identifying tuQTLs. In principle, the framework is suitable for differential analysis of any type of multinomial-like responses. From our simulations and real data analyses towards DS and sQTL analyses, *DRIMSeq* seems better suited to model transcript counts rather than exonic counts.

Overall, there are many tradeoffs to be made in DS analyses. For example, deriving transcript abundances from RNA-seq data is more difficult (e.g., complicated overlapping genes at medium to low expression levels) than directly counting exon inclusion levels of specific events. On the other hand, local splicing events may be not able to capture biologically interesting splice changes (e.g., switching between two different transcripts) but have ultimately more ability to detect DS in case when the transcript catalog is incomplete. Despite these tradeoffs and given the results observed here, *DRIMSeq* finds its place as a method to make downstream calculations on transcript quantifications. With emerging technologies that sequence longer DNA fragments (either truly or synthetically), we may see in the near future more direct counting of full-length transcripts, making transcript-level quantification more robust and accurate. Even with current standard RNA-seq data, ultrafast and lightweight methods make transcript counting more accessible and users will want to make comparative analyses directly from these estimates.

In principle, existing DS methods that allow multiple group comparisons could be adapted to the sQTL framework and *vice versa*; *DRIMSeq* is one of few tools that bridge these two applications. In particular, parameter estimation with *DRIMSeq* is suited for a situation where only a few replicates are available per group (common in DS analysis) as well as analyses over larger samples sizes (typical in sQTL analysis). For small sample sizes, accurate dispersion estimation is especially challenging. Thus, we incorporate estimation techniques analogous to those used in negative binomial frameworks, such as Cox-Reid APL; perhaps not surprisingly, raw profile likelihood or standard maximum likelihood approaches do not perform as well in our tests of estimation performance. In addition, as with many successful genomics modeling frameworks, sharing information across genes leads to more stable and accurate estimation and therefore better inference (e.g., better control of nominal FP rates).

In comparison to other available methods, *DRIMSeq* seems to be more conservative than both *DEXSeq* (using transcript counts) and *sQTLseekeR*, identifying fewer DTU genes and tuQTLs, respectively. On the other hand, *DEXSeq* is known to be somewhat liberal ^23^. Moreover, the sQTL associations detected by *DRIMSeq* have more enrichment in splicing-related features than *sQTLseekeR* tuQTLs, which could be due to the fact that *DRIMSeq* accounts for the higher uncertainty of lowly expressed genes by using transcript counts instead of transcript ratios.

Our developed DM framework is general enough that it can be applied to other genomic data with multivariate count outcomes. For example, PolyA-seq data quantifies the usage of multiple RNA polyadenylation sites. During polyadenylation, poly(A) tails can be added to different sites and thus more than one transcript can be produced from a single gene (alternative polyadenylation); comparisons between groups of replicates can be conducted with *DRIMSeq*. As mentioned, the DM distribution is a multivariate generalization of the beta-binomial distribution, as the binomial and beta distributions are univariate versions of the multinomial and Dirichlet distributions, respectively. Although untested here, the *DRIMSeq* framework could be applied to analyses where the beta-binomial distribution are used with the advantage of naturally accommodating small-sample datasets. Interesting beta-binomial-based analyses include differential methylation using bisulphite sequencing data, where counts of methylated and unmethylated cytosines (a bivariate outcome) at specific genomic loci are compared, or allele-specific gene expression, where the expression of two alleles (again, a bivariate outcome) are compared across experimental groups.

One particularly important future enhancement is a regression framework, which would allow direct analysis of more complex experimental designs. For example, covariates such as batch, sample pairing or other factors could be adjusted for in the model. In the tuQTL analysis, it would allow studying samples from the pooled populations, with the subpopulation as a covariate, allowing larger sample sizes and increased power to detect interesting changes. Another potential limitation is that *DRIMSeq* treats transcript estimates as fixed, even though they have different uncertainty, depending on the read coverage and complexity of the set of transcripts within a gene. Although untested here, propagation of this uncertainty could be achieved by incorporating observational weights that are inversely proportional to estimated uncertainties or, in case of fast quantification methods like *kallisto*, by making effective use of bootstrap samples. At this stage, there is no consensus on how these approaches will perform and ultimately may require considerable additional computation.

## Software availability

The Dirichlet-multinomial framework described in this paper is implemented within an R package called DRIMSeq. In addition to the user friendly workflow for the DTU and tuQTL analyses, it provides plotting functions that generate diagnostic figures such as the dispersion versus mean gene expression figures and histograms of p-values. User can also generate figures of the observed proportions and the DM estimated ratios for the genes of interest to visually investigate their individual splicing patterns.

The release version of *DRIMSeq* is available on Bioconductor http://bioconductor.org/packages/DRIMSeq, and the latest development version can be found on GitHub https://github.com/markrobinsonuzh/DRIMSeq.

## Data availability

The data referenced by this article are under copyright with the following copyright statement: Copyright: © 2016 Nowicka M and Robinson MD

Data for simulations that mimic real RNA-seq was obtained from Soneson *et al.* ^23^, where all the details on data generation and accessibility are available.

Differential splicing analyses were performed on the publicly available pasilla dataset, which was downloaded from the NCBI’s Gene Expression Omnibus (GEO) under the accession number GSE18508, and adenocarcinoma dataset under the accession number GSE37764.

Data for the tuQTL analyses was downloaded from the GEUVADIS project website.

All the details about data availability and preprocessing are described in the Supplementary Materials.

### Archived source code as at the time of publication

*DRIMSeq* analyses for this paper were done with version 0.3.3 available on Zenodo https://zenodo.org/record/53084 ^61^ and Bioconductor release 3.2. Source code used for the analyses in this paper is available on Zenodo https://zenodo.org/record/167305 ^62^.

## Supplementary material

**Supplementary File 1.** Contains supplementary figures and tables referred to in the text. It also contains descriptions of dispersion moderation and p-value adjustment in tuQTL analysis and details about the simulations and real data analyses.

## References

[1] McCarthy DJ Chen Y Smyth GK: Differential expression analysis of multifactor RNA-Seq experiments with respect to biological variation. *Nucleic Acids Res.* 2012;40(10):4288–4297. 10.1093/nar/gks042 22287627 PMC3378882

[2] Robinson MD Smyth GK: Small-sample estimation of negative binomial dispersion, with applications to SAGE data. *Biostatistics.* 2008;9(2):321–332. 10.1093/biostatistics/kxm030 17728317

[3] Anders S Huber W: Differential expression analysis for sequence count data. *Genome Biol.* 2010;11(10):R106. 10.1186/gb-2010-11-10-r106 20979621 PMC3218662

[4] Ritchie ME Phipson B Wu D: *Limma* powers differential expression analyses for RNA-sequencing and microarray studies. *Nucleic Acids Res.* 2015;43(7):e47. 10.1093/nar/gkv007 25605792 PMC4402510

[5] Law CW Chen Y Shi W: voom: Precision weights unlock linear model analysis tools for RNA-seq read counts. *Genome Biol.* 2014;15(2):R29. 10.1186/gb-2014-15-2-r29 24485249 PMC4053721

[6] Mosimann JE: On the compound multinomial distribution, the multivariate β-distribution, and correlations among proportions. *Biometrika.* 1962;49(1–2):65–82. 10.2307/2333468

[7] Tvedebrink T: Overdispersion in allelic counts and **θ**-correction in forensic genetics. *Theor Popul Biol.* 2010;78(3):200–210. 10.1016/j.tpb.2010.07.002 20633572

[8] Chen J Li H: Variable Selection for Sparse Dirichlet-Multinomial Regression With an Application To Microbiome Data Analysis. *Ann Appl Stat.* 2013;7(1):418–442. 10.1214/12-AOAS592 24312162 PMC3846354

[9] Finak G McDavid A Chattopadhyay P: Mixture models for single-cell assays with applications to vaccine studies. *Biostatistics.* 2014;15(1):87–101. 10.1093/biostatistics/kxt024 23887981 PMC3862207

[10] Samb R Khadraoui K Belleau P: Using informative Multinomial-Dirichlet prior in a t-mixture with reversible jump estimation of nucleosome positions for genome-wide profiling. *Stat Appl Genet Mol Biol.* 2015;14(6):517–532. 10.1515/sagmb-2014-0098 26656614

[11] Mosimann JE: On the Compound Negative Multinomial Distribution and Correlations Among Inversely Sampled Pollen Counts. *Biometrika.* 1963;50(1–2):47–54. 10.1093/biomet/50.1-2.47

[12] Farewell DM Farewell VT: Dirichlet negative multinomial regression for overdispersed correlated count data. *Biostatistics.* 2013;14(2):395–404. 10.1093/biostatistics/kxs050 23221819 PMC3590929

[13] Sun D Xi Y Rodriguez B: MOABS: model based analysis of bisulfite sequencing data. *Genome Biol.* 2014;15(2):R38. 10.1186/gb-2014-15-2-r38 24565500 PMC4054608

[14] Park Y Figueroa ME Rozek LS: MethylSig: a whole genome DNA methylation analysis pipeline. *Bioinformatics.* 2014;30(17):2414–22. 10.1093/bioinformatics/btu339 24836530 PMC4147891

[15] Feng H Conneely KN Wu H: A Bayesian hierarchical model to detect differentially methylated loci from single nucleotide resolution sequencing data. *Nucleic Acids Res.* 2014;42(8):e69. 10.1093/nar/gku154 24561809 PMC4005660

[16] Wang ET Sandberg R Luo S: Alternative isoform regulation in human tissue transcriptomes. *Nature.* 2008;456(7221):470–6. 10.1038/nature07509 18978772 PMC2593745

[17] Wang GS Cooper TA: Splicing in disease: disruption of the splicing code and the decoding machinery. *Nat Rev Genet.* 2007;8(10):749–61. 10.1038/nrg2164 17726481

[18] Tazi J Bakkour N Stamm S: Alternative splicing and disease. *Biochim Biophys Acta.* 2009;1792(1):14–26. 10.1016/j.bbadis.2008.09.017 18992329 PMC5632948

[19] Hooper JE: A survey of software for genome-wide discovery of differential splicing in RNA-Seq data. *Hum Genomics.* 2014;8(1):3. 10.1186/1479-7364-8-3 24447644 PMC3903050

[20] Robinson MD McCarthy DJ Smyth GK: edgeR: a Bioconductor package for differential expression analysis of digital gene expression data. *Bioinformatics.* 2010;26(1):139–140. 10.1093/bioinformatics/btp616 19910308 PMC2796818

[21] Derti A Garrett-Engele P Macisaac KD: A quantitative atlas of polyadenylation in five mammals. *Genome Res.* 2012;22(6):1173–1183. 10.1101/gr.132563.111 22454233 PMC3371698

[22] Alamancos GP Agirre E Eyras E: Methods to study splicing from high-throughput RNA sequencing data. *Methods Mol Biol.* 2014;1126:357–397. 10.1007/978-1-62703-980-2_26 24549677

[23] Soneson C Matthes KL Nowicka M: Isoform prefiltering improves performance of count-based methods for analysis of differential transcript usage. *Genome Biol.* 2016;17(1):12. 10.1186/s13059-015-0862-3 26813113 PMC4729156

[24] Liao Y Smyth GK Shi W: FeatureCounts: an efficient general purpose program for assigning sequence reads to genomic features. *Bioinformatics.* 2014;30(7):923–930. 10.1093/bioinformatics/btt656 24227677

[25] Anders S Reyes A Huber W: Detecting differential usage of exons from RNA-seq data. *Genome Res.* 2012;22(10):2008–2017. 10.1101/gr.133744.111 22722343 PMC3460195

[26] Anders S Pyl PT Huber W: HTSeq--a Python framework to work with high-throughput sequencing data. *Bioinformatics.* 2015;31(2):166–169. 10.1093/bioinformatics/btu638 25260700 PMC4287950

[27] Ongen H Dermitzakis ET: Alternative Splicing QTLs in European and African Populations. *Am J Hum Genet.* 2015;97(4):567–575. 10.1016/j.ajhg.2015.09.004 26430802 PMC4596912

[28] Katz Y Wang ET Airoldi EM: Analysis and design of RNA sequencing experiments for identifying isoform regulation. *Nat Methods.* 2010;7(12):1009–1015. 10.1038/nmeth.1528 21057496 PMC3037023

[29] Shen S Park JW Lu ZX: rMATS: robust and flexible detection of differential alternative splicing from replicate RNA-Seq data. *Proc Natl Acad Sci U S A.* 2014;111(51):E5593–601. 10.1073/pnas.1419161111 25480548 PMC4280593

[30] Alamancos GP Pagès A Trincado JL: Leveraging transcript quantification for fast computation of alternative splicing profiles. *RNA.* 2015;21(9):1521–1531. 10.1261/rna.051557.115 26179515 PMC4536314

[31] Goldstein LD Cao Y Pau G: Prediction and Quantification of Splice Events from RNA-Seq Data. *PLoS One.* 2016;11(5):e0156132. 10.1371/journal.pone.0156132 27218464 PMC4878813

[32] Zhao K Lu ZX Park JW: GLiMMPS: Robust statistical model for regulatory variation of alternative splicing using RNA-seq data. *Genome Biol.* 2013;14(7):R74. 10.1186/gb-2013-14-7-r74 23876401 PMC4054007

[33] Jia C Hu Y Liu Y: Mapping Splicing Quantitative Trait Loci in RNA-Seq. *Cancer Inform.* 2014;13(Suppl 4):35–43. 10.4137/CIN.S13971 25452687 PMC4218654

[34] Hu Y Liu Y Mao X: PennSeq: accurate isoform-specific gene expression quantification in RNA-Seq by modeling non-uniform read distribution. *Nucleic Acids Res.* 2014;42(3):e20. 10.1093/nar/gkt1304 24362841 PMC3919567

[35] Monlong J Calvo M Ferreira PG: Identification of genetic variants associated with alternative splicing using sQTLseekeR. *Nat Commun.* 2014;5: 4698. 10.1038/ncomms5698 25140736 PMC4143934

[36] Glaus P Honkela A Rattray M: Identifying differentially expressed transcripts from RNA-seq data with biological variation. *Bioinformatics.* 2012;28(13):1721–1728. 10.1093/bioinformatics/bts260 22563066 PMC3381971

[37] Rossell D Stephan-Otto Attolini C Kroiss M: Quantifying Alternative Splicing From Paired-End RNA-Sequencing Data. *Ann Appl Stat.* 2014;8(1):309–330. 10.1214/13-AOAS687 24795787 PMC4005600

[38] Trapnell C Williams BA Pertea G: Transcript assembly and quantification by RNA-Seq reveals unannotated transcripts and isoform switching during cell differentiation. *Nat Biotechnol.* 2010;28(5):511–515. 10.1038/nbt.1621 20436464 PMC3146043

[39] Li B Dewey CN: RSEM: accurate transcript quantification from RNA-Seq data with or without a reference genome. *BMC Bioinformatics.* 2011;12:323. 10.1186/1471-2105-12-323 21816040 PMC3163565

[40] Bernard E Jacob L Mairal J: Efficient RNA isoform identification and quantification from RNA-Seq data with network flows. *Bioinformatics.* 2014;30(17):2447–2455. 10.1093/bioinformatics/btu317 24813214 PMC4147886

[41] Patro R Mount SM Kingsford C: Sailfish enables alignment-free isoform quantification from RNA-seq reads using lightweight algorithms. *Nat Biotechnol.* 2014;32(5):462–4. 10.1038/nbt.2862 24752080 PMC4077321

[42] Bray NL Pimentel H Melsted P: Near-optimal probabilistic RNA-seq quantification. *Nat Biotechnol.* 2016;34(5):525–7. 10.1038/nbt.3519 27043002

[43] Patro R Duggal G Kingsford C: Salmon: Accurate, Versatile and Ultrafast Quantification from RNA-seq Data using Lightweight-Alignment. *bioRxiv.* 2015;021592 10.1101/021592

[44] Kanitz A Gypas F Gruber AJ: Comparative assessment of methods for the computational inference of transcript isoform abundance from RNA-seq data. *Genome Biol.* 2015;16(1):150. 10.1186/s13059-015-0702-5 26201343 PMC4511015

[45] Teng M Love MI Davis CA: A benchmark for RNA-seq quantification pipelines. *Genome Biol.* 2016;17(1):74. 10.1186/s13059-016-0940-1 27107712 PMC4842274

[46] Lappalainen T Sammeth M Friedländer MR: Transcriptome and genome sequencing uncovers functional variation in humans. *Nature.* 2013;501(7468):506–11. 10.1038/nature12531 24037378 PMC3918453

[47] Battle A Mostafavi S Zhu X: Characterizing the genetic basis of transcriptome diversity through RNA-sequencing of 922 individuals. *Genome Res.* 2014;24(1):14–24. 10.1101/gr.155192.113 24092820 PMC3875855

[48] Pickrell JK Marioni JC Pai AA: Understanding mechanisms underlying human gene expression variation with RNA sequencing. *Nature.* 2010;464(7289):768–772. 10.1038/nature08872 20220758 PMC3089435

[49] Montgomery SB Sammeth M Gutierrez-Arcelus M: Transcriptome genetics using second generation sequencing in a Caucasian population. *Nature.* 2010;464(7289):773–777. 10.1038/nature08903 20220756 PMC3836232

[50] Ongen H Buil A Brown AA: Fast and efficient QTL mapper for thousands of molecular phenotypes. *Bioinformatics.* 2016;32(10):1479–85. 10.1093/bioinformatics/btv722 26708335 PMC4866519

[51] Trapnell C Hendrickson DG Sauvageau M: Differential analysis of gene regulation at transcript resolution with RNA-seq. *Nat Biotechnol.* 2013;31(1):46–53. 10.1038/nbt.2450 23222703 PMC3869392

[52] Li YI Knowles DA Pritchard JK: LeafCutter: Annotation-free quantification of RNA splicing. *bioRxiv.* 2016 10.1101/044107 PMC5742080 29229983

[53] Robinson MD Smyth GK: Moderated statistical tests for assessing differences in tag abundance. *Bioinformatics.* 2007;23(21):2881–2887. 10.1093/bioinformatics/btm453 17881408

[54] Reid N Fraser DAS: Likelihood inference in the presence of nuisance parameters.2003;7 Reference Source

[55] McCullagh P Tibshirani R: A Simple Method for the Adjustment of Profile Likelihoods. *J R Stat Soc Series B Stat Methodol.* 1990;52(2):325–344. Reference Source

[56] Cox DR Reid N: Parameter orthogonality and approximate conditional inference. *J R Stat Soc Series B Stat Methodol.* 1987;49(1):1–39. Reference Source

[57] Choi JK Kim YJ: Intrinsic variability of gene expression encoded in nucleosome positioning sequences. *Nat Genet.* 2009;41(4):498–503. 10.1038/ng.319 19252489

[58] Singh A Soltani M: Quantifying intrinsic and extrinsic variability in stochastic gene expression models. *PLoS One.* 2013;8(12):e84301. 10.1371/journal.pone.0084301 24391934 PMC3877255

[59] Brooks AN Yang L Duff MO: Conservation of an RNA regulatory map between *Drosophila* and mammals. *Genome Res.* 2011;21(2):193–202. 10.1101/gr.108662.110 20921232 PMC3032923

[60] Kim SC Jung Y Park J: A high-dimensional, deep-sequencing study of lung adenocarcinoma in female never-smokers. *PLoS One.* 2013;8(2):e55596. 10.1371/journal.pone.0055596 23405175 PMC3566005

[61] Nowicka M Robinson MD: Source code of the R package used for analyses in "DRIMSeq: a Dirichlet-multinomial framework for multivariate count outcomes in genomics" paper. *Zenodo.* 2016 Data Source 10.12688/f1000research.8900.1 PMC5200948 28105305

[62] Nowicka M Robinson MD: Source code of the analyses in the "DRIMSeq: a Dirichlet-multinomial framework for multivariate count outcomes in genomics” paper. *Zenodo.* 2016 Data Source 10.12688/f1000research.8900.1 PMC5200948 28105305

