# Supplementary material for: DRIMSeq: a Dirichlet-multinomial framework for multivariate count outcomes in genomics
Source: F1000Res. 2016 Dec 6;5:1356. Originally published 2016 Jun 13. [Version 2] doi: 10.12688/f1000research.8900.2 (PMC5200948; doi:10.12688/f1000research.8900.2)
Supplement: Supplementary file 1 [file f1000research-5-11139-s0000.tgz › 1cedc7d9-87b8-419c-a7c8-89ad590474b3.pdf]

DRIMSeq: a Dirichlet-multinomial framework for multivariate count outcomes in genomics
Supplementary Materials

Malgorzata Nowicka, Mark D. Robinson

Contents

1 Calculating the degree of dispersion moderation **2**

2 P-value adjustment with permutations in sQTL analysis **2**

3 Details on simulations from the Dirichlet-multinomial model **3**

4 Details on simulations that mimic real RNA-seq data **3**

5 Details on the differential splicing analyses **3**

5.1 Pasilla dataset 4

5.2 Adenocarcinoma dataset 4

6 Details on the tuQTL analyses **5**

7 List of abbreviations used in the Supplementary Figures **6**

Supplementary Figures **7**

Supplementary Tables **43**

References **49**

1 Calculating the degree of dispersion moderation

We propose a heuristic strategy to define the moderation level specified as W in Equations 10 and 11 in the main text.

To estimate the concentration parameter γ_+, we employ the grid approach from *edgeR* [1]. For each gene, adjusted profile likelihood is calculated for a defined set of γ_+ values, then the cubic spline function is fitted and optimized very quickly with `maximizeInterpolant` function. The set of γ_+ values spans the parameter space of potential concentration estimates creating a grid of values around the common concentration equally dividing a user defined range (Figure S21A). Figure S21B shows a profile likelihood, calculated at the grid points, for a gene that has a clear maximum within the specified γ_+ range. Figure S21C, on the other hand, shows an example of a gene with profile likelihood that cannot be optimized because it is a monotonically increasing function within the grid. In this case, the boundary grid value is used as a concentration estimate. Such behavior may take place due to the instability of likelihood function based on small number of observations. In the DS analyses considered here, there are many instances of such genes (see Figure S21A).

We use these boundary genes to estimate the minimal shrinkage W that should be applied in the weighted likelihood so that their concentration estimates become equal to the common concentration estimate. For that, we calculate the likelihood span, defined as a difference between the maximum and minimum likelihood value obtained on a grid, for the boundary genes and for the common likelihood (Figures S22A, S22D and S23A, S23D). Gene-wise likelihood APL_g in Equation 10 will be dominated by common likelihood when the span of the latter is larger than the span of the former, and the minimal level at which it happens could be approximated as a ratio between the spans of gene likelihood and common likelihood, defined here as $priorN$. Thus, $priorN$ specifies the minimal shrinkage W for each individual boundary gene. For the moderation to trended dispersion, $priorN$ is calculated similarly, using the trended likelihood instead of the common one. As a moderation level that is applied to all the genes, we decided to use one weight W for all the genes equal to the median of $priorN$ in the moderation to common dispersion, and in the moderation to trended dispersion, we use gene-wise weights W defined as a loess fitting to $priorN$ as a function of mean gene expression (Figures S22 and S23).

The number of boundary genes differs substantially between the analyses of transcript and exon counts, with many more cases on the boundary in the latter (Figures S22A, S22D and S23A, S23D). This indicates that there is much more instability in the likelihoods based on exon counts and suggesting that the DM model does not fit them well.

To assess whether our approach of moderation level W calculation is optimal, i.e., leads to improved dispersion estimation and better control of FP rate, we performed the DS analyses using different moderation levels and the one estimated by *DRIMSeq* on the data simulated from the DM model described in Section 3. In Figures S7 - S14, it is shown that the estimated shrinkage levels are very close to the one that provide best dispersion estimates and best control of FP rate.

2 P-value adjustment with permutations in sQTL analysis

The null distribution of gene-SNP associations is assessed from the analyses of permuted data. Namely, samples in transcript count table are shuffled while the genotype data and gene-SNP matching stay unchanged so that the correlation structure between fitted models is unaffected. Full and null models are fitted to such newly obtained data and p-values from the LR test are generated. This procedure is repeated a user-defined number of times (default: 10). P-values from each permutation cycle are pooled together (null p-values) and used for the adjustment of the nominal p-values. The (new) estimated p-value is equal to the fraction of null p-values that are more significant than the nominal p-value. Such permutation adjusted p-values are then corrected for multiple testing with

the Benjamini-Hochberg method using the `p.adjust` R function.

3 Details on simulations from the Dirichlet-multinomial model

In these simulations, data that corresponds to a two-group comparison with no DS was generated from the DM distribution with identical parameters in both groups.

There are slight variations between our simulations depending on their specific purpose.

The first set of simulations aimed to compare performance of the DM model using different dispersion estimates (Figures S1 - S4). Fixed feature proportions were used in the DM distribution for all the genes. In the first case, they were the same for all the features (uniform), and in the second case, they were estimated, separately for genes with a given number of features, as a median of (sorted) proportions observed in the *kallisto* counts from Kim et al. data (kim_kallisto).

In the second simulations, the goal was to see the performance of the DM model on data with different number of features and two scenarios for proportion distributions: uniform and decaying. In the uniform distribution, each feature had a proportion equal to 1/total number of features. In the decaying distribution, each following feature had a proportion of one-half smaller than the previous one, and then normalized according to the number of features (Figures S5 and S6). Both of these simulations were repeated 50 times for 1000 genes with the same expression per gene and sample.

With the third type of simulations, we wanted to assess the level of dispersion moderation (Figures from S7 to S14). They intend to better resemble a real dataset. Thus, genes have different expression, dispersion and proportions that were estimated from *kallisto* and *HTSeq* counts from the Kim et al. and Brooks et al. datasets. Gene expression was simulated using the negative-binomial distribution with mean μ generated from the log-normal distribution fitted to the observed mean gene expression and with common dispersion θ estimated by *edgeR*. Feature proportions for the DM distribution were randomly selected from the exact proportions observed for genes in one sample, and the concentration parameters γ_+ were generated from the log-normal distribution fitted to the observed concentrations. The latest simulation instance was repeated 25 times for 5000 genes each.

In these analyses, the ML estimates of concentration were obtained with the *dirmult* package version 0.1.3-4.

4 Details on simulations that mimic real RNA-seq data

Data for this comparison was obtained from the simulations by Soneson et al. [2], where all the details on data generation and accessibility are available. It includes: *HTSeq* raw (htseq) and pre-filtered (htseqprefiltered5) counts, *kallisto* raw (kallisto), filtered (kallistofiltered5) and pre-filtered (kallistoprefiltered5) counts and the *DEXSeq* results for both Drosophila melanogaster and Homo sapiens.

The *DRIMSeq* analyses were performed using Cox-Reid adjustment and common dispersion (drimseq_common) or gene-wise dispersion estimation without moderation (drimseq_genewise_grid_none), with moderation to common (drimseq_genewise_grid_common) and to trended dispersion (drimseq_genewise_grid_trended).

5 Details on the differential splicing analyses

For both of the analyses, the exonic bin counts were obtained with python scripts from *DEXSeq* version 1.10.8 (Bioconductor release 2.14) which call *HTSeq*. For the generation of flattened GTF file, `-r no` option was used, which disables aggregation of overlapping genes.

The transcript quantification was obtained with *kallisto* version 0.42.1.

We also applied our DS analysis to filtered transcript counts (kallistofiltered5), where only transcripts with expression proportions higher than 5% in at least one sample were kept, and to exonic counts (htseqprefiltered5) obtained as above with *HTSeq*, but using a GTF file where the same transcripts as for kallistofiltered5 are kept (pre-filtering approach proposed by Soneson et al. [2]).

The differential splicing analyses were done with *DRIMSeq* and *DEXSeq* version 1.10.8 for the comparisons defined in Table S2 for pasilla data and in Table S3 for adenocarcinoma data. There are two types of comparisons performed. First, the analyses that detect DS between the condition groups (model full) and second, the mock analyses, which are a comparison between the replicates of the same condition (model null). The latter aim to investigate the specificity of each method. In the mock analyses, no DS should be detected and any DS gene is treated as a FP. Thus, high number of DS genes may be interpreted as low specificity.

We used three different approaches of gene-wise dispersion estimation in *DRIMSeq*: without moderation (drimseq_genewise_grid_none) and with moderation to common (drimseq_genewise_grid_common) and to trended dispersion (drimseq_genewise_grid_trended). We did not consider common dispersion, since its performance in simulations was much worse than for the gene-wise approaches. In all situations, Cox-Reid adjustment was used. *DEXSeq* exon p-values were summarized into gene-level adjusted p-values by applying the `perGeneQValue` function.

5.1 Pasilla dataset

The data (in SRA format) was downloaded from the NCBI's Gene Expression Omnibus (GEO) under the accession number GSE18508[1] (samples from GSM461176 to GSM461182). It was converted into FASTQ format using the `fastq-dump` command from the *SRA toolkit*[2]. The reads were aligned to Drosophila melanogaster Ensembl 70 reference genome with *TopHat* version v2.0.14 where the bowtie index was generated with Bowtie 2 version 2.1.0. The reference files including:

- genome `Drosophila_melanogaster.BDGP5.70.dna.toplevel.fa.gz`[3],
- transcriptome `Drosophila_melanogaster.BDGP5.70.cdna.all.fa.gz`[4],
- gene model `Drosophila_melanogaster.BDGP5.70.gtf.gz`[5]

were downloaded from the Ensembl FTP sites. For all the details of pasilla data pre-processing, see the vignette of *PasillaTranscriptExpr* [3] package available on Bioconductor[6].

For the validation of DS analysis results, we used the 16 genes that were validated by Brooks et al. [4] with the RT-PCR for the alternative usage of exons. One of their validated cases consisted of two genes sesB and Ant2 so we decided to consider them separately.

5.2 Adenocarcinoma dataset

We downloaded the *TopHat*-mapped reads (in BAM format) from the InSilico DB[7] and used them to compute the *HTSeq* counts. The FASTQ files needed for *kallisto* were generated with `fastq-dump` from the SRA files corresponding to samples from GSM927308 to GSM927319 downloaded from GEO under the accession number GSE37764[8]. We used as a reference the Homo sapiens Ensembl 71

[1] `http://www.ncbi.nlm.nih.gov/geo/query/acc.cgi?acc=GSE18508`
[2] `http://www.ncbi.nlm.nih.gov/Traces/sra/sra.cgi?view=software`
[3] `http://ftp.ensembl.org/pub/release-70/fasta/drosophila_melanogaster/dna/`
[4] `http://ftp.ensembl.org/pub/release-70/fasta/drosophila_melanogaster/cdna/`
[5] `http://ftp.ensembl.org/pub/release-70/gtf/drosophila_melanogaster`
[6] `http://bioconductor.org/packages/PasillaTranscriptExpr`
[7] `https://insilicodb.com`
[8] `https://www.ncbi.nlm.nih.gov/geo/query/acc.cgi?acc=GSE37764`

release. The reference files including:

- genome `Homo_sapiens.GRCh37.71.dna.toplevel.fa.gz`[9],
- transcriptome `Homo_sapiens.GRCh37.71.cdna.all.fa.gz`[10],
- gene model `Homo_sapiens.GRCh37.71.gtf.gz`[11]

were downloaded from the Ensembl FTP sites.

6 Details on the tuQTL analyses

For the tuQTL analyses, expected transcript counts, obtained with Flux Capacitor, and genotype data were downloaded from the GEUVADIS project website[12]. The gene annotation[13] Release 12 (GRCh37) used in GEUVADIS analyses was download from GENCODE[14].

Originally, the genotype data is stored in the VCF files. For the analyses with *DRIMSeq* and *sQTLseekeR*, we kept only the bi-allelic SNPs with a minor allele present in at least 5 samples and at least two alleles present in a population, and converted the genotype information into 0 for ref/ref, 1 for ref/not ref, 2 for not ref/not ref, -1 or NA for missing values. Newly encoded genotypes were saved as text files. For all the details of GEUVAIDS data pre-processing, see the vignette of the *GeuvadisTranscriptExpr* [5] package available on Bioconductor[15].

The tuQTL analyses were performed with *DRIMSeq* using the Cox-Reid adjusted gene-wise dispersion without moderation and *sQTLseekeR* version 2.1 installed from GitHub[16] with default parameters in `sqtl.seeker` function. For both methods, transcript quantification was filtered to the protein coding genes that have at least 10 counts in 70 or more samples and at least two transcript left after the transcript filtering, which keeps those that have at least 10 counts and proportion of at least 5% in 5 or more samples. `sqtl.seeker` function returns non adjusted p-values. Thus, we applied to them the Benjamini and Hochberg correction for multiple testing with `p.adjust` function (the same as in *DRIMSeq*).

We compared the tuQTLs detected by *DRIMSeq* and *sQTLseekeR* for CEU and YRI populations with the associations discovered in other studies which include:

- transcript ratio QTLs (trQTLs) obtained in the GEUVADIS project [6] from the analyses of the EUR[17] (373 samples) and the YRI[18] (89 samples) populations. They provide a list of all the trQTLs and the best per gene associations (FDR = 0.05) using a cis-window of 1Mb. Notice that many of their gene-SNP pairs were not tested in our analyses since we used a cis-window of 5Kb;
- percent spliced in QTLs (psiQTLs) detected by *GLiMMPs* [7] in the analysis of the CEU (41 samples) population data from Cheung et al. [8]. They provide a list of psiQTLs (FDR = 0.1) that are closest to the target exon splice site[19].

---

[9] `http://ftp.ensembl.org/pub/release-71/fasta/homo_sapiens/dna/`
[10] `http://ftp.ensembl.org/pub/release-71/fasta/homo_sapiens/cdna/`
[11] `http://ftp.ensembl.org/pub/release-71/gtf/homo_sapiens/`
[12] `http://www.ebi.ac.uk/Tools/geuvadis-das/`
[13] `gencode.v12.annotation.gtf.gz`
[14] `http://www.gencodegenes.org/releases/12.html`
[15] `http://bioconductor.org/packages/GeuvadisTranscriptExpr`
[16] `https://github.com/jmonlong/sQTLseekeR`
[17] EUR373.trratio.cis.FDR5.all.rs137.txt and EUR373.trratio.cis.FDR5.best.rs137.txt
[18] YRI89.trratio.cis.FDR5.all.rs137.txt and YRI89.trratio.cis.FDR5.best.rs137.txt
[19] Supplementary table S1 in [7] (13059_2013_3131_MOESM2_ESM.xls)

Additionally, we consider separately the 26 psiQTLs that were validated by RT-PCR[20] and the 10 psiQTLs that were linked to GWAS signal (Table 1 in [7]). The numbers of rediscovered associations are shown in Tables S4 and S5. Figures S41 and S42 depict the data and DM estimates for the two PCR validated sQTLs that were detected by *DRIMSeq* in CEU population.

7 List of abbreviations used in the Supplementary Figures

- DM - Dirichlet-multinomial
- FP - false positive
- TPR - true positive rate
- FDR - false discovery rate
- ML - maximum likelihood
- PL - profile likelihood
- CR - Cox-Reid
- DS - differential splicing

In simulations from the DM model (Figures S1 - S12):

- n - number of samples in a condition group, for example, n=3 means that a comparison of 3 versus 3 samples was conducted
- m - total gene expression; corresponds to the number of trials in the Dirichlet-multinomial distribution
- q - number of gene features, for example, transcripts; corresponds to the number of categories in the Dirichlet-multinomial distribution
- kim - used when referring to the adenocarcinoma data from Kim et al.
- brooks - used when referring to the pasilla data from Brooks et al.

In simulations that mimic real RNA-seq data (Figures S15- S24):

- node, withde - simulations without and with differential gene expression, respectively
- nonull - simulations with differential splicing
- n - total number of genes
- nds - number of differentially spliced genes

[20] Supplementary table S3 in [7] (13059_2013_3131_MOESM4_ESM.xls)

Supplementary Figures

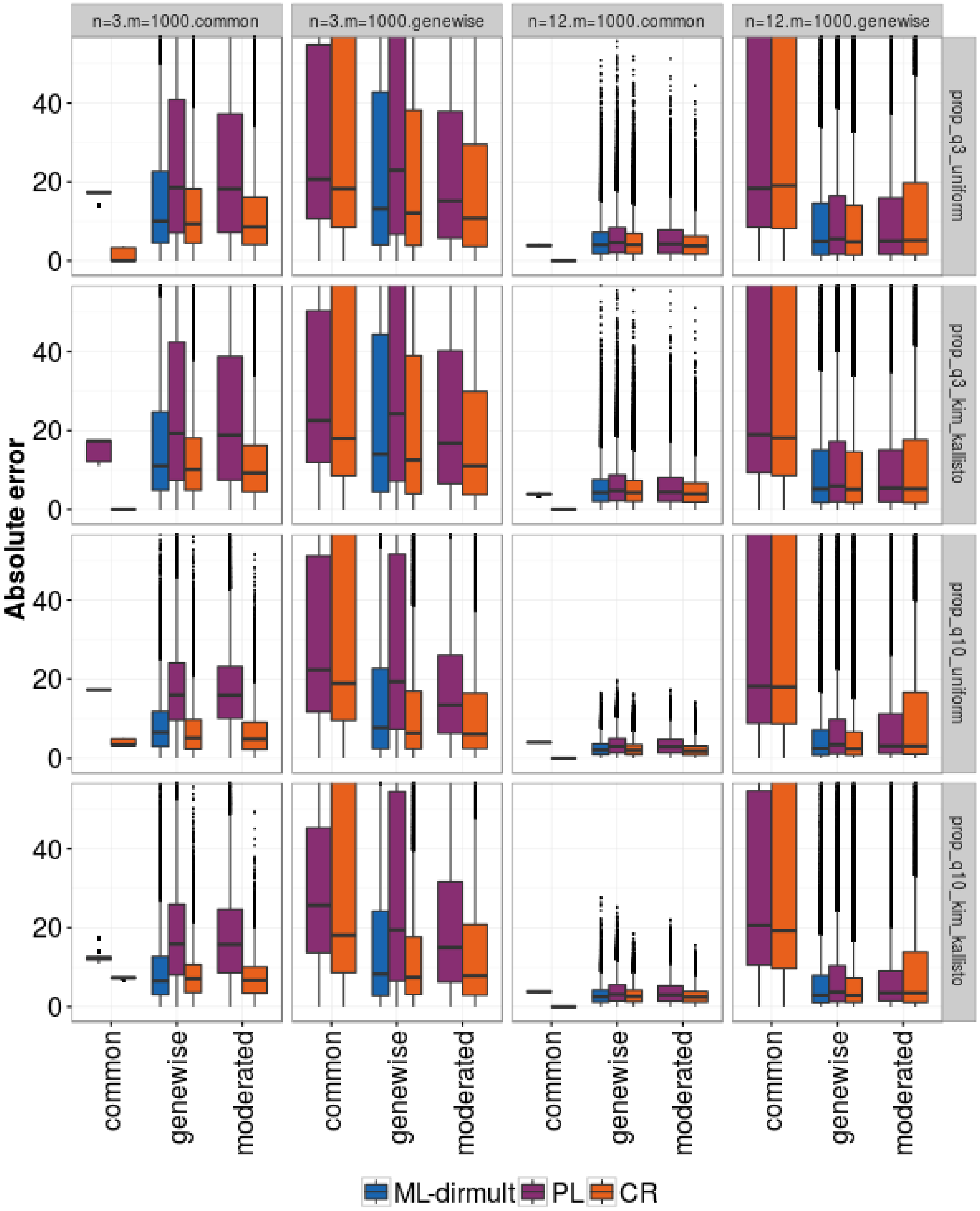

Figure S1: Simulations from the DM distribution where the aim was to compare performance of the DM model using different dispersion estimates. Data was simulated for two-group null comparison (size of each group equal to $n = 3$ or $n = 12$) with two dispersion scenarios. In the first one, all genes have the same (common) concentration, in the second one, each gene has a different (genewise) concentration. Genes have $q = 3$ or $q = 10$ features with uniform proportions or decaying proportions estimated based on *kallisto* counts of data from Kim et al., gene expression equal to $m = 1000$. For each of the scenarios, common, gene-wise, without and with moderation to common concentration was estimated. Concentration estimates were obtained with maximum likelihood using the *dirmult* package (ML-dirmult), the raw profile likelihood (PL) and the Cox-Reid adjusted profile likelihood (CR). The Y-axis shows the median absolute error of concentration γ_+ estimates.

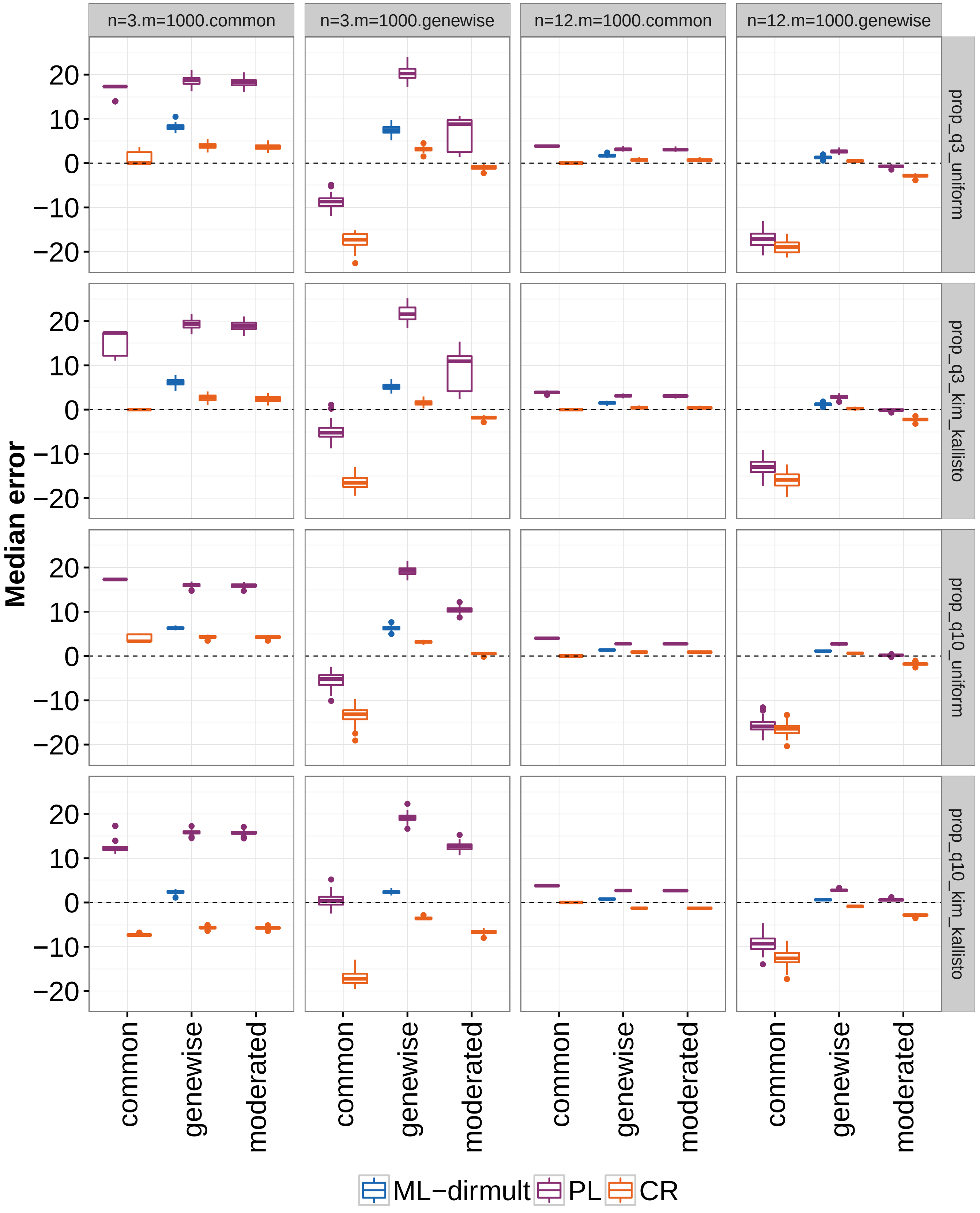

Figure S2: Simulations from the DM distribution where the aim was to compare performance of the DM model using different dispersion estimates. Median raw error of concentration γ_+ estimates are shown.

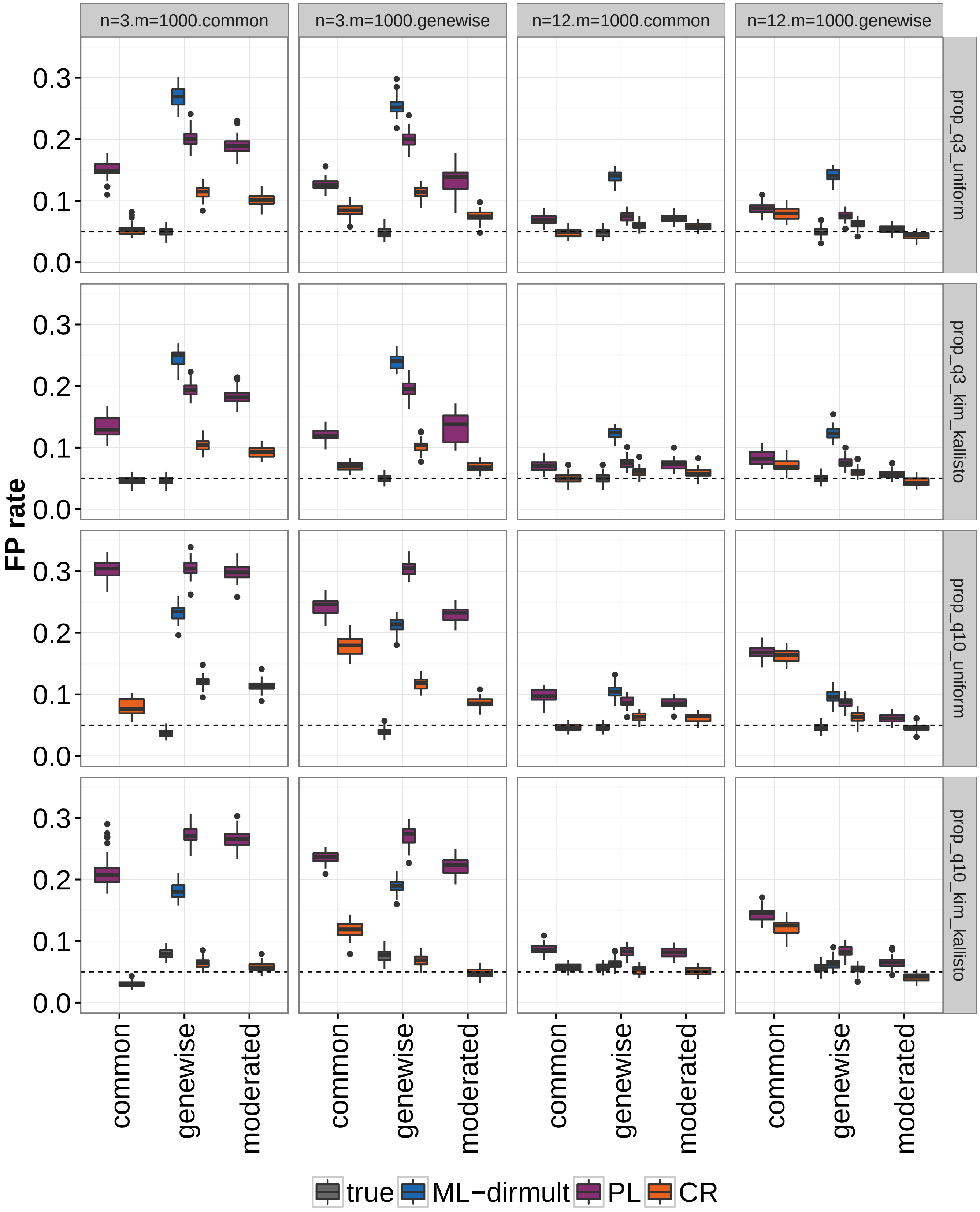

Figure S3: Simulations from the DM distribution where the aim was to compare performance of the DM model using different dispersion estimates. False positive (FP) rate for the p-value threshold of 0.05 of the null two-group comparisons based on the likelihood ratio (LR) statistics. Additionally, the FP rates when true concentration estimates were used for the inference are shown as gray boxplots.

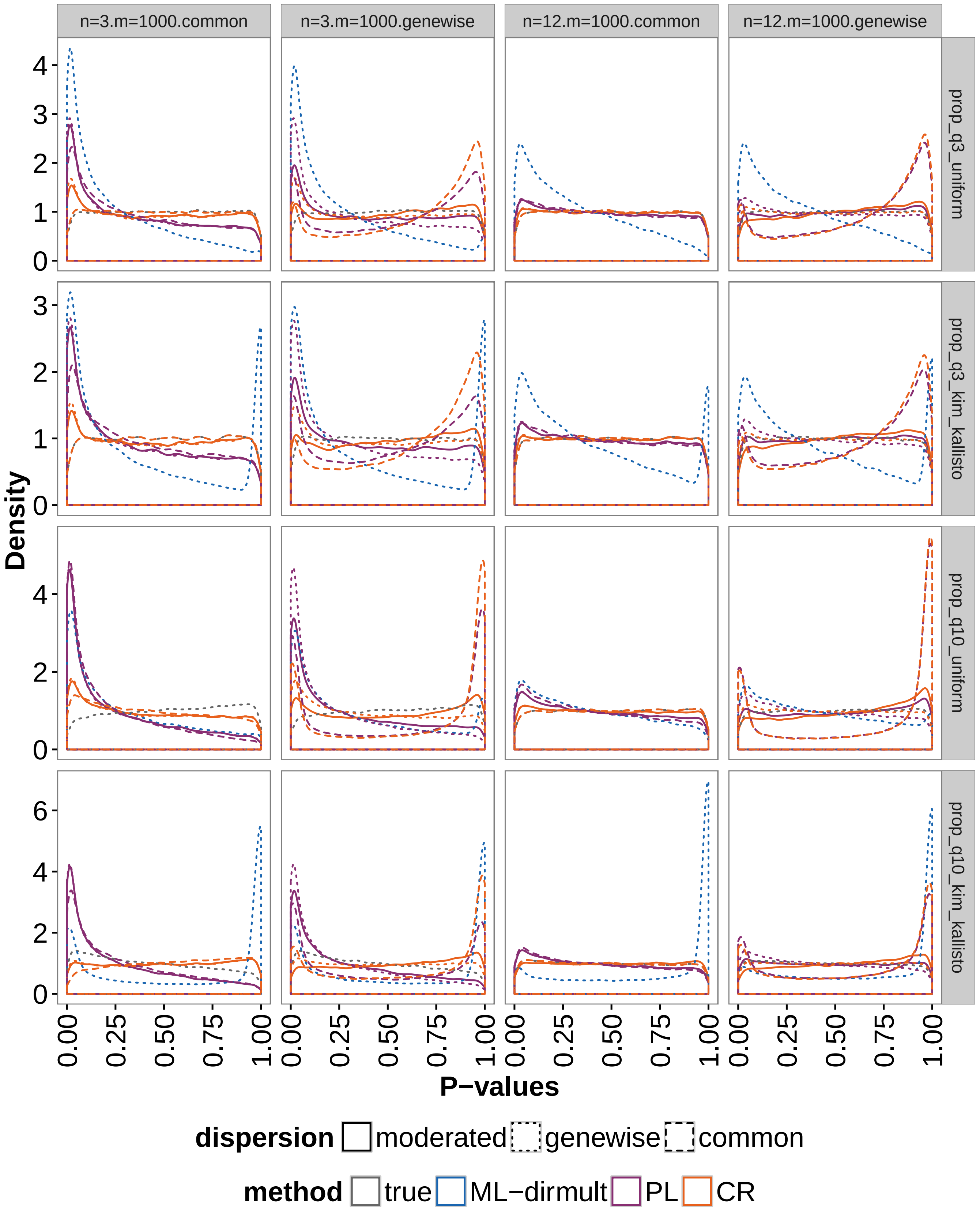

Figure S4: Simulations from the DM distribution where the aim was to compare performance of the DM model using different dispersion estimates. P-value distributions of the null two-group comparisons based on the likelihood ratio (LR) statistics.

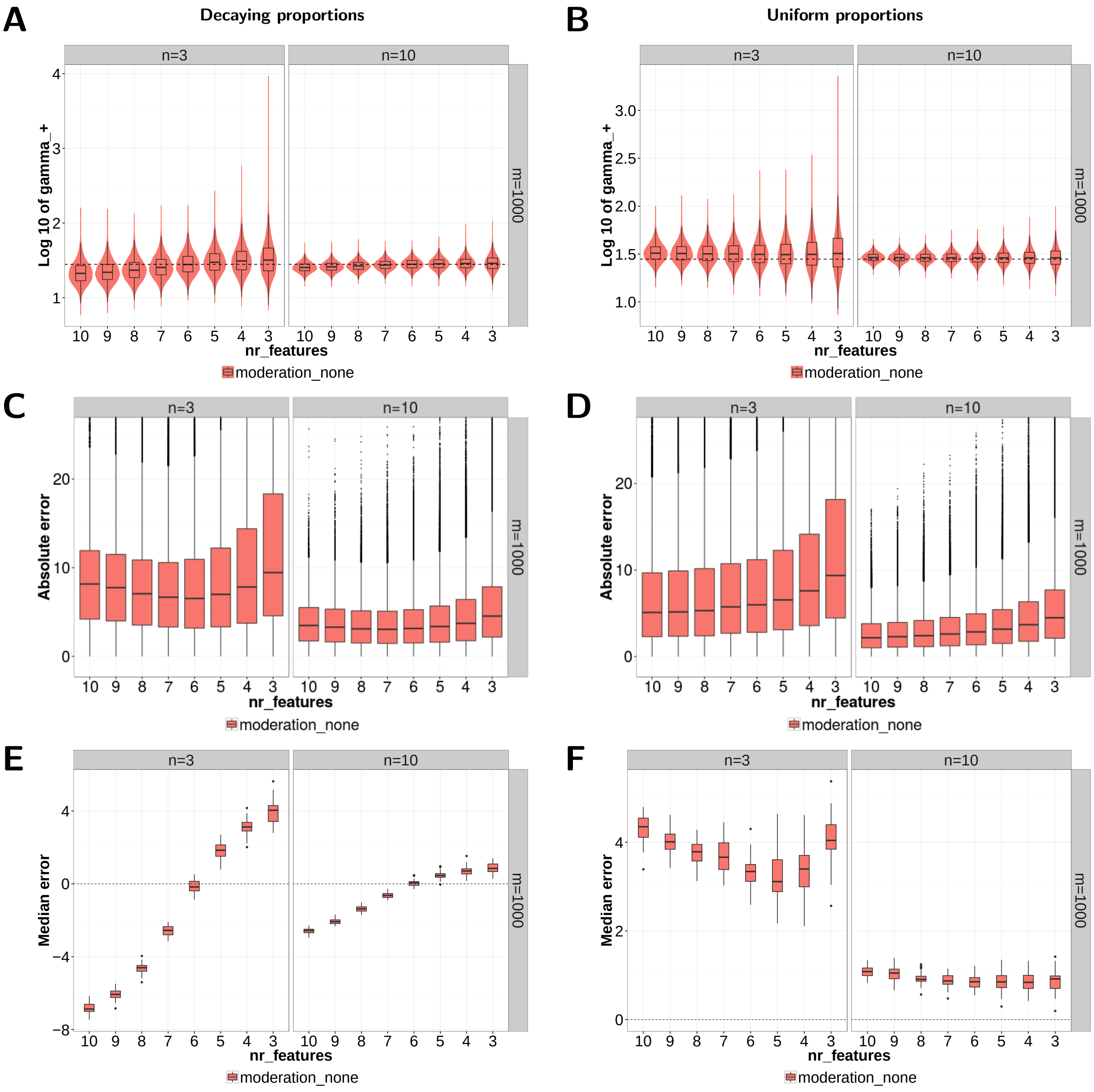

Figure S5: Simulations from the DM distribution where the aim was to compare performance of the DM model using different distributions for the feature proportions. *DRIMSeq* results of the gene-wise dispersion estimation (no moderation) based on the Cox-Reid adjusted profile likelihood where genes are simulated to have identical expression (m=1000) and dispersion but different number of features (nr_features) with decaying (left panels) and uniform (right panels) proportions in comparisons of 3 versus 3 (n=3) and 10 versus 10 (n=10) samples. A, B: Concentration estimates as violin plots and the true concentration marked with dashed line. C, D: Absolute error of the concentration estimates. E,F: Median error of the concentration estimates. For genes with more features and decaying proportions, concentration is more underestimated (overestimation of dispersion) and vice versa for genes with uniform proportions.

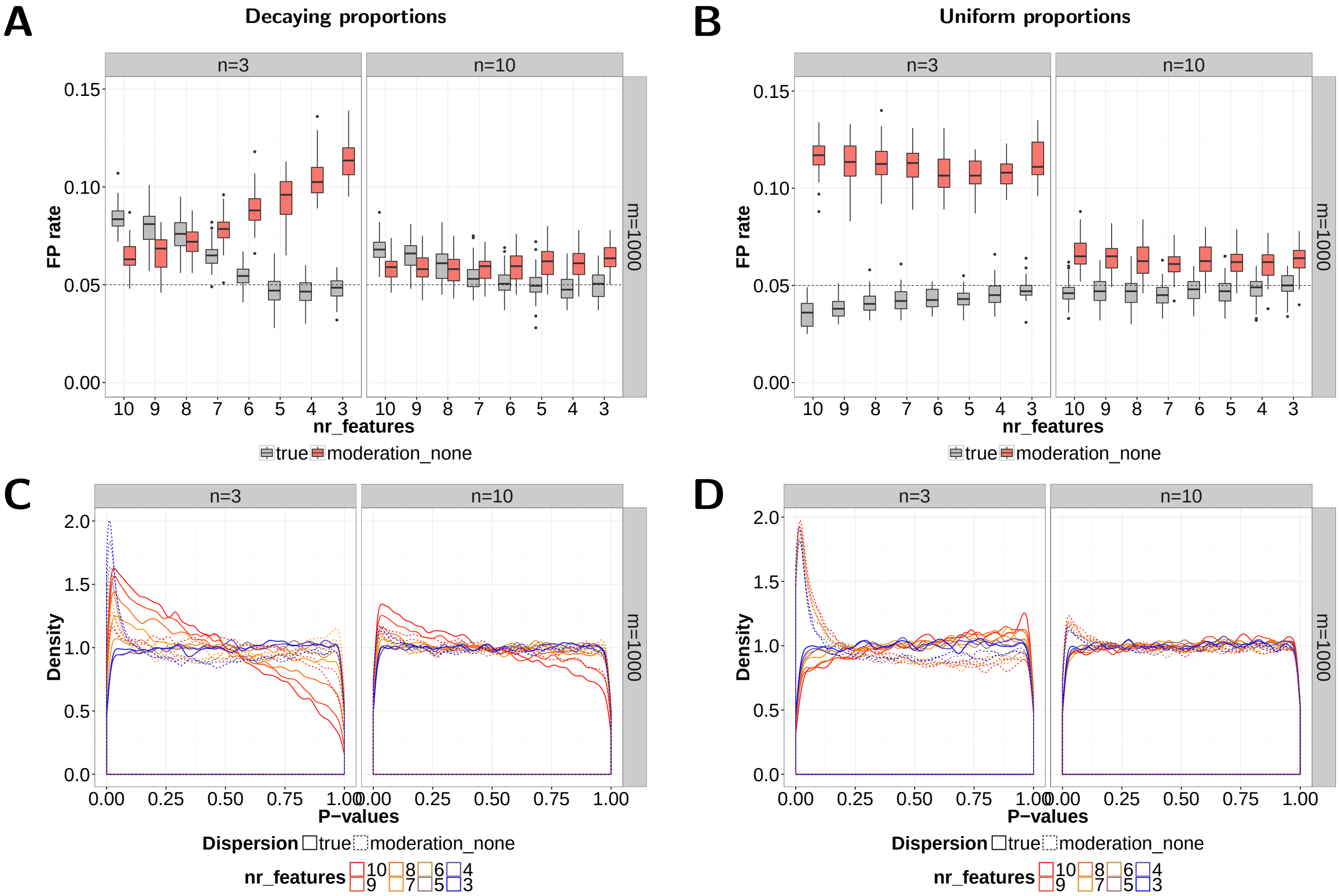

Figure S6: Simulations from the DM distribution where the aim was to compare performance of the DM model using different distributions for the feature proportions. Left panels: features with decaying proportions. Right panels: features with uniform proportions. A, B: False positive (FP) rates. C, D: Distributions of p-values. The FP rate decreases as genes have more features with decaying proportions when the estimated (moderation_none) dispersion is used and increases for true dispersion. For the uniform proportions, the FP rate stays more or less at the same level despite the number of features.

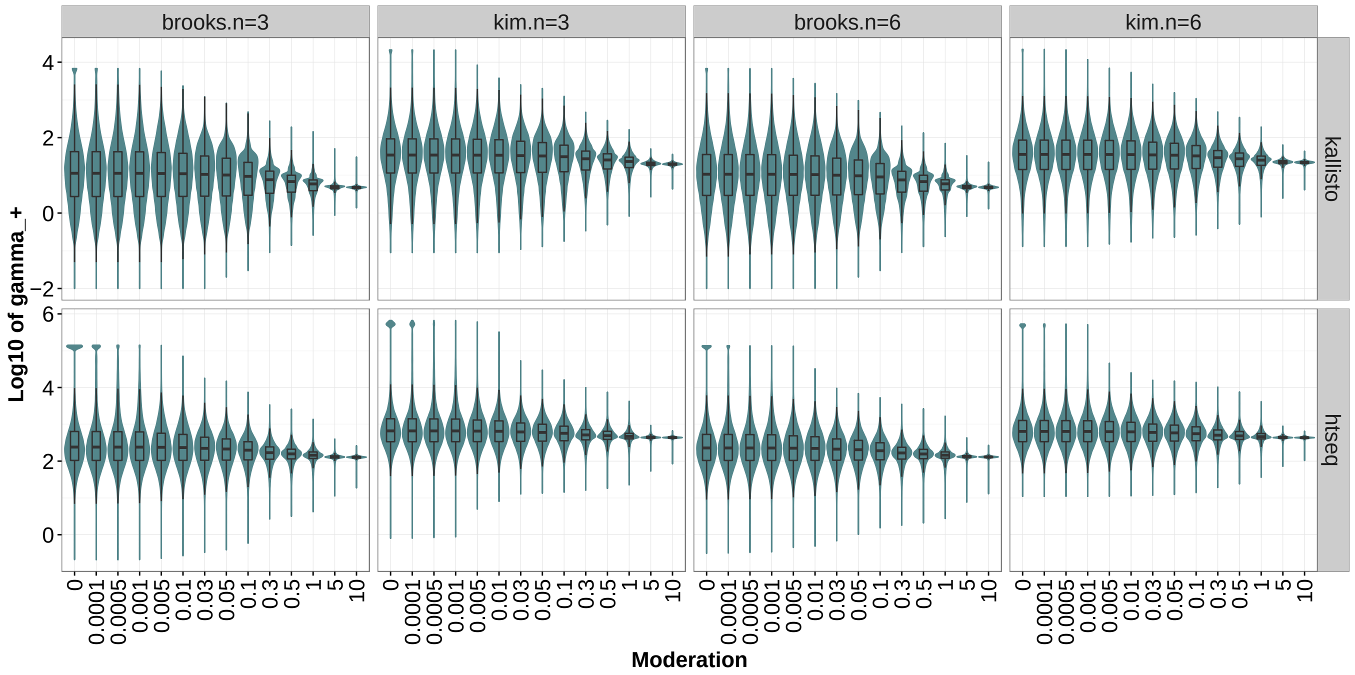

Figure S7: Simulations from the DM distribution where the aim was to assess the dispersion moderation level. Genes were simulated to have different expression, dispersion and proportions as estimated using *kallisto* and *HTSeq* counts from Kim et al. and Brooks et al. datasets. *DRIMSeq* was applied with different level of moderation (x-axis) when estimating moderated to common gene-wise dispersion. On the y-axis are the estimates of concentration γ_+. When no or low moderation is applied, concentration for some genes is estimated as a boundary value (bubble on the top of violin). Applying moderation shrinks these boundary values. As the moderation level increases, concentration estimates are shrunk more and more to the common value.

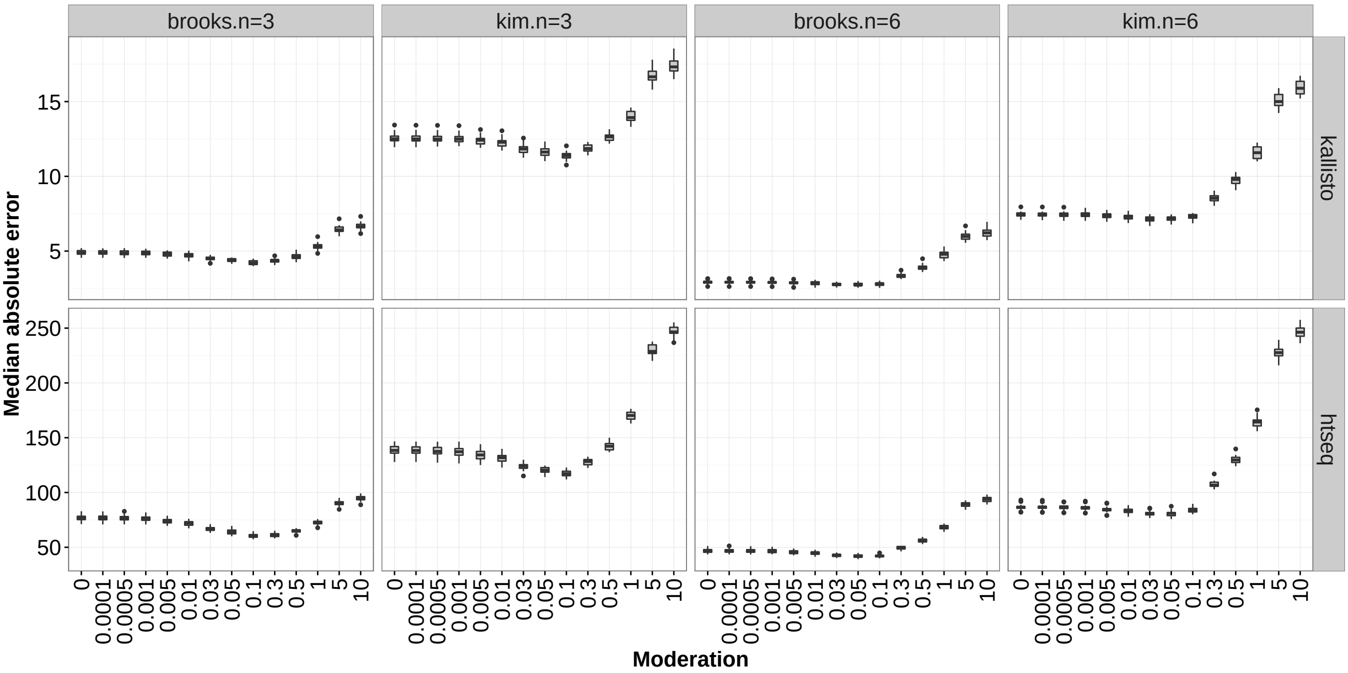

Figure S8: Simulations from the DM distribution where the aim was to assess the dispersion moderation level. Median absolute error of concentration estimates.

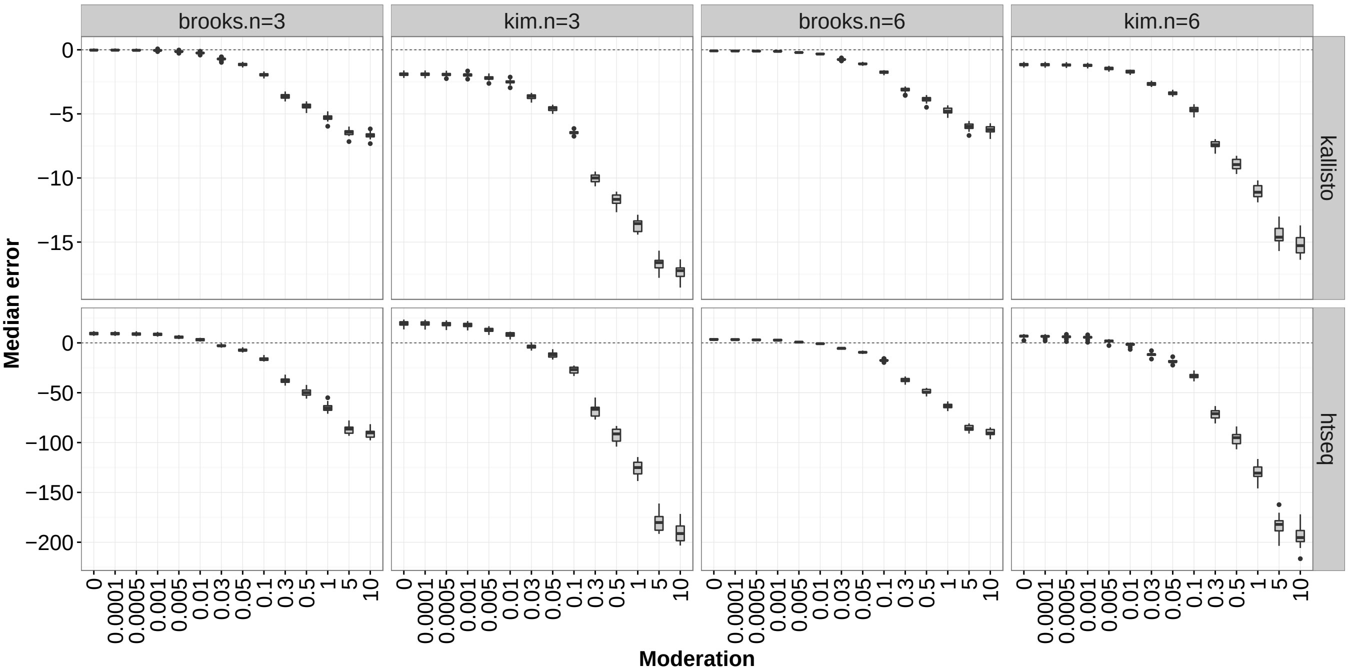

Figure S9: Simulations from the DM distribution where the aim was to assess the dispersion moderation level. Median error of concentration estimates.

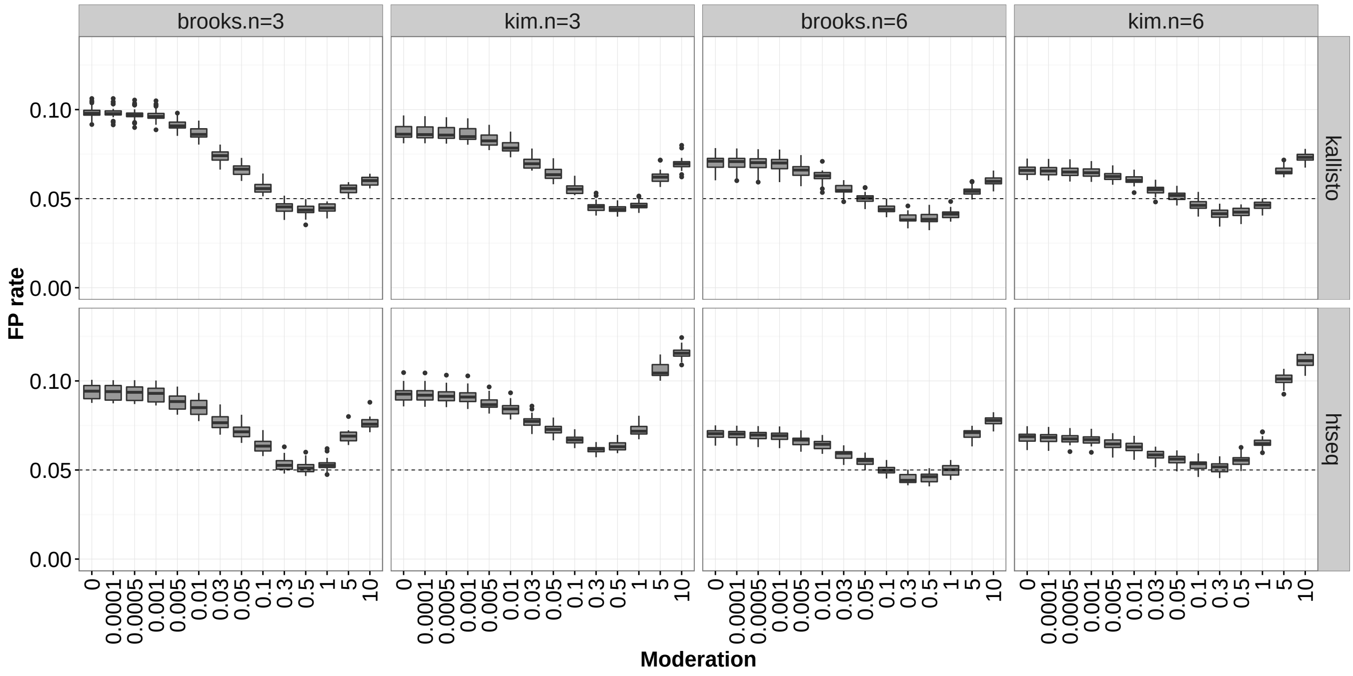

Figure S10: Simulations from the DM distribution where the aim was to assess the dispersion moderation level. FP rate obtained when conducting the DS inference with moderated dispersion.

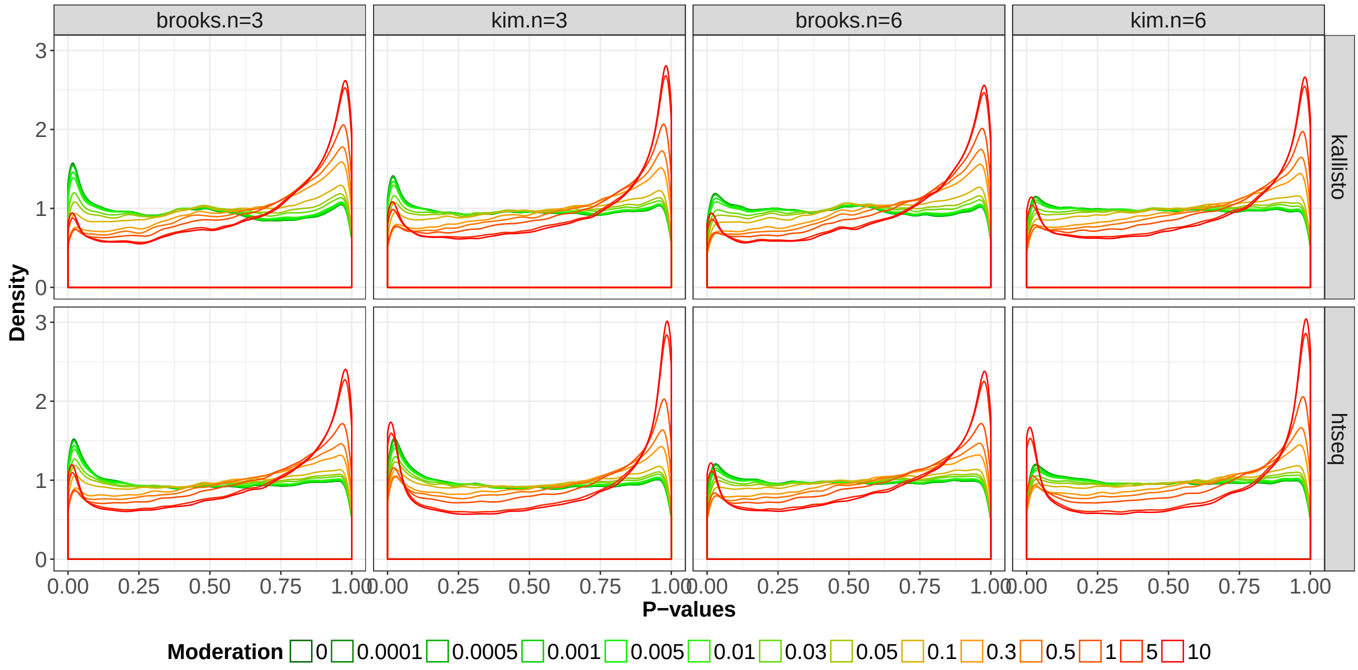

Figure S11: Simulations from the DM distribution where the aim was to assess the dispersion moderation level. Distributions of p-values obtained when conducting the DS inference with moderated dispersion.

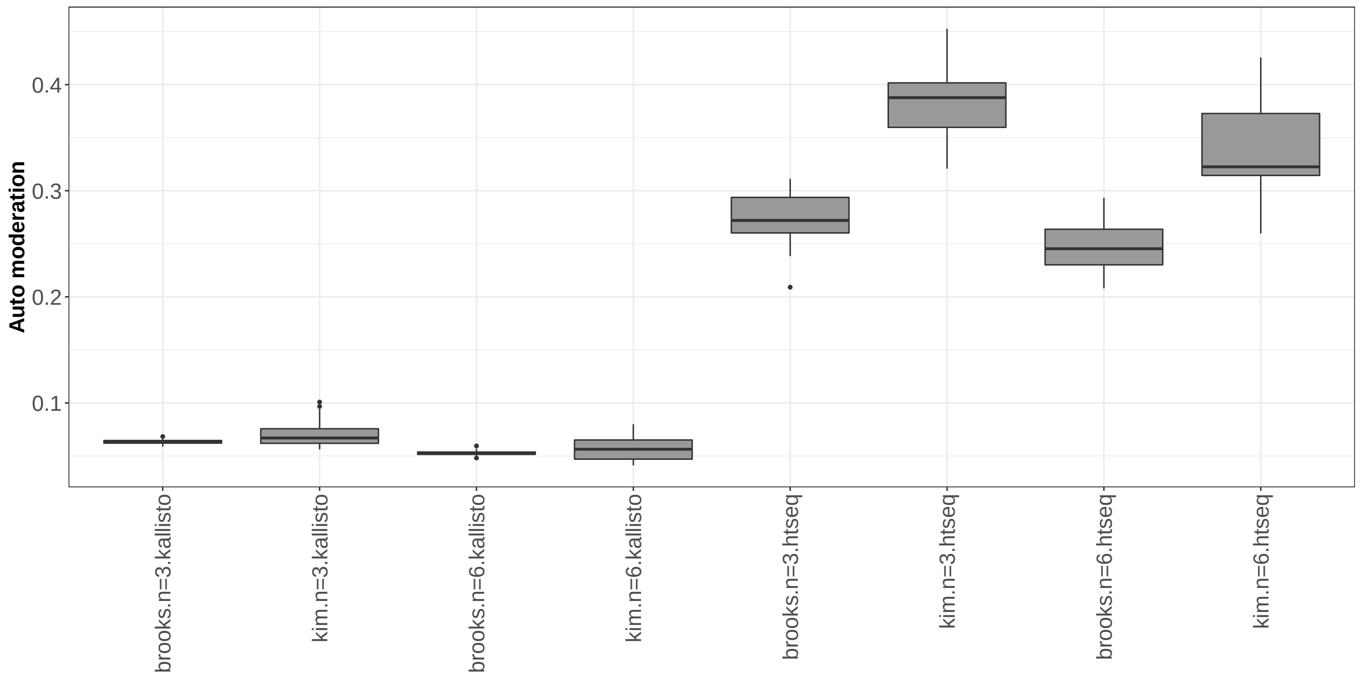

Figure S12: Simulations from the DM distribution where the aim was to assess the dispersion moderation level. Automatically estimated moderation levels. In order to shrunk the boundary concentration estimates, more moderation is needed for the exonic (*HTSeq*) than transcript (*kallisto*) counts. As the sample size increases, less moderation is required.

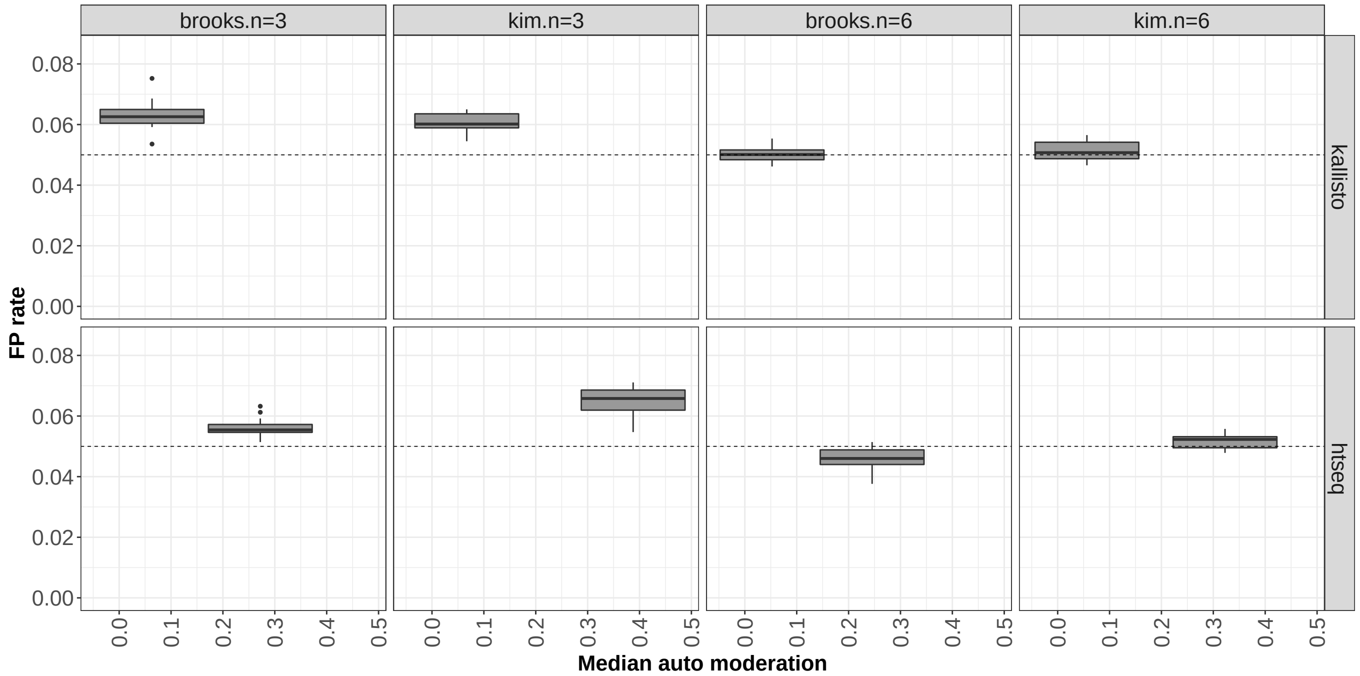

Figure S13: Simulations from the DM distribution where the aim was to assess the dispersion moderation level. FP rate corresponding to the automatically estimated moderation levels.

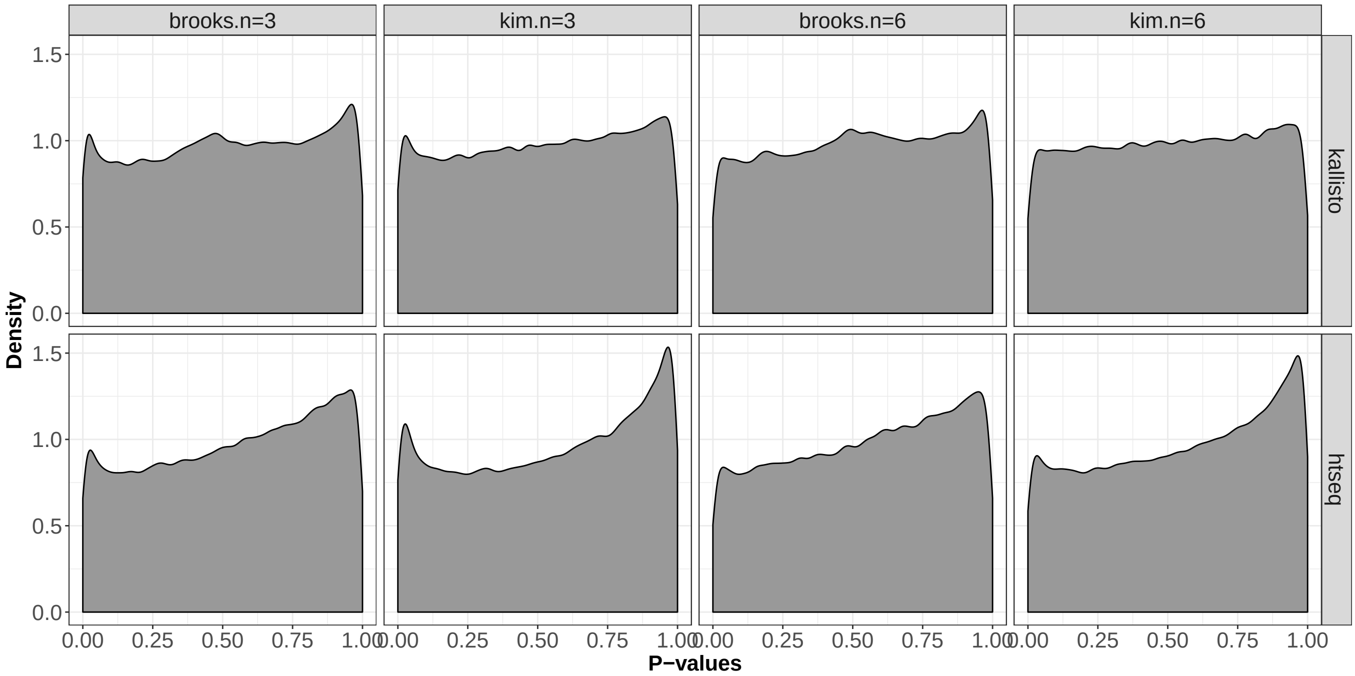

Figure S14: Simulations from the DM distribution where the aim was to assess the dispersion moderation level. Distributions of p-values obtained when conducting the DS inference with moderated dispersion, where the levels of moderation was estimated automatically.

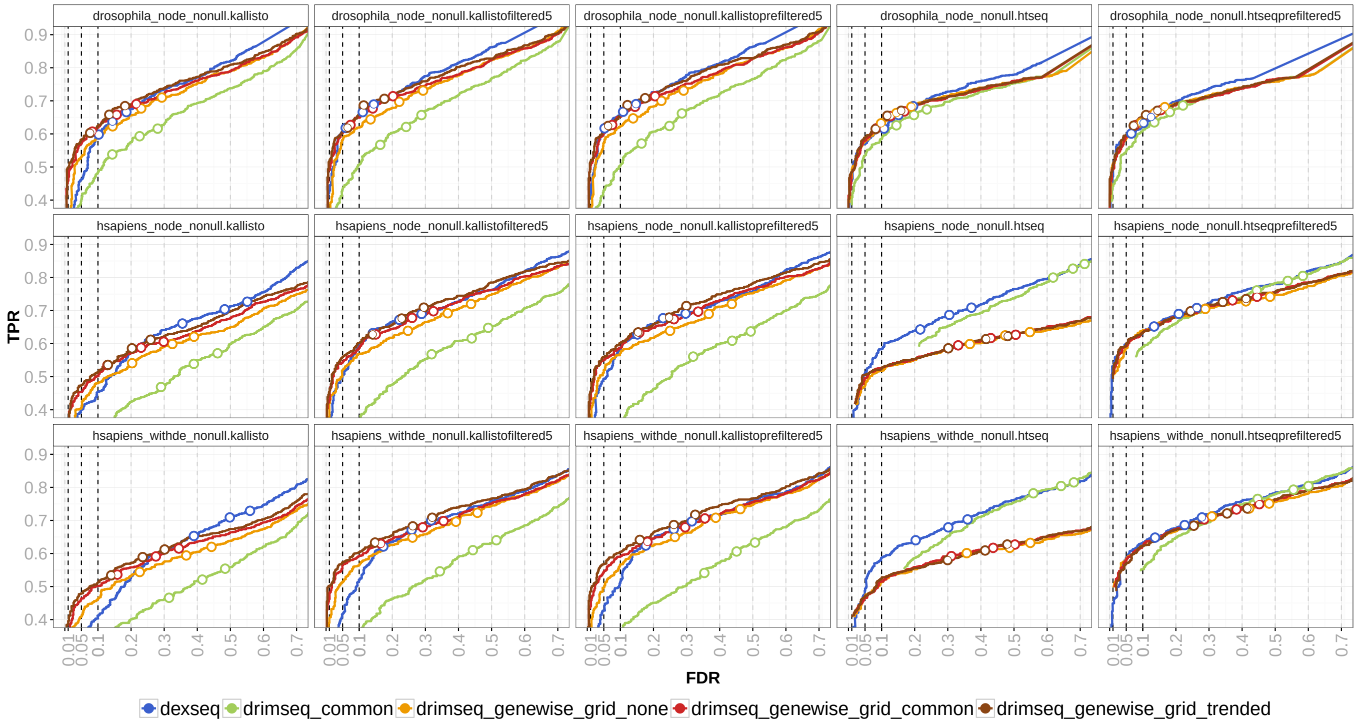

Figure S15: True positive rate (TPR) versus achieved false discovery rate (FDR) for three FDR thresholds (0.01, 0.05 and 0.1) obtained by *DEXSeq* and *DRIMSeq* with different dispersion estimation strategies: common dispersion and genewise dispersion with no moderation (genewise_grid_none), moderation to common dispersion (genewise_grid_common) and moderation to trended dispersion (genewise_grid_trended). Results presented for simulations of Drosophila melanogaster and Homo sapiens, both with no differential gene expression (node), and Homo sapiens with differential gene expression (withde). All cases with differential splicing (nonull). Transcript counts from *kallisto*, exonic counts from *HTSeq*, pre-filtered counts (kallistoprefiltered5, htseqprefiltered5) and simply filtered *kallisto* counts (kallistofiltered5) were used. When the achieved FDR is smaller than the threshold, circles are filled with the corresponding color, otherwise, they are white. There is not too much difference between kallisto filtered or pre-filtered counts, indicating that here it was not necessary to recalculate the transcript abundance based on the reduced GTF. Method performance is almost identical for H. sapiens with and without DGE, showing that both approaches accurately account for gene expression.

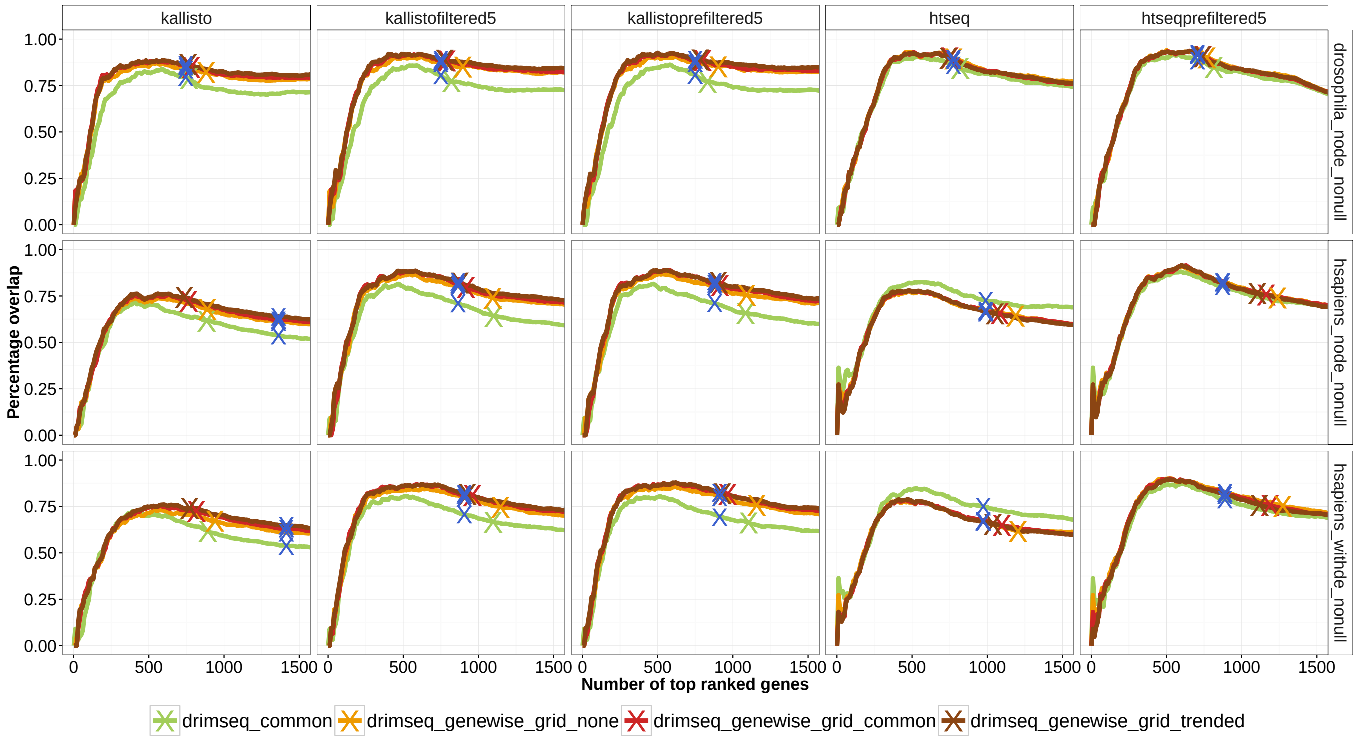

Figure S16: Concordance between *DEXSeq* and different versions of *DRIMSeq* for the top significant DS genes detected by each of the methods. "X" indicates the number of genes detected as DS for the FDR of 0.05. Blue "X" corresponds to *DEXSeq*.

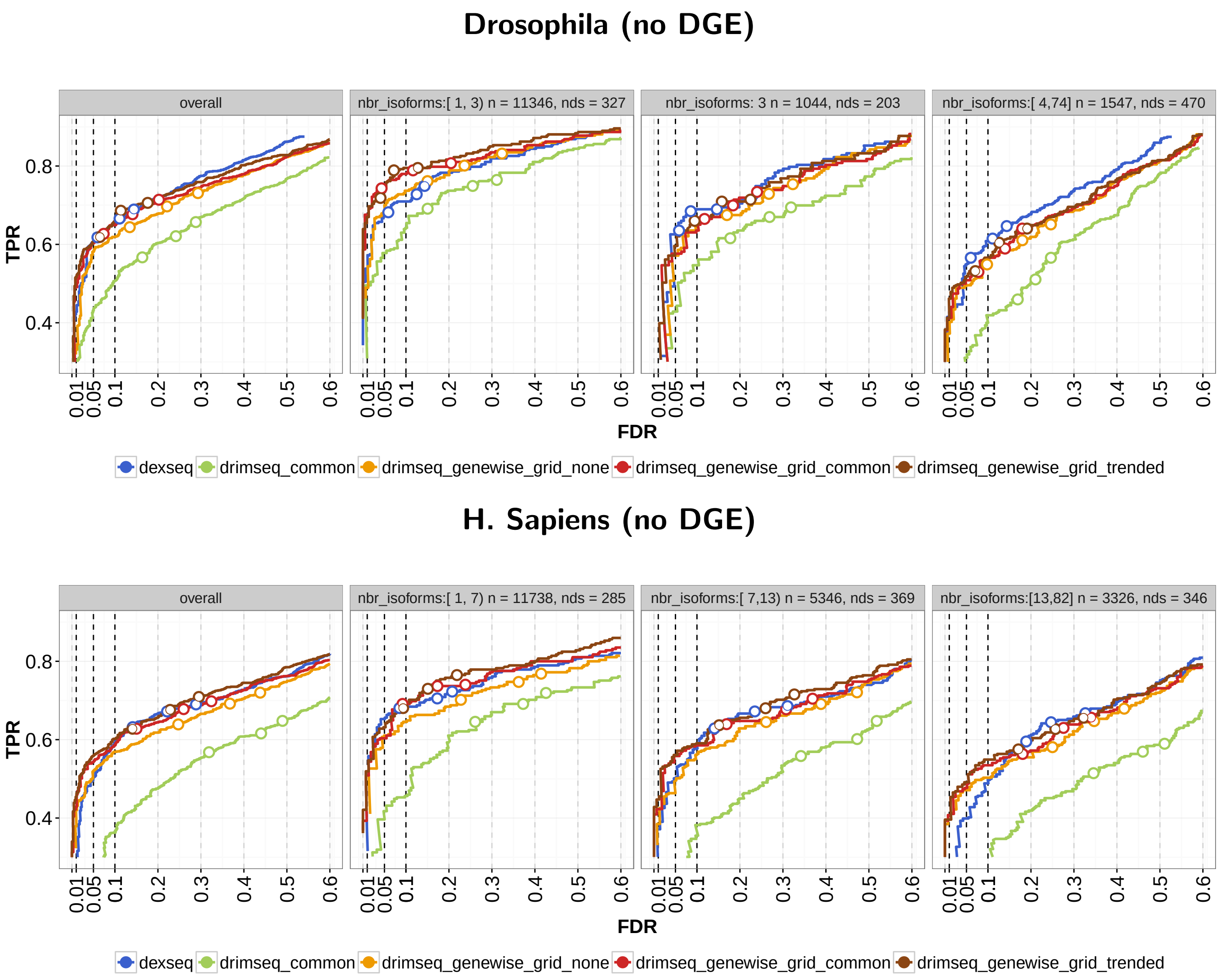

Figure S17: TPR and FDR stratified by the number of isoforms for the analysis based on *kallisto* filtered counts. The ability to control the FDR at an imposed level and the TPR depend on the number of isoforms of the genes. The FDR control for genes with many isoforms is worse and TPR is smaller than that for genes with few isoforms. Number of isoforms in the brackets (e.g., [1,3) for Drosophila), the total number of genes (n) and the number of DS genes (nds) in each category are indicated in the panel headers.

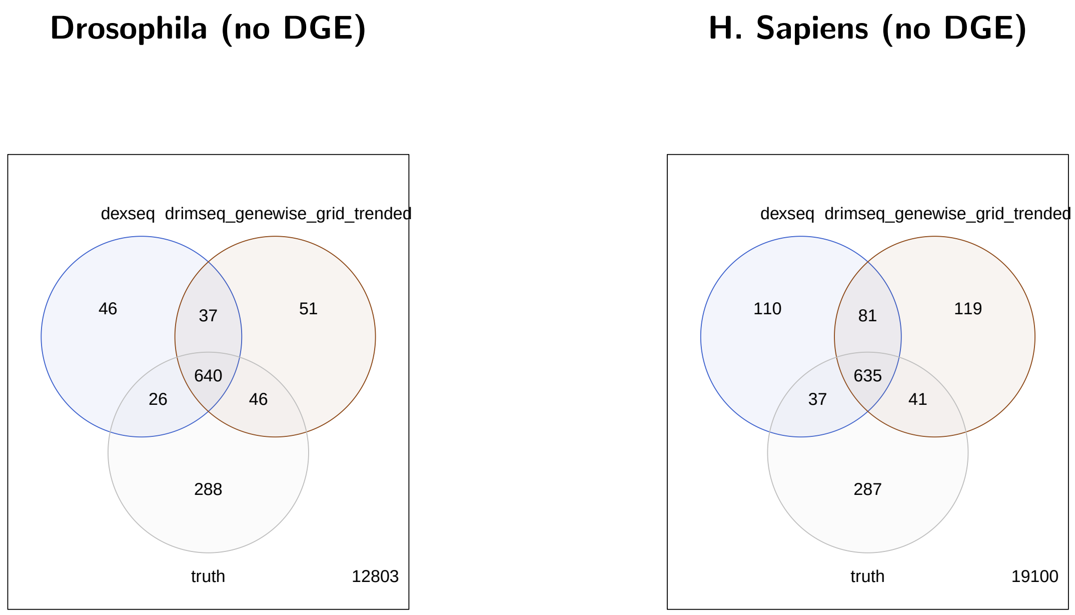

Figure S18: Numbers of DS genes detected by *DEXSeq* and *DRIMSeq* (with gene-wise dispersion moderated to the trend) at the FDR = 0.05 based on the *kallisto* filtered counts. Truth corresponds to the genes with imposed DTU.

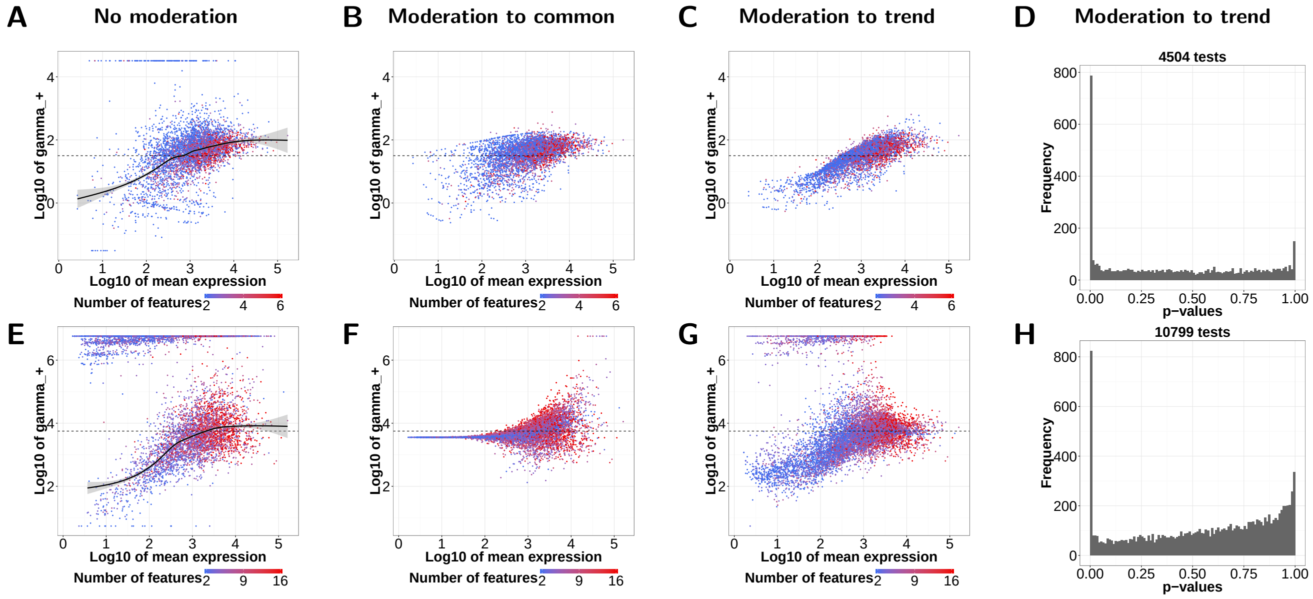

Figure S19: Results of the Drosophila (no DGE) analyses with *DRIMSeq*. Upper panels - *kallisto* counts, bottom panels - *HTSeq* counts. A, B, C, E, F, G: Concentration versus mean gene expression plots using different dispersion estimation approaches. Dashed line corresponds to the common dispersion estimate. Additionally, when no moderation is used, a smoothed curve is fitted. The fitting clearly indicates that there is a dispersion-mean trend present in the data. D, H: P-value histograms for trended dispersion used in the inference. For *kallisto* counts, the p-value distribution is more uniform with a sharp peak close to zero, which suggests a better fit of the DM model to transcript counts than to exonic counts.

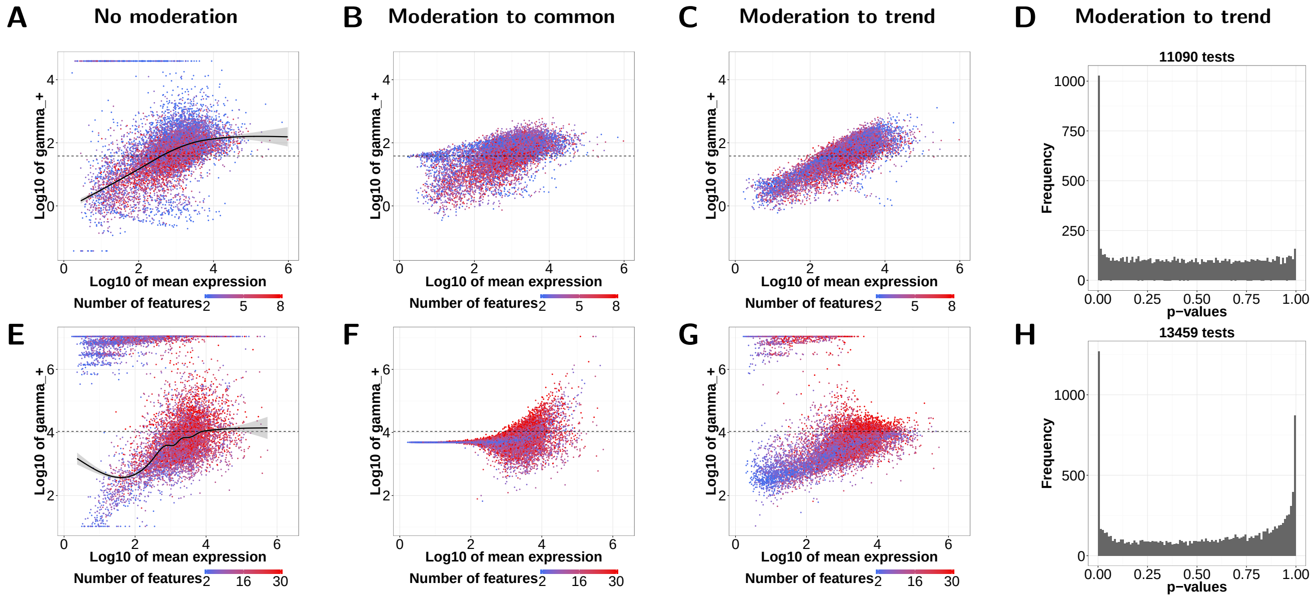

Figure S20: Results of the Homo sapiens (no DGE) analyses with *DRIMSeq*. Upper panels - *kallisto* counts, bottom panels - *HTSeq* counts. A, B, C, E, F, G: Concentration versus mean gene expression plots using different dispersion estimation approaches. Dashed line corresponds to the common dispersion estimate. Additionally, when no moderation is used, a smoothed curve is fitted. The fitting clearly indicates that there is a dispersion-mean trend present in the data. D, H: P-value histograms for trended dispersion used in the inference. For *kallisto* counts, the p-value distribution is more uniform with a sharp peak close to zero, which suggests a better fit of the DM model to transcript counts than to exonic counts.

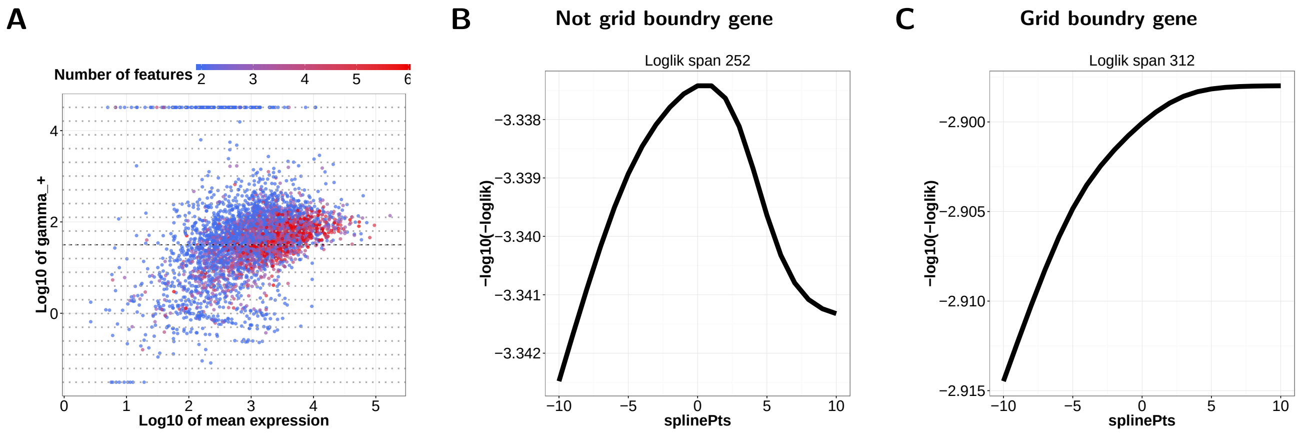

Figure S21: A: Concentration versus mean gene expression with marked grid points (gray dashed lines). B, C: Profile likelihood (y-axis) along the grid points (x-axis). B: Example of a gene with profile likelihood that can be maximized within the grid. C: Example of a gene with profile likelihood that is a monotone increasing function over the grid and the concentration estimate is equal to the boundary value. Data from the Drosophila (no DGE) analysis based on *kallisto* filtered counts.

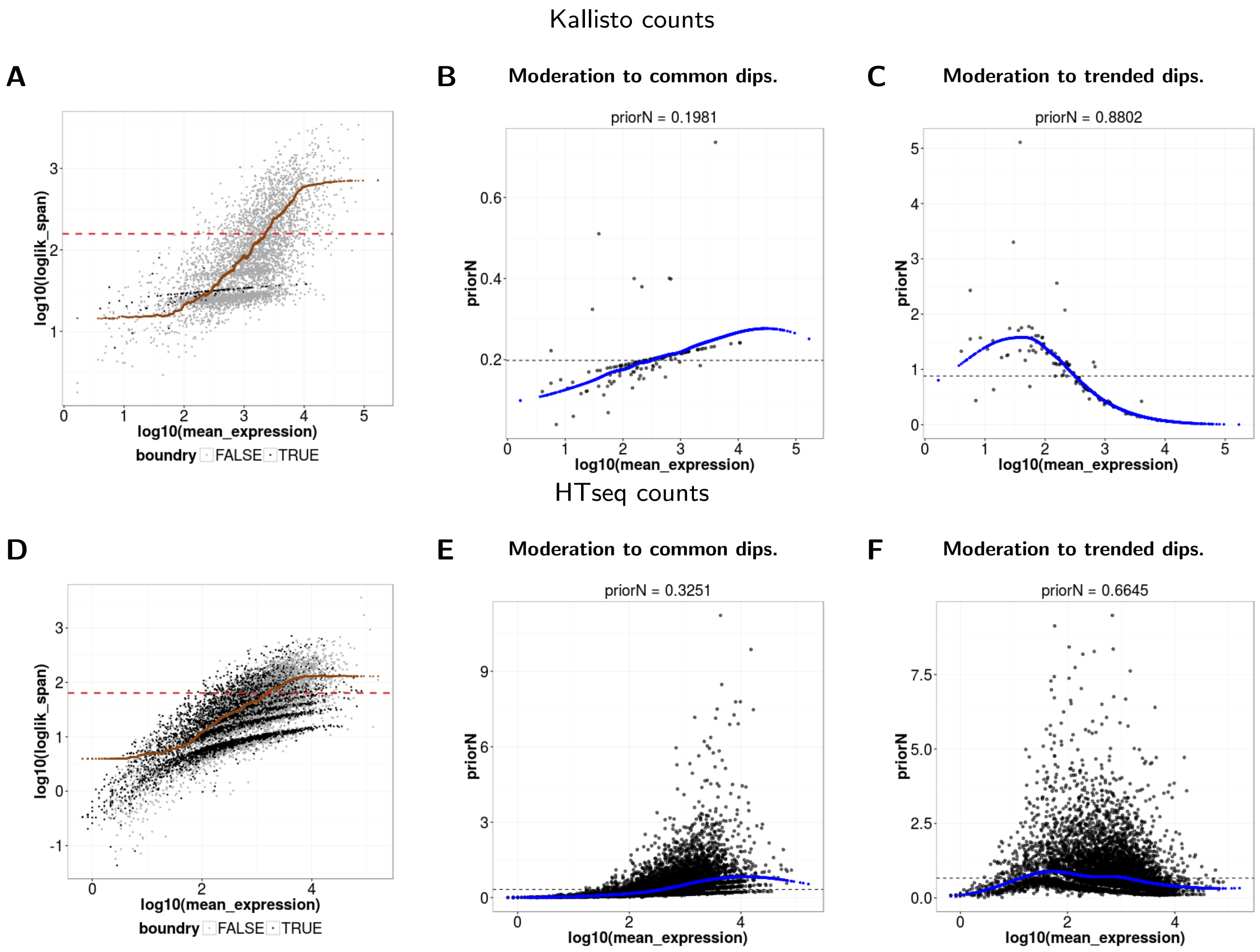

Figure S22: Results for Drosophila (no DGE). A, D: Profile likelihood span versus mean gene expression. A likelihood span is defined as difference between the maximum and minimum value of a likelihood calculated over the grid of potential concentration estimates. Black dots indicate span for the grid boundary genes, gray points the rest of the genes. Red dashed line corresponds to a span of common likelihood and brow points to the trended likelihood. B, C, E, F: priorN versus mean gene expression, where priorN is a ratio between the likelihood span of boundary genes and the span of common likelihood (B, E) or trended likelihood (C, F). Dashed line corresponds to the median of observed priorN, which is used as a moderation level W for shrinkage to the common dispersion, blue line is a loess fitting to the data and is used as a moderation level in shrinkage to the trended dispersion. There are many more boundary genes when exonic counts are used, which can suggest that the DM model does not explain this type of data so well.

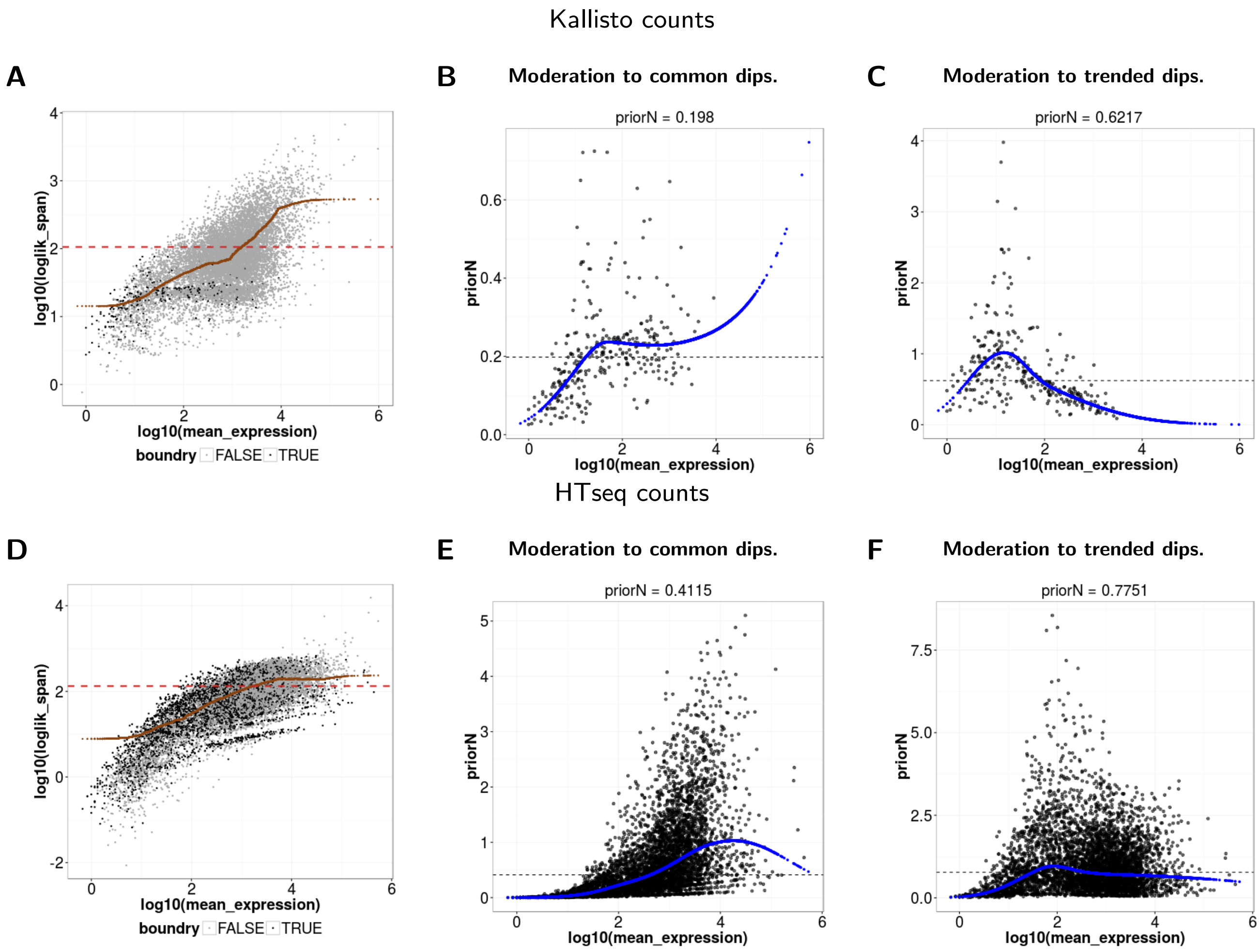

Figure S23: Results for Homo sapiens (no DGE). A, D: Profile likelihood span versus mean gene expression. A likelihood span is defined as difference between the maximum and minimum value of a likelihood calculated over the grid of potential concentration estimates. Black dots indicate span for the grid boundary genes, gray points the rest of the genes. Red dashed line corresponds to a span of common likelihood and brow points to the trended likelihood. B, C, E, F: priorN versus mean gene expression, where priorN is a ratio between the likelihood span of boundary genes and the span of common likelihood (B, E) or trended likelihood (C, F). Dashed line corresponds to the median of observed priorN, which is used as a moderation level W for shrinkage to the common dispersion, blue line is a loess fitting to the data and is used as a moderation level in shrinkage to the trended dispersion. There are many more boundary genes when exonic counts are used, which can suggest that the DM model does not explain this type of data so well.

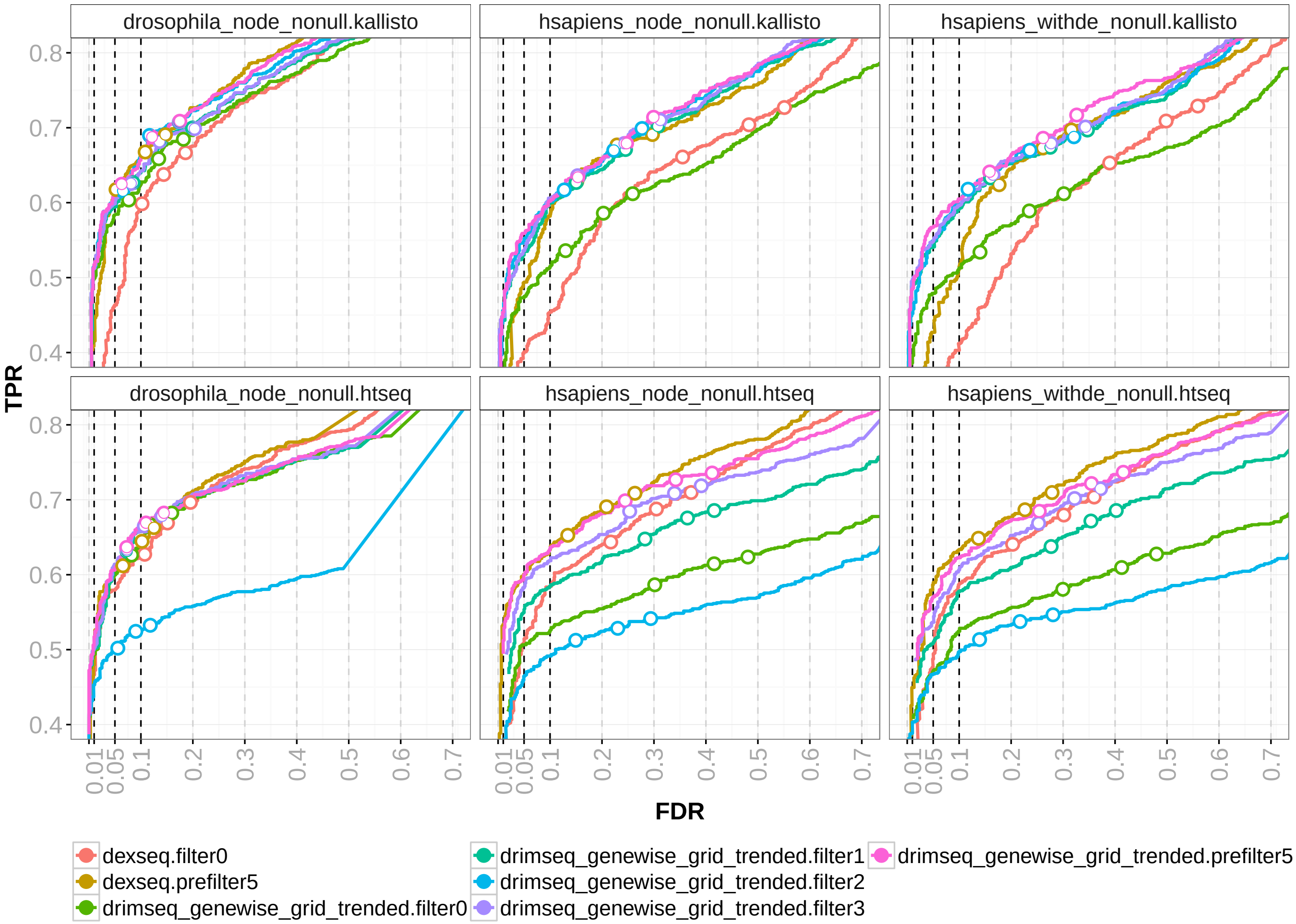

Figure S24: TPR versus FDR for *DEXSeq* and *DRIMSeq* with gene-wise dispersion moderated to the trend with different filtering approaches applied to *kallisto* and *HTSeq* counts. filter0 - corresponds to no filtering, i.e., all features (exonic bins or transcripts) with at least one count in one sample are kept. filter1 - filters to genes with at least 10 counts in all the 6 samples and features with with at least 10 counts in 3 and more samples. filter2 - keeps all the features that are expressed at the ratio of 5% in at least one sample. filter3 - additionally to the conditions from filter1, requires that a feature is expressed at the ratio of at least 0.5% in 3 and more samples. prefilter5 - is a pre-filtering approach proposed by Soneson et al., where only the transcripts with expression ratio of at least 5% in 1 and more samples, based on *kallisto* counts, are kept in the annotation. Such reduced annotation is then used to re-compute the *HTSeq* and *kallisto* counts. Filtering approaches proposed here affect exon results with different degree, while transcript results based on filtered data are very similar. For transcript counts, applying filtering increases substantially the power of *DRIMSeq* but the FDR is also elevated, for *DEXSeq* filtering mostly reduces its FDR and the TPR stays at the very similar level. In the analyses of exonic counts, pre-filtering approach is the most effective one it increases the TPR and decreases the FDR (the latter especially for *DEXSeq*).

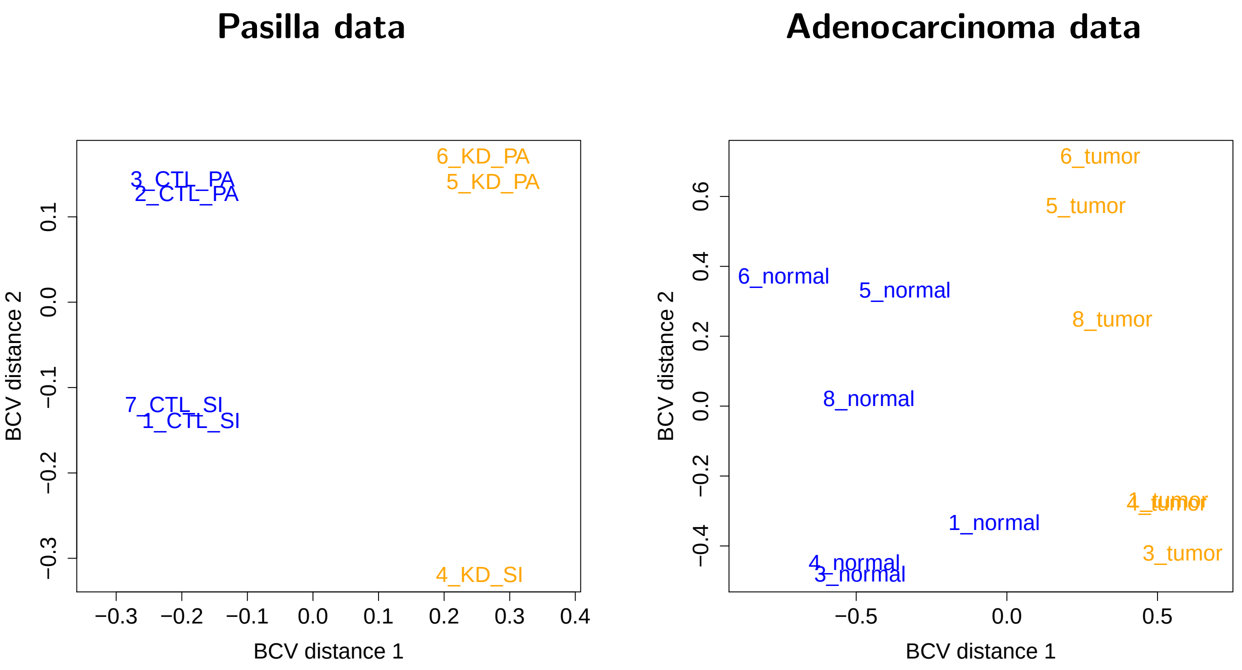

Figure S25: MDS plots based on top 500 most variable genes show the dissimilarity between samples from pasilla and adenocarcinoma studies. BVC - biological coefficient of variation. Sample labels are colored by condition. For the pasilla data, sample labels contain sample IDs, experimental condition (CTL - control, KD - knock-down of pasilla factor) and the type of sequencing (PA - paired-end, SI - single-end). There is a clear separation between control and knock-down samples, but also, paired-end and single-end samples create clusters that are very distinct within each condition, indicating that the library layout has a strong impact on the measured gene expression. For the adenocarcinoma data, sample labels correspond to the patient IDs and the tissue types that samples were extracted from. Samples group by condition. Nevertheless, there is a lot of heterogeneity among the individual patients - the range of the y-axis, that corresponds to the differences between the patients, is almost as wide as the range of x-axis, that represents the differences between the conditions. The order (from top to bottom) of patients in normal and tumor clusters is very similar. These two observations suggest that the differences between patients may be stronger than between the tissue type.

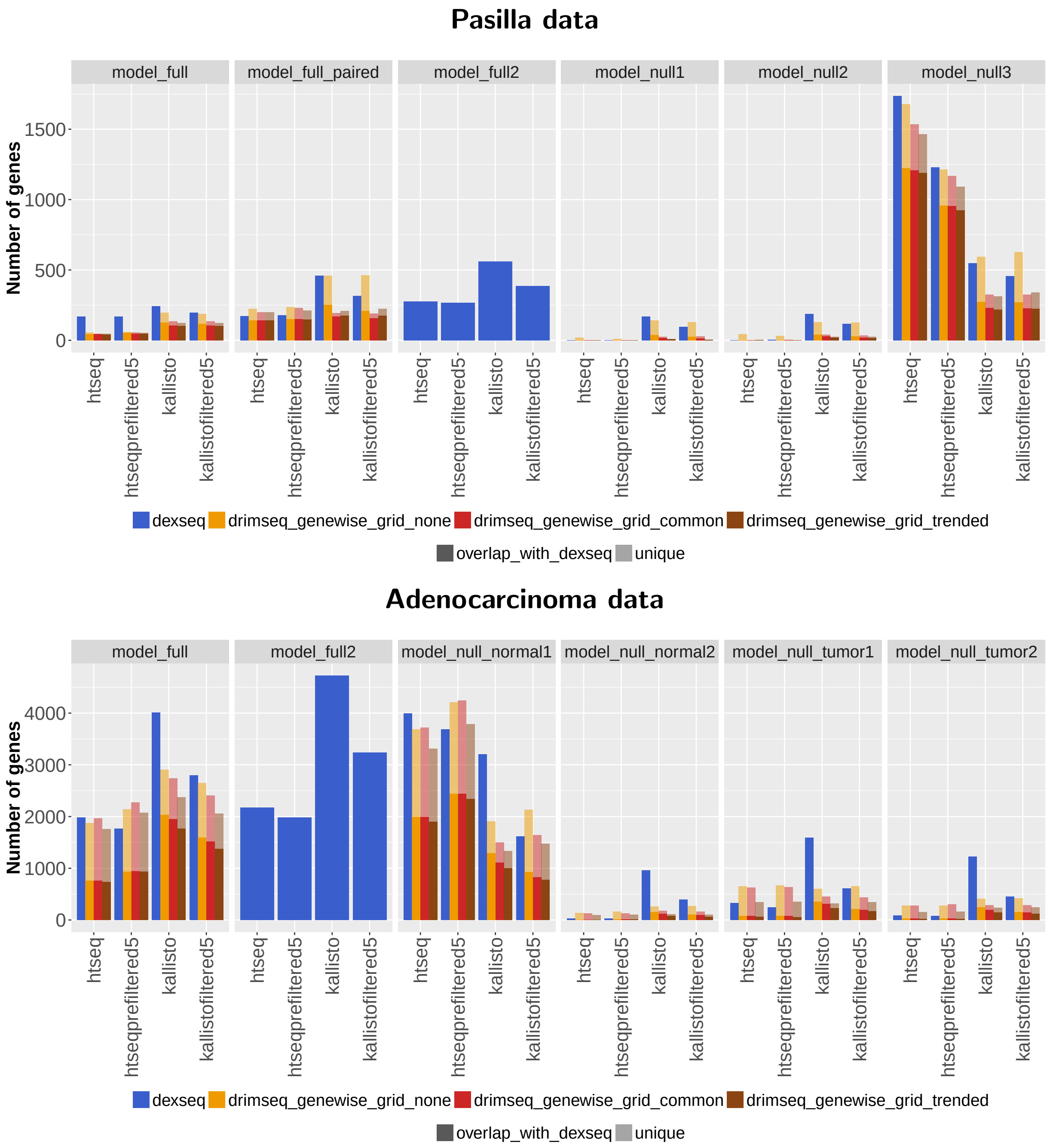

Figure S26: Number of genes detected as differentially spliced by *DEXSeq* and *DRIMSeq* with different dispersion methods (gene-wise estimates without moderation and with moderation to the common and to the trended dispersion) in pasilla and adenocarcinoma data. For the pasilla data, model full corresponds to the comparison of 4 control samples versus 3 knock-down. Model full paired is a comparison of 2 control versus 2 knock-down paired-end samples. Model full 2 is as model full but additionally accounts for the library layout. Null models compare different combinations of control samples (2 versus 2). For the adenocarcinoma data, full model corresponds to the two-group comparison of 6 control and 6 cancer samples. Model full 2 accounts for the fact that samples are paired. Null models are the two-group comparisons of different combinations of 3 versus 3 samples that come from the same condition: normal or tumor tissue. In the null comparisons, one expects to find no differential splicing since replicates from the same condition are compared. Currently, *DRIMSeq* does not support models with multiple covariates. The analyses were performed on *HTSeq* and *kallisto* counts estimated based on the full transcript catalog and the pre-filtered annotation where the lowly expressed transcripts were removed. Intensive colors specify the number of DS genes detected by *DRIMSeq* that overlap with *DEXSeq*. Transparent colors represent genes that are unique for *DRIMSeq*. FDR = 0.05.

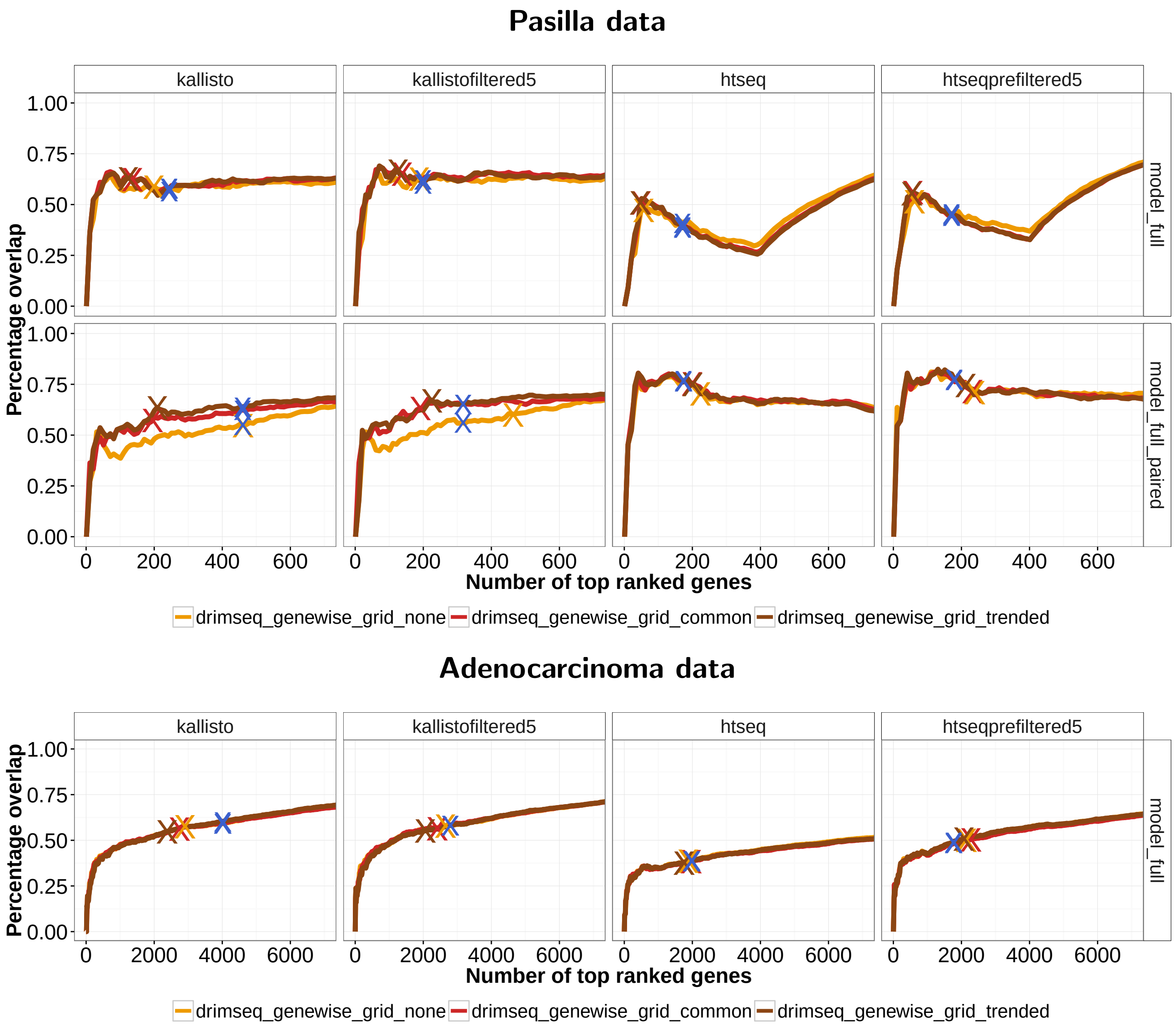

Figure S27: Concordance between *DEXSeq* and different versions of *DRIMSeq* for the top significant DS genes detected by each of the methods. "X" indicates the number of genes detected as DS for the FDR of 0.05. Blue "X" corresponds to *DEXSeq*.

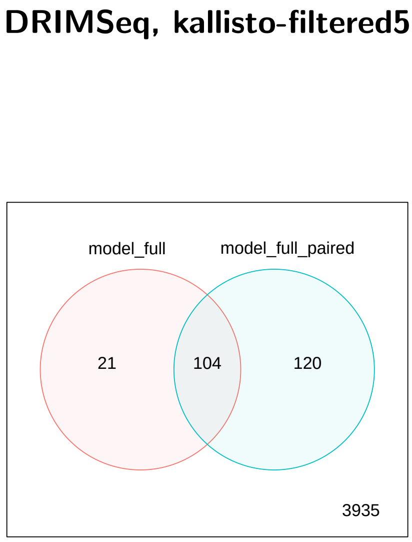

Figure S28: Pasilla data. Numbers of DS genes detected by *DRIMSeq* in the comparison of control versus knock-down based on all samples (model_full) or only from paired-end samples (model_full_paired). Results for *DRIMSeq* run with gene-wise dispersion moderated to the trend on *kallisto* filtered counts are shown. FDR = 0.05. When comparing the number of genes detected by *DRIMSeq* and *DEXSeq* (Figure S29) in models full and full_paired, their behavior is very similar. *DRIMSeq* and *DEXSeq* detect many more DS genes in the 2 versus 2 comparison than in the comparison based on all the data (with library layout as a batch effect), and quite a few of the DS genes detected in the full model are not discovered in the full_paired model.

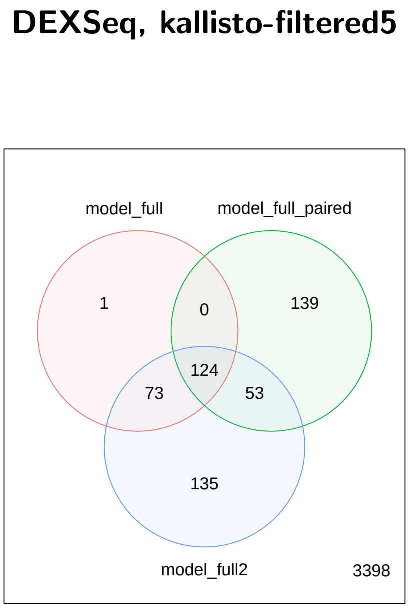

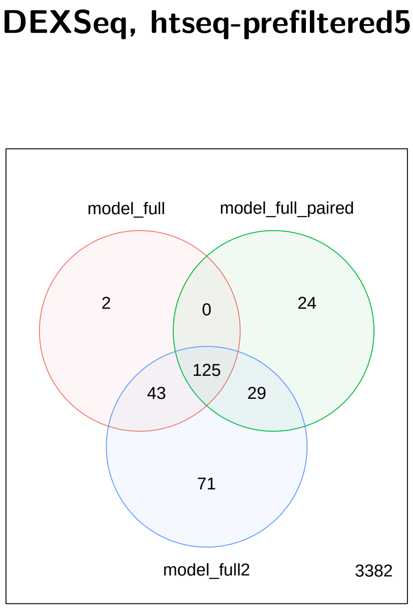

Figure S29: Pasilla data. Numbers of DS genes detected by *DEXSeq* in the comparison of control versus knock-down based on all samples with (model_full2) and without (model_full) accounting for the library layout or based on the paired-end samples only (model_full_paired). Results for *kallisto* filtered and *HTSeq* pre-filtered counts are shown. FDR = 0.05. When accounting for the library layout, *DEXSeq* detects almost all the DS genes from the full model analyses and a bunch of extra DS genes. This shows that library layout covariate, which is in fact a batch effect in this study, explains a substantial amount of variability in the data, and accounting for it should increase the power to detect DS genes.

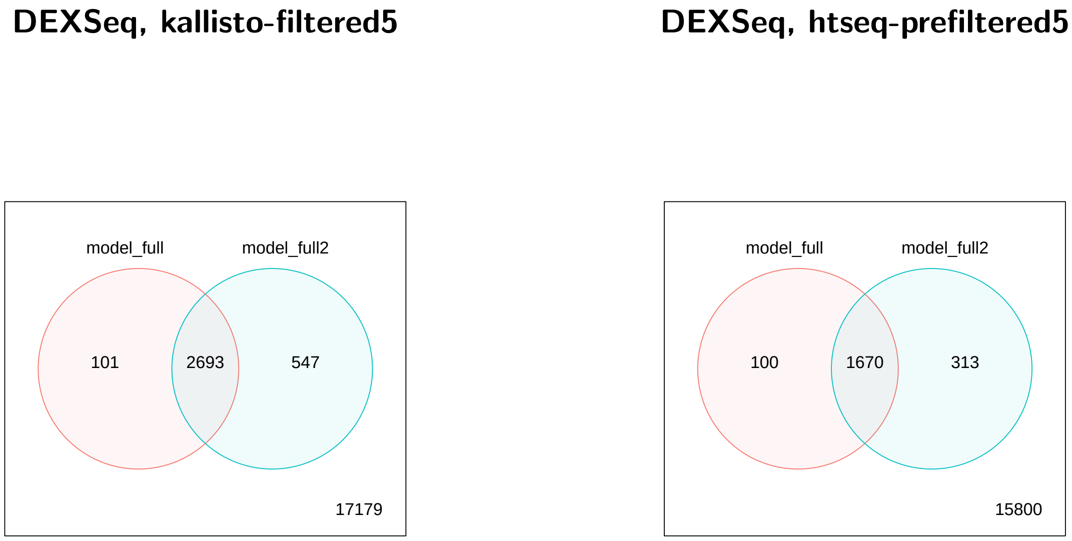

Figure S30: Adenocarcinoma data. Numbers of DS genes detected by *DEXSeq* in the comparison of normal versus tumor tissue with (model_full2) and without (model_full) accounting for the patient ID. Results for *kallisto* filtered and *HTSeq* pre-filtered counts are shown. FDR = 0.05. When accounting for the patient ID, *DEXSeq* detects a substantial amount of DS genes that overlap with the DS genes from the full model analyses and some extra DS genes. This shows that by accounting for the within-patient variability, the power to detect DS genes can be increased.

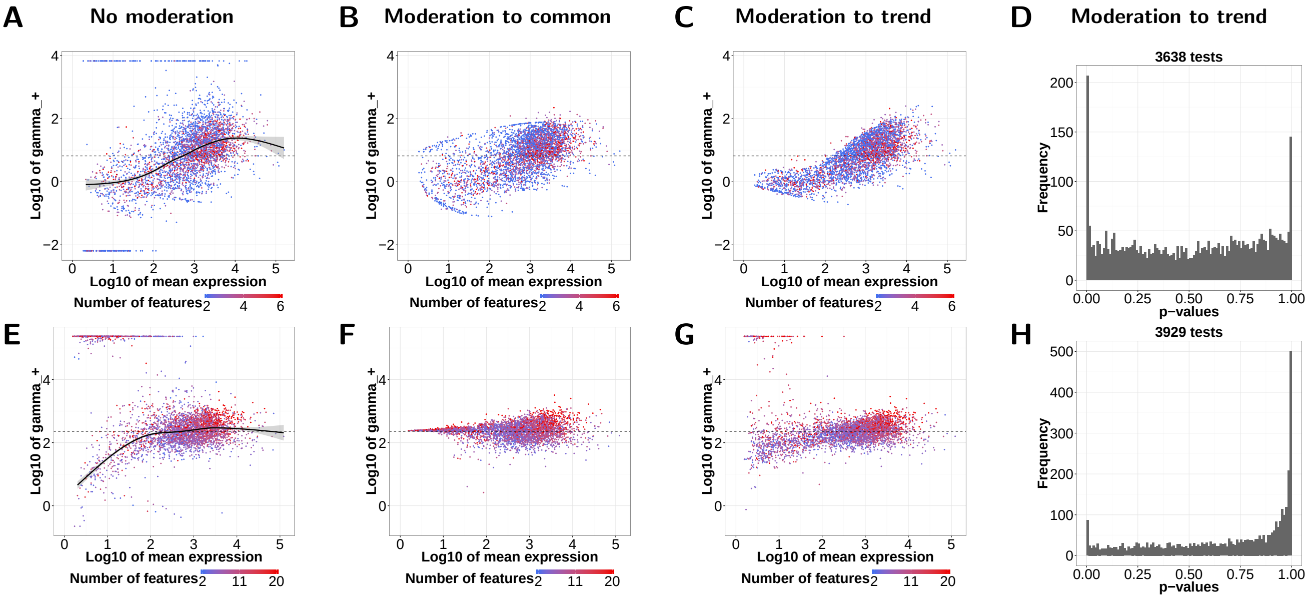

Figure S31: Results of the pasilla data analyses with *DRIMSeq*. Upper panels - *kallisto* counts, bottom panels - *HTSeq* counts. A, B, C, E, F, G: Concentration versus mean gene expression plots using different dispersion estimation approaches. Dashed line corresponds to the common dispersion estimate. Additionally, when no moderation is used, a smoothed curve is fitted. The fitting clearly indicates that there is a dispersion-mean trend present in the data. D, H: P-value histograms for trended dispersion used in the inference. For *kallisto* counts, the p-value distribution is more uniform with a sharp peak close to zero, which suggests a better fit of the DM model to transcript counts than to exonic counts.

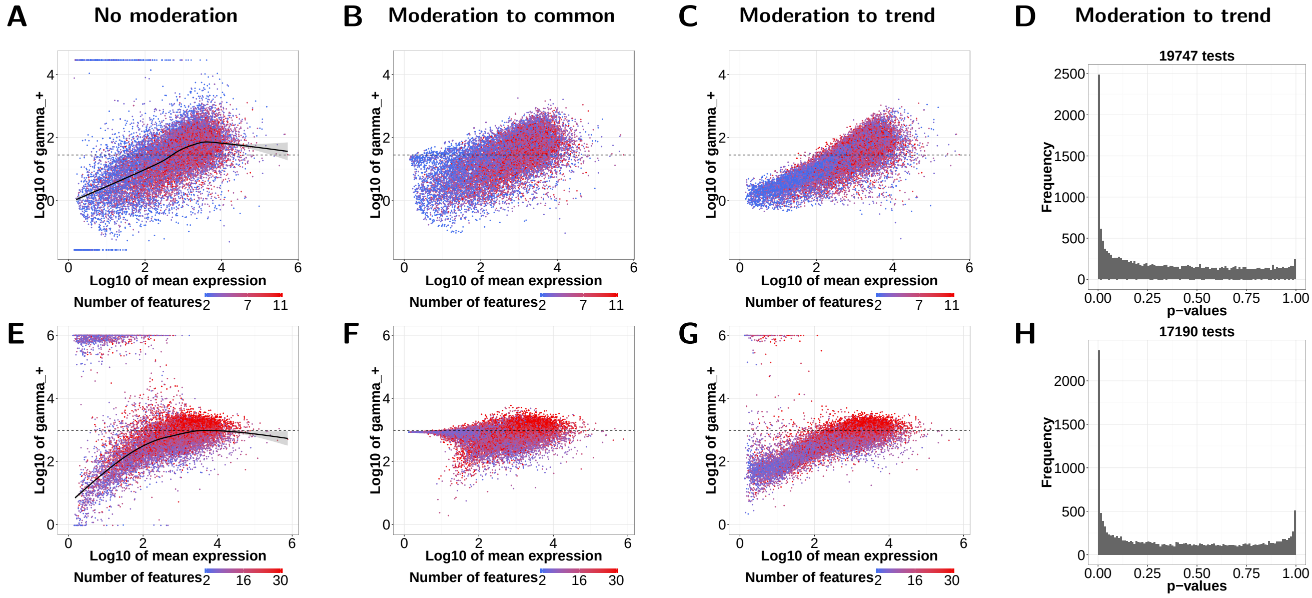

Figure S32: Results of the adenocarcinoma data analyses with *DRIMSeq*. Upper panels - *kallisto* counts, bottom panels - *HTSeq* counts. A, B, C, E, F, G: Concentration versus mean gene expression plots using different dispersion estimation approaches. Dashed line corresponds to the common dispersion estimate. Additionally, when no moderation is used, a smoothed curve is fitted. The fitting clearly indicates that there is a dispersion-mean trend present in the data. D, H: P-value histograms for trended dispersion used in the inference. For *kallisto* counts, the p-value distribution is more uniform with a sharp peak close to zero, which suggests a better fit of the DM model to transcript counts than to exonic counts.

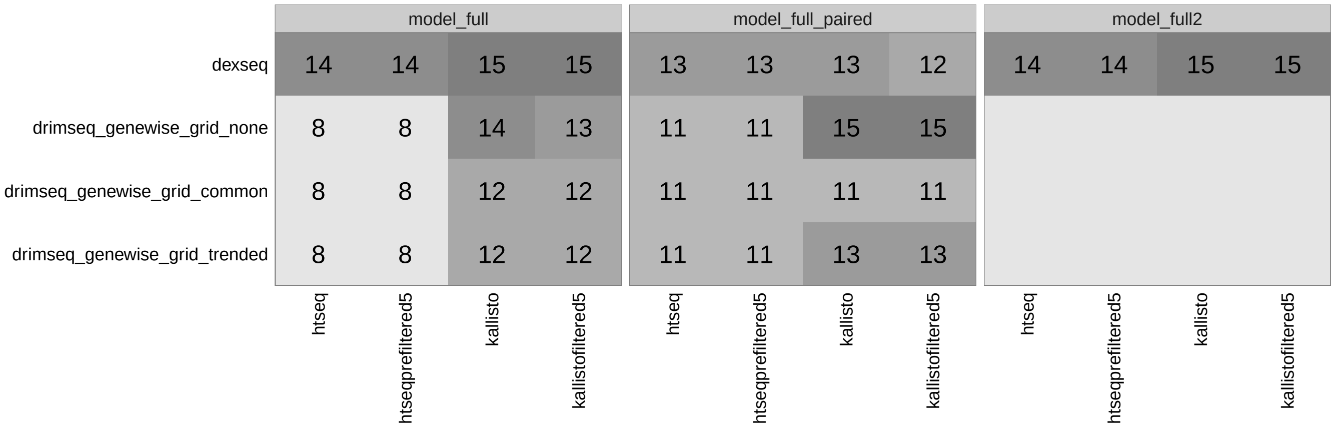

Figure S33: Pasilla data. Number of genes that were validated by Brooks et al. for the alternative exon usage and detected as DS by *DEXSeq* and *DRIMSeq* (FDR $= 0.05$). Model full - comparison of 4 control samples versus 3 knock-down. Model full paired - comparison of 2 versus 2 paired-end samples. Model full 2 - as model full but additionally accounts for the library layout.

	htseq				htseqprefiltered5			
	dexseq	drimseq_genewise_grid_none	drimseq_genewise_grid_common	drimseq_genewise_grid_trended	dexseq	drimseq_genewise_grid_none	drimseq_genewise_grid_common	drimseq_genewise_grid_trended
trol_FBgn0261451	0.00e+00	NA	NA	NA	0.00e+00	NA	NA	NA
slik_FBgn0035001	0.00e+00	1.86e-09	8.85e-09	7.83e-09	0.00e+00	1.86e-09	1.04e-08	7.43e-09
sesB_FBgn0003360	0.00e+00	2.12e-02	1.03e-02	8.93e-03	0.00e+00	9.17e-03	3.04e-03	3.29e-03
sba_FBgn0016754	3.69e-05	1.53e-03	6.29e-03	1.02e-02	5.98e-05	1.50e-03	6.45e-03	9.59e-03
RhoGAP19D_FBgn0031118	2.38e-08	1.00e+00	1.00e+00	1.00e+00	1.96e-08	1.00e+00	1.00e+00	1.00e+00
PhKgamma_FBgn0011754	4.84e-07	2.51e-01	3.40e-01	3.94e-01	5.99e-07	1.36e-01	1.64e-01	1.94e-01
osa_FBgn0261885	1.06e-07	4.27e-07	1.77e-06	1.70e-06	8.07e-08	1.55e-07	1.26e-06	7.60e-07
msn_FBgn0010909	0.00e+00	6.90e-01	9.56e-01	9.68e-01	0.00e+00	3.97e-01	5.06e-01	4.83e-01
LanB1_FBgn0261800	3.79e-02	1.00e+00	1.00e+00	1.00e+00	2.71e-02	1.00e+00	1.00e+00	1.00e+00
dre4_FBgn0002183	2.80e-05	1.00e+00	1.00e+00	1.00e+00	4.90e-05	1.00e+00	1.00e+00	1.00e+00
CG8920_FBgn0027529	2.26e-05	3.55e-05	4.17e-04	1.14e-03	1.41e-05	7.37e-04	4.80e-03	8.30e-03
CG7337_FBgn0031374	1.95e-11	4.02e-06	8.46e-06	1.29e-05	3.49e-11	5.43e-07	1.69e-06	2.29e-06
CG4829_FBgn0030796	0.00e+00	2.09e-02	2.60e-02	2.40e-02	0.00e+00	2.62e-03	3.26e-03	3.07e-03
CG34439_FBgn0085468	1.00e+00	1.00e+00	1.00e+00	1.00e+00	1.00e+00	1.00e+00	1.00e+00	1.00e+00
cg_FBgn0000289	1.00e+00	1.00e+00	1.00e+00	1.00e+00	1.00e+00	1.00e+00	1.00e+00	1.00e+00
bmm_FBgn0036449	0.00e+00	7.66e-08	1.74e-07	2.28e-07	0.00e+00	3.22e-08	8.73e-08	8.45e-08
Ant2_FBgn0025111	1.00e+00	1.00e+00	1.00e+00	1.00e+00	1.00e+00	1.00e+00	1.00e+00	1.00e+00

	kallisto				kallistofiltered5			
	dexseq	drimseq_genewise_grid_none	drimseq_genewise_grid_common	drimseq_genewise_grid_trended	dexseq	drimseq_genewise_grid_none	drimseq_genewise_grid_common	drimseq_genewise_grid_trended
trol_FBgn0261451	0.00e+00	9.99e-35	1.50e-33	1.20e-34	0.00e+00	2.13e-27	2.13e-26	2.57e-27
slik_FBgn0035001	4.99e-14	4.20e-02	7.07e-02	5.79e-02	4.60e-14	6.24e-02	9.89e-02	8.52e-02
sesB_FBgn0003360	0.00e+00	1.95e-137	8.62e-87	4.66e-93	0.00e+00	8.93e-138	6.68e-84	3.20e-97
sba_FBgn0016754	0.00e+00	4.31e-04	6.39e-04	5.72e-04	0.00e+00	5.97e-04	9.06e-04	8.12e-04
RhoGAP19D_FBgn0031118	7.21e-03	2.01e-07	6.26e-07	1.75e-07	5.46e-03	1.65e-07	5.81e-07	1.77e-07
PhKgamma_FBgn0011754	1.81e-05	1.25e-10	1.80e-09	5.52e-09	1.17e-05	1.01e-10	2.07e-09	5.75e-09
osa_FBgn0261885	0.00e+00	1.28e-27	2.38e-24	5.95e-27	0.00e+00	1.01e-27	3.63e-24	5.28e-27
msn_FBgn0010909	5.35e-14	2.64e-02	3.65e-02	3.59e-02	0.00e+00	2.22e-02	2.90e-02	3.04e-02
LanB1_FBgn0261800	3.97e-01	8.39e-02	2.78e-01	1.45e-01	4.29e-01	7.99e-02	2.83e-01	1.26e-01
dre4_FBgn0002183	3.78e-02	6.69e-04	5.62e-02	1.24e-02	3.81e-02	5.70e-04	6.50e-02	9.13e-03
CG8920_FBgn0027529	3.18e-02	4.77e-06	3.50e-04	9.60e-04	3.13e-02	3.88e-06	3.77e-04	9.66e-04
CG7337_FBgn0031374	0.00e+00	7.75e-08	2.84e-06	3.34e-06	2.06e-12	5.47e-09	1.56e-06	1.28e-06
CG4829_FBgn0030796	2.64e-10	8.82e-32	1.86e-21	5.15e-29	1.30e-10	1.22e-38	3.05e-25	7.42e-35
CG34439_FBgn0085468	1.54e-02	2.39e-03	1.29e-02	5.30e-02	1.64e-02	2.09e-03	1.32e-02	5.79e-02
cg_FBgn0000289	1.86e-02	3.77e-01	5.04e-01	5.06e-01	2.23e-02	3.49e-01	4.93e-01	4.86e-01
bmm_FBgn0036449	4.36e-12	2.73e-119	1.98e-51	5.49e-55	1.43e-12	1.66e-119	1.66e-48	1.84e-56
Ant2_FBgn0025111	3.91e-01	5.70e-01	6.67e-01	6.93e-01	4.63e-01	5.49e-01	6.50e-01	6.60e-01

Figure S34: Pasilla data. Table with adjusted p-values obtained by *DEXSeq* and *DRIMSeq* in the full model analyses (comparison of 4 control samples versus 3 knock-down) for the genes that were validated by Brooks et al. for the alternative exon usage.

	htseq				htseqprefiltered5				kallisto				kallistofiltered5			
	dexseq	drimseq_genewise_grid_none	drimseq_genewise_grid_common	drimseq_genewise_grid_trended	dexseq	drimseq_genewise_grid_none	drimseq_genewise_grid_common	drimseq_genewise_grid_trended	dexseq	drimseq_genewise_grid_none	drimseq_genewise_grid_common	drimseq_genewise_grid_trended	dexseq	drimseq_genewise_grid_none	drimseq_genewise_grid_common	drimseq_genewise_grid_trended
trol_FBgn0261451	0.00e+00	NA	NA	NA	0.00e+00	NA	NA	NA	0.00e+00	6.47e−193	6.95e−152	1.01e−189	0.00e+00	2.70e−189	5.88e−126	3.51e−183
slik_FBgn0035001	0.00e+00	9.44e−58	1.98e−56	5.28e−50	0.00e+00	2.90e−55	1.16e−53	3.68e−50	3.32e−07	3.21e−11	4.92e−08	8.58e−11	2.06e−05	9.47e−10	1.69e−06	1.98e−09
sesB_FBgn0003360	0.00e+00	8.37e−52	4.01e−47	1.95e−44	0.00e+00	6.35e−52	1.59e−46	6.63e−44	0.00e+00	4.57e−35	8.59e−22	4.28e−32	0.00e+00	4.31e−35	9.90e−20	4.37e−32
sba_FBgn0016754	2.33e−02	6.74e−04	3.40e−03	3.30e−03	1.39e−02	2.87e−04	1.35e−03	2.55e−03	5.96e−10	9.11e−09	1.24e−07	1.13e−07	2.89e−09	8.55e−08	1.34e−06	9.32e−07
RhoGAP19D_FBgn0031118	0.00e+00	6.51e−23	2.02e−22	6.20e−23	0.00e+00	2.03e−23	1.31e−22	2.03e−23	2.21e−05	2.25e−23	3.05e−13	6.68e−21	1.53e−05	2.40e−23	6.84e−12	1.89e−21
PhKgamma_FBgn0011754	2.52e−05	6.48e−05	3.74e−04	3.49e−04	1.51e−05	2.85e−05	1.86e−04	2.51e−04	9.66e−04	2.04e−04	1.09e−03	1.46e−03	1.72e−03	1.66e−04	1.12e−03	1.23e−03
osa_FBgn0261885	0.00e+00	1.61e−32	6.88e−32	1.35e−28	0.00e+00	2.17e−33	1.41e−32	3.26e−30	0.00e+00	4.85e−79	1.27e−43	5.03e−75	0.00e+00	1.46e−79	3.39e−38	3.04e−73
msn_FBgn0010909	0.00e+00	1.06e−49	2.50e−49	2.70e−47	0.00e+00	1.21e−51	3.67e−51	3.84e−50	0.00e+00	1.12e−75	4.15e−36	3.78e−67	0.00e+00	1.17e−89	5.33e−35	9.73e−72
LanB1_FBgn0261800	5.11e−03	1.42e−01	1.66e−01	2.31e−01	2.57e−03	7.65e−02	8.58e−02	8.46e−02	9.51e−02	7.40e−10	5.95e−01	2.18e−03	3.29e−01	6.13e−10	6.61e−01	1.04e−02
dre4_FBgn0002183	5.41e−04	5.95e−01	6.31e−01	6.72e−01	2.58e−04	3.98e−01	4.14e−01	3.79e−01	2.20e−02	3.17e−07	1.35e−01	1.83e−04	5.64e−02	2.66e−07	2.09e−01	5.16e−04
CG8920_FBgn0027529	3.64e−01	1.32e−02	2.72e−02	1.46e−02	2.57e−01	2.00e−02	4.44e−02	2.71e−02	1.36e−01	9.76e−04	8.11e−02	6.68e−02	2.78e−01	8.29e−04	1.23e−01	7.82e−02
CG7337_FBgn0031374	3.49e−06	1.17e−11	2.37e−11	5.01e−11	2.59e−06	1.46e−13	5.91e−13	1.21e−12	2.07e−05	2.24e−16	7.07e−09	1.08e−09	2.70e−03	9.70e−11	4.49e−04	5.08e−05
CG4829_FBgn0030796	0.00e+00	1.91e−12	6.48e−13	1.19e−15	0.00e+00	1.46e−12	1.38e−13	9.70e−18	0.00e+00	1.62e−17	7.45e−11	7.92e−21	0.00e+00	1.20e−17	3.28e−10	1.29e−22
CG34439_FBgn0085468	1.00e+00	1.00e+00	1.00e+00	1.00e+00	1.00e+00	9.69e−01	9.52e−01	9.75e−01	6.82e−02	2.51e−03	7.28e−02	3.06e−01	1.28e−01	2.20e−03	9.37e−02	3.13e−01
cg_FBgn0000289	2.91e−01	1.97e−01	2.31e−01	2.76e−01	1.59e−01	1.04e−01	1.21e−01	1.41e−01	1.07e−03	5.87e−02	3.81e−01	2.10e−01	3.17e−02	5.49e−02	4.47e−01	2.20e−01
bmm_FBgn0036449	0.00e+00	6.16e−21	3.98e−21	1.28e−20	0.00e+00	3.18e−21	5.03e−22	6.13e−21	3.20e−03	5.21e−09	3.15e−09	1.15e−16	1.95e−03	4.29e−09	2.39e−09	3.74e−18
Ant2_FBgn0025111	1.00e+00	1.00e+00	1.00e+00	1.00e+00	1.00e+00	9.55e−01	9.43e−01	9.70e−01	2.06e−01	2.82e−01	3.43e−01	3.28e−01	2.68e−01	2.62e−01	3.28e−01	2.97e−01

Figure S35: Pasilla data. Table with adjusted p-values obtained by *DEXSeq* and *DRIMSeq* in the model full paired analyses (comparison of 2 versus 2 paired-end samples) for the genes that were validated by Brooks et al. for the alternative exon usage.

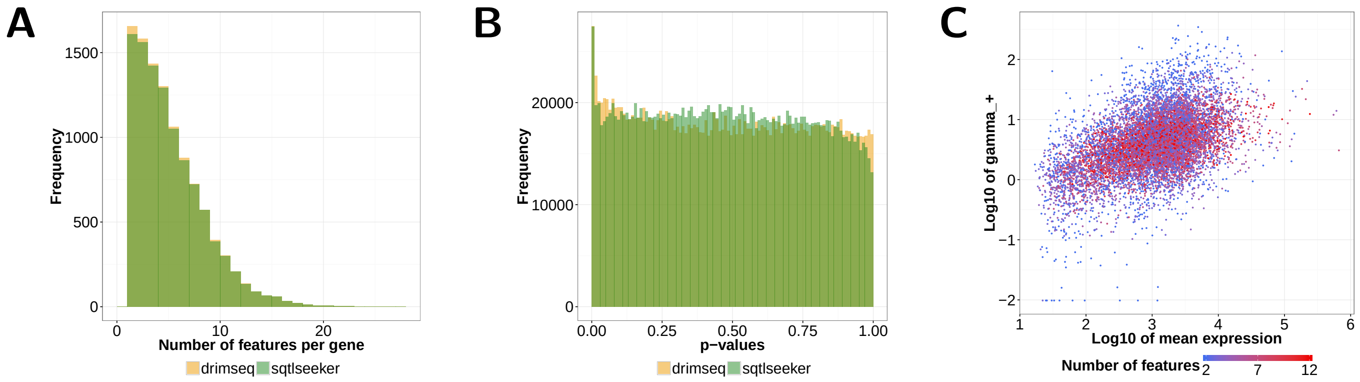

Figure S36: tuQTL analyses of the CEU population from GEUVADIS data. A: Distribution of the number of transcripts per gene for genes that had a non NA p-value assigned by *DRIMSeq* or *sQTLseekeR*. They should be almost identical since the count data is identical for both of the pipelines. B: Distributions of p-values obtained by *DRIMSeq* and *sQTLseekeR*. C: Concentration versus mean gene expression plot from the *DRIMSeq* analysis with gene-wise dispersion without any moderation. Notice that none of the estimated values lies on the upper grid boundary, indicating that the concentration estimates are more stable in the analyses based on 91 samples.

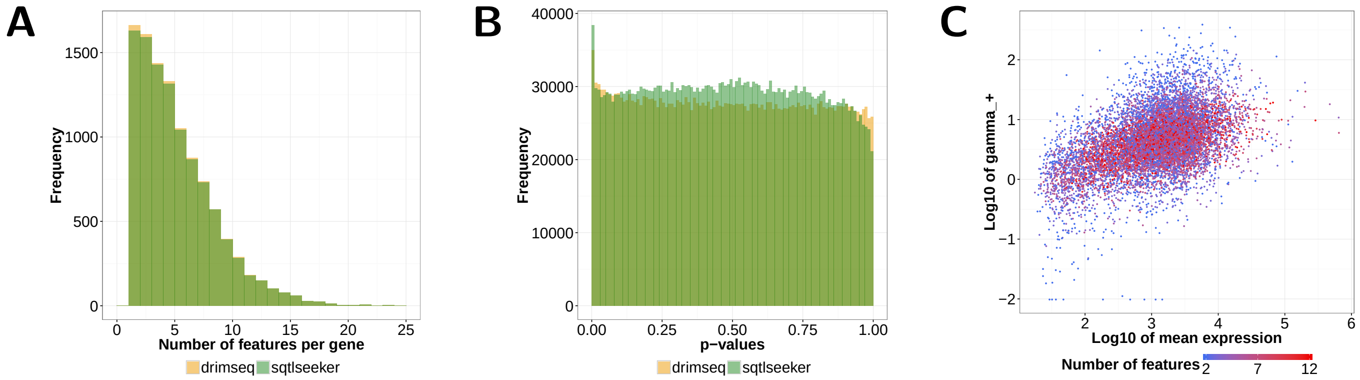

Figure S37: tuQTL analyses of the YRI population from GEUVADIS data. A: Distribution of the number of transcripts per gene for genes that had a non NA p-value assigned by *DRIMSeq* or *sQTLseekeR*. They should be almost identical since the count data is identical for both of the pipelines. B: Distributions of p-values obtained by *DRIMSeq* and *sQTLseekeR*. C: Concentration versus mean gene expression plot from the *DRIMSeq* analysis with gene-wise dispersion without any moderation. Notice that none of the estimated values lies on the upper grid boundary, indicating that the concentration estimates are more stable in the analyses based on 89 samples.

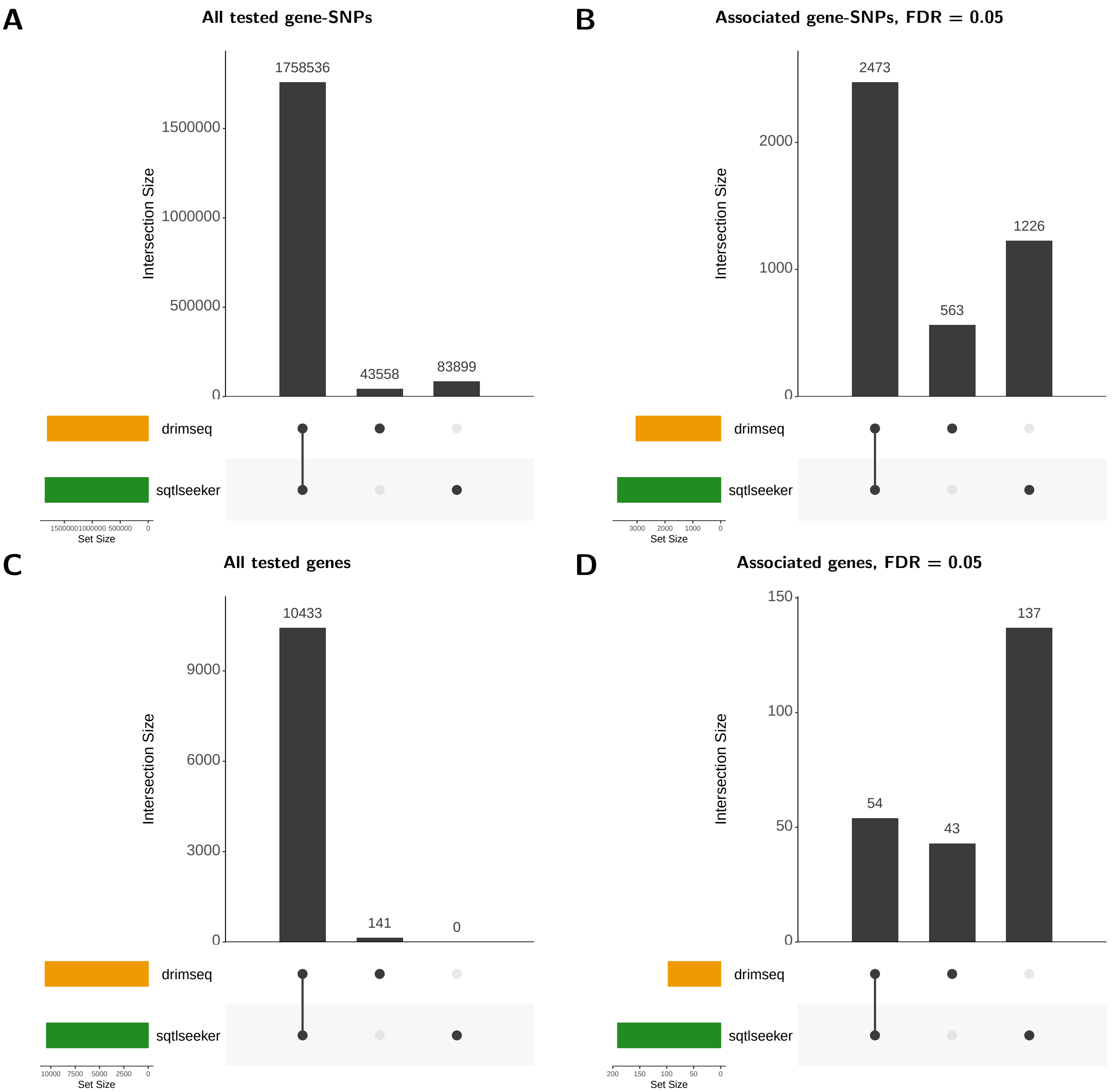

Figure S38: tuQTL analyses of the CEU population from GEUVADIS data. A, C: Numbers of gene-SNP pairs and genes that were tested by *DRIMSeq* and *sQTLseekeR*. B, D: Numbers of significant tuQTLs and associated genes (FDR = 0.05). *sQTLseekeR* detects more tuQTLs than *DRIMSeq*, and substantially more genes that are associated to its unique variants.

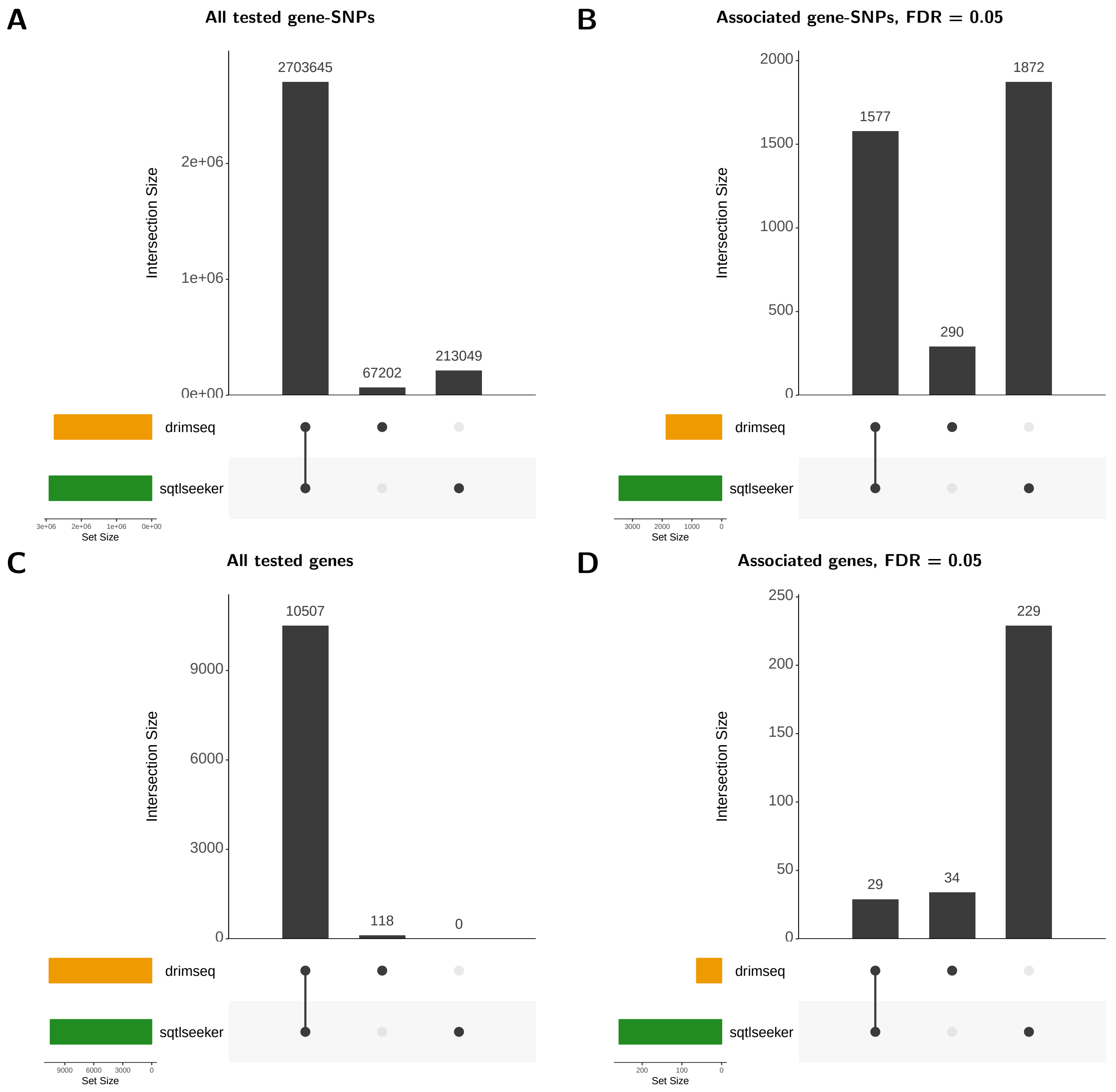

Figure S39: tuQTL analyses of the YRI population from GEUVADIS data. A, C: Numbers of gene-SNP pairs and genes that were tested by *DRIMSeq* and *sQTLseekeR*. B, D: Numbers of significant tuQTLs and associated genes (FDR = 0.05). *sQTLseekeR* detects more tuQTLs than *DRIMSeq*, and substantially more genes that are associated to its unique variants.

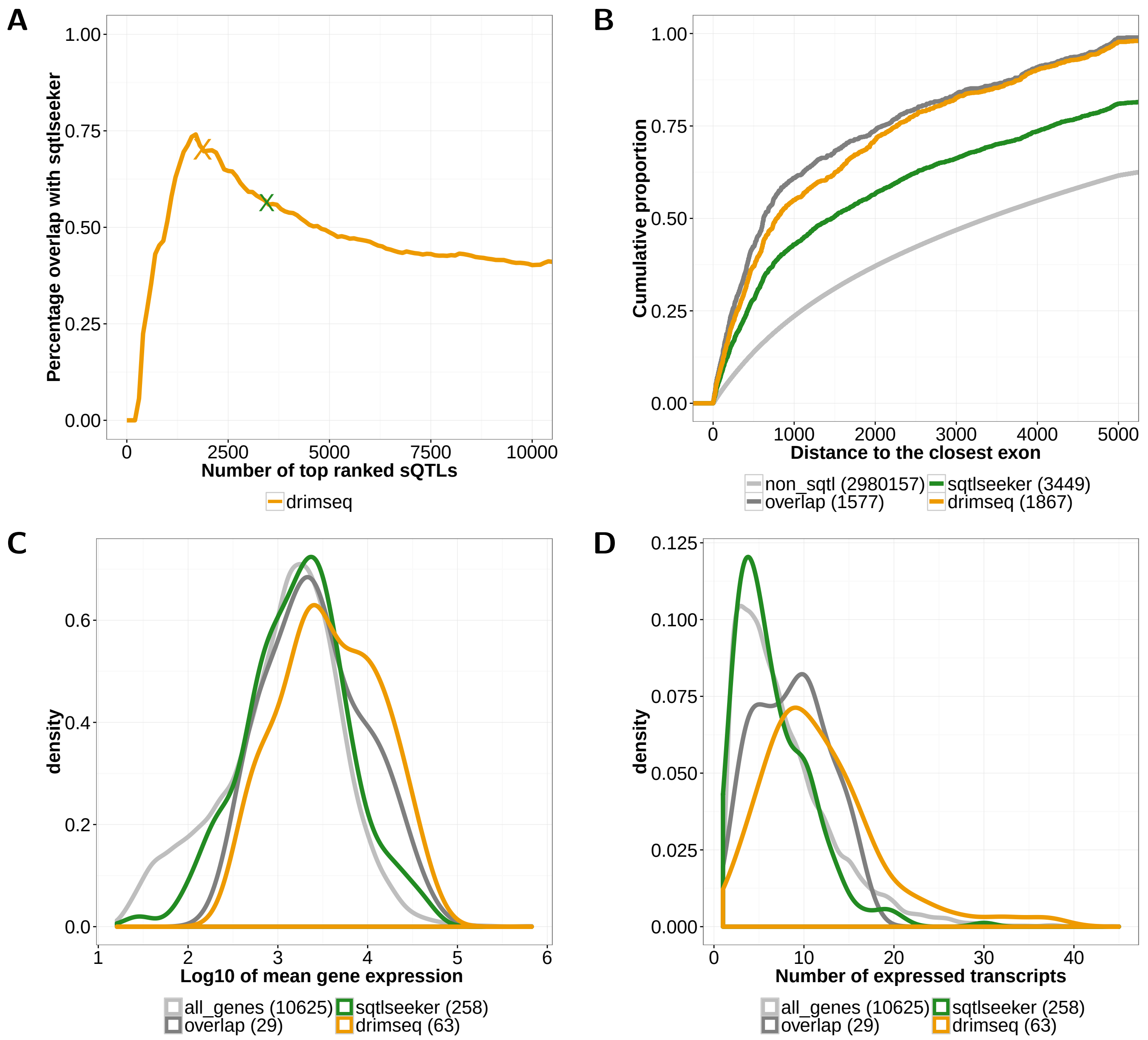

Figure S40: tuQTL analyses of the YRI population from GEUVADIS data. A: Concordance between *sQTLseekeR* and *DRIMSeq*. "X" indicates number of tuQTLs for FDR = 0.05. Panel B, C and D show characteristics of tuQTLs and genes detected by *sQTLseekeR* or *DRIMSeq* for FDR = 0.05. Values in the brackets indicate numbers of tuQTLs or genes in a given set. Dark gray line corresponds to tuQTLs or genes that were identified by both of the methods (overlap). B: Distance to the closest exon of intronic tuQTLs. Light gray line (non_sqtl) corresponds to intronic tuQTLs that were not called by any of the methods. C: Distribution of mean gene expression for genes that are associated with tuQTLs. D: Distribution of the number of expressed transcripts for genes that are associated with tuQTLs. Light gray lines (all_genes) represent corresponding features for all the analyzed genes.

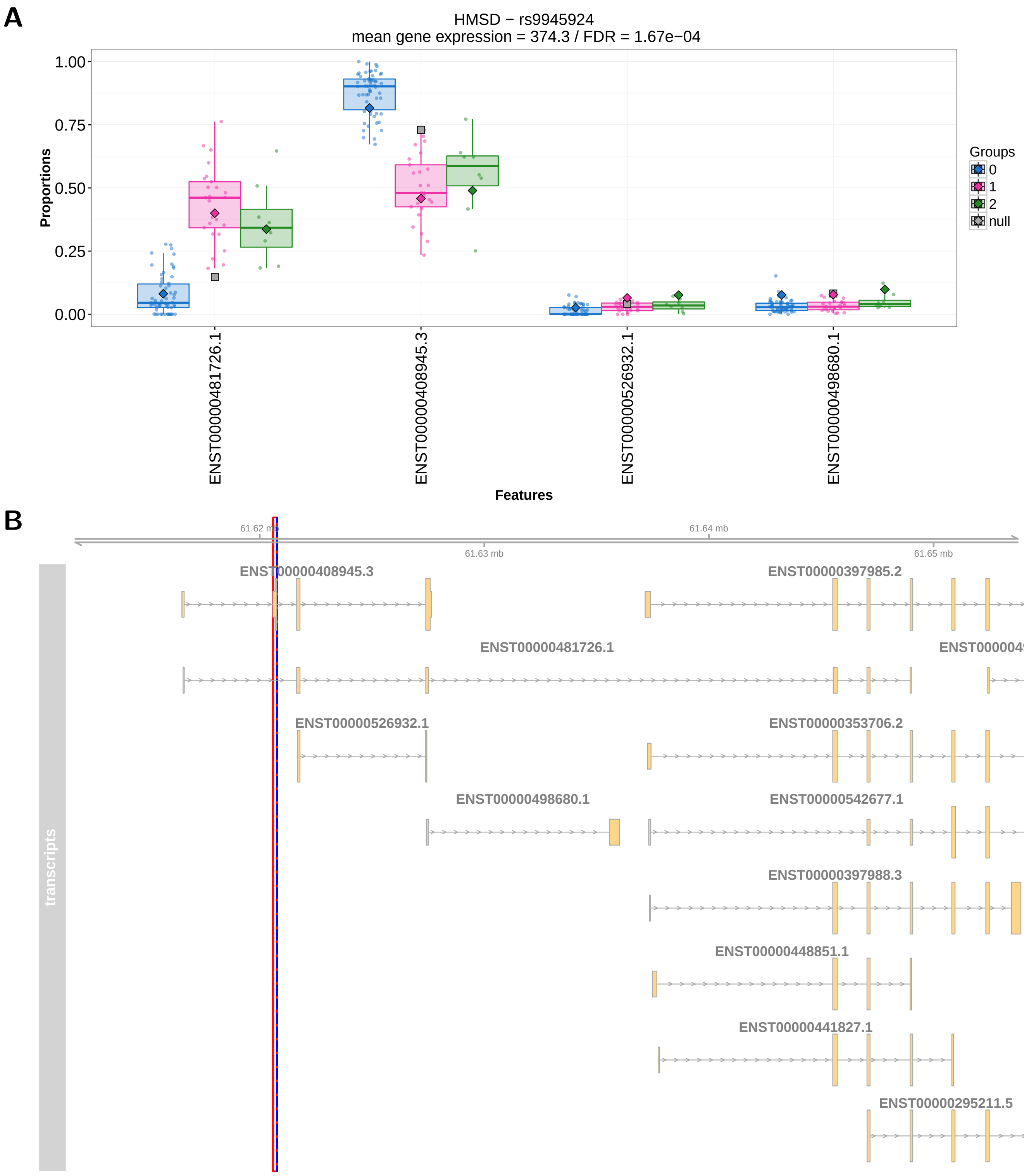

Figure S41: tuQTL analyses of the CEU population from GEUVADIS data. An example of an tuQTL validated with PCR in *GLiMMPS* and detected by *DRIMSeq* (FDR = 0.05). A: Box-plots represent the distributions of transcript proportions given for each genotype (0, 1 or 2 alleles different from the reference). Diamonds represent the DM estimated transcript proportions in each of the genotype groups (full model estimates), gray squares indicate the proportions estimated from the polled data (null model). B: Gene structure of HMSD with red area indicating the exon that was used for the validation and blue dashed line specifying the location of rs9945924 SNP.

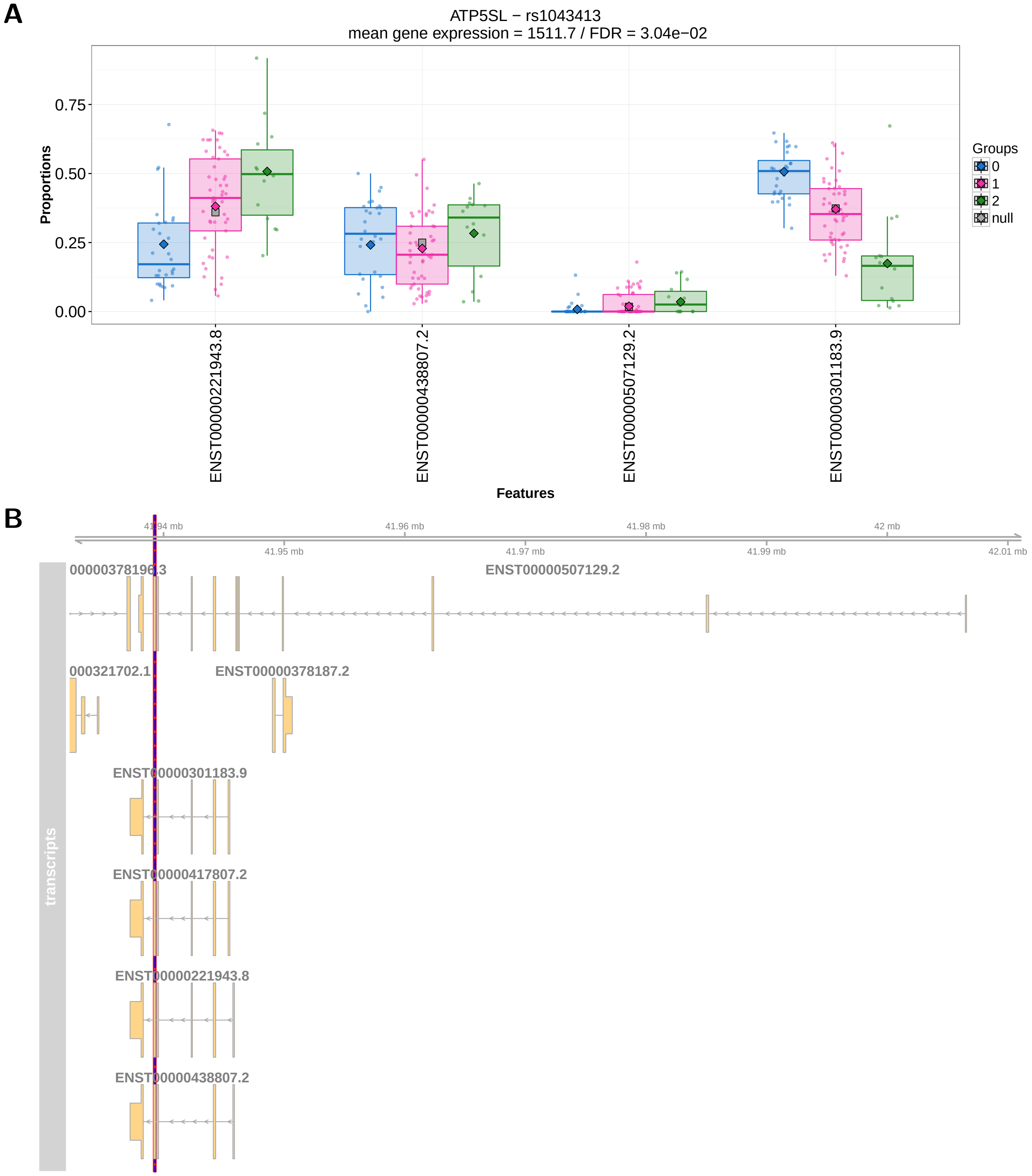

Figure S42: tuQTL analyses of the CEU population from GEUVADIS data. An example of an tuQTL validated with PCR in *GLiMMPS* and detected by *DRIMSeq* (FDR = 0.05). A: Box-plots represent the distributions of transcript proportions given for each genotype (0, 1 or 2 alleles different from the reference). Diamonds represent the DM estimated transcript proportions in each of the genotype groups (full model estimates), gray squares indicate the proportions estimated from the polled data (null model). B: Gene structure of ATP5SL with red area indicating the exon that was used for the validation and blue dashed line specifying the location of rs1043413 SNP. The rs1043413 SNP is in high linkage disequilibrium [7] with GWAS SNP rs17318596 associated with height [9].

Supplementary Tables

Table S1: Overview of DS methods. The updated list can be found under the link `http://rpubs.com/gosianow/DS_methods`.

Application	DS method	Counting method	Type of DS analysis	Modeling	Description of the DS method	Extra notes/features
DS	DEXSeq [10]	DEXSeq/ HTSeq [10, 11]	Local; exon usage	At the gene level but exons are treated as independent	Fits, per gene, a generalized linear model with bin-condition interactions assuming the negative-binomial distribution for counts; Each exon-condition interaction is fitted within a separate model containing interaction coefficient for only this exon; Fits sample effects to handle the gene expression variability; Involves sharing information between genes, for the dispersion estimation	Allows analyses with multiple covariates; Returns exon-level p-values, but has a module to transform these p-values into gene-level adjusted p-values
sQTL	Altrans/ FastQTL [12, 13]	Altrans [12]	Local; exon link (junction) usage	Modeling each exon link separately	For the exon-link quantifications, it uses the paired-end reads with mates mapping to different exons and junction reads, and account for the insert size; Inference based on the F value which is a ratio between the coverage of a link and the sum of coverages of all the links that the primary exon makes; Employs Pearson correlation, from FastQTL [13], as a measure of association between genotype and Gaussian transformed exon link ratios	Applies a permutation approach to estimate p-values
DS	rMATS [14]	rMATS	Local; splicing events	Modeling each splicing event separately	For the splicing event quantification, it uses the inclusion reads and exclusion reads (no reads that map to the body of constitutive exons); In the DS inference, uses a hierarchical model: binomial distribution for the inclusion read counts given the inclusion level PSI to model the estimation uncertainty of PSI in individual replicate and logit-normal mixed model for PSI with random effects for each sample to account for the variability among replicates within conditions	Allows unpaired and paired comparisons
sQTL	GLiMMPS [7]	GLiMMPS	Local; splicing events	Modeling each splicing event separately	For the splicing event quantification, it uses the inclusion reads and exclusion reads (no reads that map to the body of constitutive exons); Uses a GLMM with a hierarchical model: binomial distribution for the inclusion read counts given the inclusion level PSI to model the estimation uncertainty of PSI in individual replicate and logit-normal mixed model for PSI with fixed effects for the genotype and random effects for each sample to account for the variability within the same genotype group;	Computation done with glmer function from R package lme4; Using permutations for the FDR estimation
sQTL	Jia et al. [15]	PennSeq [16]	Local; splicing events	Modeling each splicing event separately	For the splicing event quantification, PennSeq uses all reads mapped to a given exon-trio, i.e., the inclusion reads and exclusion reads and reads that map to the body of the constitutive exons, and it accounts for the paired-end nature of the data; Applies a random-effects meta regression using logit-normal for inclusion levels PSI with fixed effects for the genotype, random effects for each sample and a component representing the PSI estimation uncertainty from PennSeq;	Computation is performed with metafor R package; Jia et al. considered also a beta regression and GLMM like in GliMMPS, but REMR was performing the best
DS	MISO [17]	MISO	Local; splicing events; Global; transcript usage	Modeling each splicing event separately in exon-centric analyses; Multivariate for isoform-centric analyses	For the quantification of exon or isoform inclusion levels, it employs a Bayesian inference which uses the inclusion reads and exclusion reads and reads that map to the body of the constitutive exons and captures the information about library inserts in paired-end data; The inference about DS between two samples is based on the posterior probabilities of isoform inclusions (or exon-inclusions) which are compared using the Bayes factor	DS analysis only for 1 vs. 1 comparisons; Performs exon-centric and isoform-centric DS analyses

Table S1 Continued: Overview of DS methods. The updated list can be found under the link `http://rpubs.com/gosianow/DS_methods`.

Application	DS method	Counting method	Type of DS analysis	Modeling	Description of the DS method	Extra notes/features
DS	Cuffdiff2 [18]	Cufflinks [19]	Global; transcript usage	Multivariate	Uses the Jensen-Shannon divergence metric on probability distributions of isoform proportions (obtained from Cufflinks) as a measure of changes in isoform relative abundances between samples; The test statistic is the square root of the Jensen-Shannon divergence divided by its standard error (one-sided t -test)	DS based on transcripts grouped by TSS or promoter usage
sQTL	sQTLseekeR [20]		Global; transcript usage	Multivariate	To account for the gene expression, it transforms transcript quantifications into ratios; To test for the association between a genotype and transcript ratios, it uses a test proposed by Anderson which is similar to a multivariate analysis of variance (MANOVA) without assuming any probabilistic distribution. The difference between the within-group and between-group variability is measured by a pseudo-F ratio score; The Hellinger distance in used as a dissimilarity measure between transcript ratios	Employs permutations to estimate p-values; Allows comparison between multiple groups of samples
DS	LeafCutter [21]	LeafCutter	Local; intron usage	Multivariate	For the quantification of intron excision, uses only the junction reads; For the DS inference, uses the Dirichlet-multinomial generalized linear model	Allows analyses with multiple covariates; Annotation free
sQTL	LeafCutter/ FastQTL [21, 13]	LeafCutter [21]	Local; intron usage	Modeling each intron separately	For the quantification of intron excision, uses only the junction reads; Inference based on the proportions of reads supporting excised introns; Employs Pearson correlation, from FastQTL [13], as a measure of association between genotype and intron excision proportions	Applies a permutation approach to estimate p-values; Annotation free
DS and sQTL	DRIMSeq		Global; transcript usage	Multivariate	Uses the Dirichlet-multinomial distribution to model transcript counts of a gene in each condition and for the pooled data; To test for the differences in transcript proportions between conditions, DRIMSeq uses the LR statistic; Involves sharing information between genes, for the dispersion estimation	Allows comparison between multiple groups of samples; In sQTL analysis, a permutation approach is employed in p-value estimation to account for the dependencies between variants (SNPs)
sQTL	Lappalainen et al. (GEUVADIS) [6]	FluxCapacitor [22]	Global; transcript usage	Modeling each transcript separately	To account for the gene expression, they transformed transcript quantifications into ratios; They used a linear model implemented in Matrix eQTL to test each transcript separately for the association between its expression and genotype	FDR was estimated by permutations
sQTL	Montgomety et al. [22]	FluxCapacitor [22]	Local; splicing events	Modeling each splicing event separately	Association analyses between genotypes and splicing events conducted with Spearman rank correlation	P-value significance evaluated with permutations
sQTL	Battle et al. [23]	Cufflinks [19]	Global; transcript usage	Modeling each transcript separately	To account for the gene expression, it transforms transcript quantifications into ratios; Association analyses between genotypes and transcript ratios conducted with Spearman rank correlation	Bonferroni correction used to account for the number of SNPs tested per gene
sQTL	Pickrell et al. [24]		Local; exon usage	Modeling each exon separately	To account for the gene expression, it transforms exon quantifications into ratios; Association analyses between genotypes and splicing events were done with standard linear regressions between exon quantifications and genotypes	Using permutations for the FDR estimation

Table S2: Metadata and comparisons done for pasilla data with *DEXSeq* and *DRIMSeq*. Additionally, for *DEXSeq* which is implemented as a GLM framework, a model that compares the control and knock-down samples (like in model full) and takes into account the library layout was fitted (model full 2).

Sample Name	Condition	Library Layout	Model full	Model full paired	Model null1	Model null2	Model null3
GSM461176	CTL	SINGLE	c1	-	c1	c1	c1
GSM461177	CTL	PAIRED	c1	c1	c2	c1	c2
GSM461178	CTL	PAIRED	c1	c1	c1	c2	c2
GSM461179	KD	SINGLE	c2	-	-	-	-
GSM461180	KD	PAIRED	c2	c2	-	-	-
GSM461181	KD	PAIRED	c2	c2	-	-	-
GSM461182	CTL	SINGLE	c1	-	c2	c2	c1

Table S3: Metadata and comparisons done for adenocarcinoma data with *DEXSeq* and *DRIMSeq*. Additionally, for *DEXSeq* which is implemented as a GLM framework, a model that compares the normal and tumor samples (like in model full) and takes into account the patient ID was fitted (model full 2).

Sample Name	Condition	Patient ID	Model full	Model null normal1	Model null normal2	Model null tumor1	Model null normal2
GSM927308	normal	1	c1	c1	c1	-	-
GSM927309	tumor	1	c2	-	-	c1	c1
GSM927310	normal	3	c1	c1	c2	-	-
GSM927311	tumor	3	c2	-	-	c1	c2
GSM927312	normal	4	c1	c1	c1	-	-
GSM927313	tumor	4	c2	-	-	c1	c1
GSM927314	normal	5	c1	c2	c2	-	-
GSM927315	tumor	5	c2	-	-	c2	c2
GSM927316	normal	6	c1	c2	c1	-	-
GSM927317	tumor	6	c2	-	-	c2	c1
GSM927318	normal	8	c1	c2	c2	-	-
GSM927319	tumor	8	c2	-	-	c2	c2

Table S4: Overlap of *DRIMSeq* and *sQTLseekeR* tuQTLs detected in the CEU population from the GEUVADIS project at FDR = 0.05 with sQTLs from other analyses.

	DRIMSeq	sQTLseekeR	Detected tested	Total detected
GEUVADIS trQTLs EUR all	2,475	2,466	16,826	83,266
GEUVADIS trQTLs EUR best	15	26	266	536
GLiMMPS psiQTLs CEU	9	10	91	112
	DRIMSeq	sQTLseekeR	Validated tested	Total validated
GLiMMPS psiQTLs CEU GWAS	1	1	10	10
GLiMMPS psiQTLs CEU PCR	2	2	26	26

Table S5: Overlap of *DRIMSeq* and *sQTLseekeR* tuQTLs detected in the YRI population from the GEUVADIS project at FDR = 0.05 with sQTLs from other analyses.

	DRIMSeq	sQTLseekeR	Detected tested	Total detected
GEUVADIS trQTLs YRI all	1,047	1,226	1,882	3,563
GEUVADIS trQTLs YRI best	7	20	50	75
GLiMMPS psiQTLs CEU	4	7	78	112
	DRIMSeq	sQTLseekeR	Validated tested	Total validated
GLiMMPS psiQTLs CEU GWAS	0	2	9	10
GLiMMPS psiQTLs CEU PCR	1	3	22	26

References

[1] Mark D Robinson, Davis J McCarthy, and Gordon K Smyth. edgeR: a Bioconductor package for differential expression analysis of digital gene expression data. *Bioinformatics (Oxford, England)*, 26(1):139–140, 2010.

[2] Charlotte Soneson, Katarina L Matthes, Malgorzata Nowicka, Charity W Law, and Mark D Robinson. Isoform prefiltering improves performance of count-based methods for analysis of differential transcript usage. *Genome Biology*, 17(1):1–15, 2016.

[3] Malgorzata Nowicka. *PasillaTranscriptExpr: Data package with transcript expression obtained with kallisto from pasilla knock-down RNA-Seq data from Brooks et al.*, 2016. R package version 1.0.0.

[4] Angela N Brooks, Li Yang, Michael O Duff, Kasper D Hansen, Jung W Park, Sandrine Dudoit, Steven E Brenner, and Brenton R Graveley. Conservation of an RNA regulatory map between Drosophila and mammals. *Genome research*, 21(2):193–202, 2011.

[5] Malgorzata Nowicka. *GeuvadisTranscriptExpr: Data package with transcript expression and bi-allelic genotypes from the GEUVADIS project*, 2016. R package version 1.0.0.

[6] Tuuli Lappalainen, Michael Sammeth, Marc R Friedländer, Peter A C 't Hoen, Jean Monlong, Manuel A Rivas, Mar Gonzàlez-Porta, Natalja Kurbatova, Thasso Griebel, Pedro G Ferreira, Matthias Barann, Thomas Wieland, Liliana Greger, Maarten van Iterson, Jonas Almlöf, Paolo Ribeca, Irina Pulyakhina, Daniela Esser, Thomas Giger, Andrew Tikhonov, Marc Sultan, Gabrielle Bertier, Daniel G MacArthur, Monkol Lek, Esther Lizano, Henk P J Buermans, Ismael Padioleau, Thomas Schwarzmayr, Olof Karlberg, Halit Ongen, Helena Kilpinen, Sergi Beltran, Marta Gut, Katja Kahlem, Vyacheslav Amstislavskiy, Oliver Stegle, Matti Pirinen, Stephen B Montgomery, Peter Donnelly, Mark I McCarthy, Paul Flicek, Tim M Strom, Hans Lehrach, Stefan Schreiber, Ralf Sudbrak, Angel Carracedo, Stylianos E Antonarakis, Robert Häsler, Ann-Christine Syvänen, Gert-Jan van Ommen, Alvis Brazma, Thomas Meitinger, Philip Rosenstiel, Roderic Guigó, Ivo G Gut, Xavier Estivill, and Emmanouil T Dermitzakis. Transcriptome and genome sequencing uncovers functional variation in humans. *Nature*, 501(7468):506–11, 2013.

[7] Keyan Zhao, Zhi-Xiang Lu, Juw Won Park, Qing Zhou, and Yi Xing. GLiMMPS: Robust statistical model for regulatory variation of alternative splicing using RNA-seq data. *Genome biology*, 14(7):R74, 2013.

[8] Vivian G. Cheung, Renuka R. Nayak, Isabel Xiaorong Wang, Susannah Elwyn, Sarah M. Cousins, Michael Morley, and Richard S. Spielman. Polymorphic cis- and Trans-Regulation of human gene expression. *PLoS Biology*, 8(9), 2010.

[9] T (EBI) Burdett, PN (NHGRI) Hall, E (EBI) Hastings, LA (NHGRI) Hindorff, HA (NHGRI) Junkins, AK (NHGRI) Klemm, J (EBI) MacArthur, TA (NHGRI) Manolio, J (EBI) Morales, H (EBI) Parkinson, and D (EBI) Welter. The NHGRI-EBI Catalog of published genome-wide association studies.

[10] S. Anders, A. Reyes, and W. Huber. Detecting differential usage of exons from RNA-seq data. *Genome Research*, 22(10):2008–2017, 2012.

[11] Simon Anders, Paul Theodor Pyl, and Wolfgang Huber. HTSeq-A Python framework to work with high-throughput sequencing data. *Bioinformatics*, 31(2):166–169, 2015.

[12] Halit Ongen and Emmanouil T. Dermitzakis. Alternative Splicing QTLs in European and African Populations. *American Journal of Human Genetics*, 97(4):567–575, 2015.

[13] Halit Ongen, Alfonso Buil, Andrew Anand Brown, Emmanouil T Dermitzakis, and Olivier Delaneau. Fast and efficient QTL mapper for thousands of molecular phenotypes. *Bioinformatics (Oxford, England)*, pages 1–7, 2015.

[14] Shihao Shen, Juw Won Park, Zhi-xiang Lu, Lan Lin, Michael D Henry, Ying Nian Wu, Qing Zhou, and Yi Xing. rMATS: robust and flexible detection of differential alternative splicing from replicate RNA-Seq data. *Proceedings of the National Academy of Sciences of the United States of America*, 111(51):E5593–601, 2014.

[15] Cheng Jia, Yu Hu, Yichuan Liu, and Mingyao Li. Mapping Splicing Quantitative Trait Loci in RNA-Seq. *Cancer Informatics*, 13:35–43, 2014.

[16] Yu Hu, Yichuan Liu, Xianyun Mao, Cheng Jia, Jane F. Ferguson, Chenyi Xue, Muredach P. Reilly, Hongzhe Li, and Mingyao Li. PennSeq: Accurate isoform-specific gene expression quantification in RNA-Seq by modeling non-uniform read distribution. *Nucleic Acids Research*, 42(3), 2014.

[17] Yarden Katz, Eric T Wang, Edoardo M Airoldi, and Christopher B Burge. Analysis and design of RNA sequencing experiments for identifying isoform regulation. *Nat Methods*, 7(12):1009–1015, 2010.

[18] Cole Trapnell, David G Hendrickson, Martin Sauvageau, Loyal Goff, John L Rinn, and Lior Pachter. Differential analysis of gene regulation at transcript resolution with RNA-seq. *Nature biotechnology*, 31(1):46–53, 2013.

[19] Cole Trapnell, Brian a Williams, Geo Pertea, Ali Mortazavi, Gordon Kwan, Marijke J van Baren, Steven L Salzberg, Barbara J Wold, and Lior Pachter. Transcript assembly and quantification by RNA-Seq reveals unannotated transcripts and isoform switching during cell differentiation. *Nature biotechnology*, 28(5):511–515, 2010.

[20] Jean Monlong, Miquel Calvo, Pedro G. Ferreira, and Roderic Guigó. Identification of genetic variants associated with alternative splicing using sQTLseekeR. *Nature Communications*, 5(May):4698, aug 2014.

[21] Yang I Li, David A Knowles, and Jonathan K Pritchard. LeafCutter: Annotation-free quantification of RNA splicing. *bioRxiv*, mar 2016.

[22] Stephen B Montgomery, Micha Sammeth, Maria Gutierrez-Arcelus, Radoslaw P Lach, Catherine Ingle, James Nisbett, Roderic Guigo, and Emmanouil T Dermitzakis. Transcriptome genetics using second generation sequencing in a Caucasian population. *Nature*, 464(7289):773–777, 2010.

[23] Alexis Battle, Sara Mostafavi, Xiaowei Zhu, James B. Potash, Myrna M. Weissman, Courtney McCormick, Christian D. Haudenschild, Kenneth B. Beckman, Jianxin Shi, Rui Mei, Alexander E. Urban, Stephen B. Montgomery, Douglas F. Levinson, and Daphne Koller. Characterizing the genetic basis of transcriptome diversity through RNA-sequencing of 922 individuals. *Genome Research*, 24(1):14–24, 2014.

[24] Joseph K Pickrell, John C Marioni, Athma A Pai, Jacob F Degner, Barbara E Engelhardt, Everlyne Nkadori, Jean-Baptiste Veyrieras, Matthew Stephens, Yoav Gilad, and Jonathan K Pritchard. Understanding mechanisms underlying human gene expression variation with RNA sequencing. *Nature*, 464(7289):768–772, 2010.
