# Supplementary material for: DRIMSeq: a Dirichlet-multinomial framework for multivariate count outcomes in genomics
Source: F1000Res. 2016 Dec 6;5:1356. Originally published 2016 Jun 13. [Version 2] doi: 10.12688/f1000research.8900.2 (PMC5200948; doi:10.12688/f1000research.8900.2)
Supplement: Peer review history

# Peer review: Referee response for version 2

**DOI:** 10.5256/f1000research.11139.r18253

**Published:** 2016-12-20

**Authors:** Robert Castelo

**Affiliations:** 1 Department of Experimental and Health Sciences, Pompeu Fabra University, Barcelona, Spain

I appreciate that the authors have made an effort to address my comments and I'm particularly happy to see that my suggestion to check the overlap of tuQTLs with splice site binding sites reveals an improved enrichment by DRIMSeq. I also understand now the decision you took about not using the 'SummarizedExperiment' class. For the future development of DRIMSeq you may want to consider using the MultiAssayExperiment class ( http://bioconductor.org/packages/MultiAssayExperiment) that allows multiple assay types over multiple sample sets.

 The authors say that it is not worth made a comparison with Cufdiff because in the study by Soneson ^1^ *et al.* (2016), where both authors of DRIMSeq were involved, Cudiff was very conservative in detecting differential isoform/transcript usage (DTU). In that paper the authors assess DTU by switching the two most abundant isoforms and show that Cufdiff has a low true positive rate (TPR) at small magnitudes of the difference in relative abundance between the two most abundant isoforms per gene. However, in Supplementary Figure 10 of that paper, the authors show that at larger magnitudes of that difference, the TPR of Cufdiff improves substantially while correctly controlling the false discovery rate (FDR).

 In this paper the authors assess DTU following the same strategy of switching the two most abundant isoforms and I think it would be again very interesting to see how Cufdiff and DRIMSeq compare at different magnitudes of the change in isoform usage. The authors also argue that Frazee ^2^ *et al.* (2014) find that Cuffdiff is very conservative. However, as far as I understand that paper, Frazee and co-workers are not evaluating DTU but differential transcript expression (DTE), and therefore, in my view, the experiments conducted on that paper do not warrant the conclusion that Cufdiff is overly conservative for DTU.

 The authors decided not to perform an enrichment analysis of tuQTLs on ESEs and ESSs because Lalonde ^3^ *et al.* (2011) concluded that ESE predictions themselves are a poor indicator of the effect of SNPs on splicing patterns. However, Lalonde and co-workers scored ESE motifs with ESEfinder 3.0 (Cartegni ^4^ *et al.* 2003), a method based on SELEX experiments conducted about 10 years ago and I would expect some advance in this field in the last decade. A recent study that seems to successfully use more recent ESE and ESS data to assess their enrichment with respect to polymorphisms is Supek ^4^ *et al.* (2014).

 While these two aspects remain, in my opinion, open, I think the statistical model of DRIMSeq proposed for DTU makes a lot sense and people interested in addressing biological questions that involve DTU should give it try, and I'm happy to approve the paper.

I have read this submission. I believe that I have an appropriate level of expertise to confirm that it is of an acceptable scientific standard.

## References

[1] SonesonCMatthesKLNowickaMLawCWRobinsonMD: Isoform prefiltering improves performance of count-based methods for analysis of differential transcript usage. *Genome Biol*.2016;17: 10.1186/s13059-015-0862-3 12 10.1186/s13059-015-0862-3 26813113PMC4729156

[2] FrazeeACPerteaGJaffeAELangmeadBSalzbergSLLeekJT: Flexible isoform-level differential expression analysis with Ballgown. *bioRxiv*.2014; https://doi.org/10.1101/003665 003665 10.1101/003665

[3] LalondeEHaKCWangZBemmoAKleinmanCLKwanTPastinenTMajewskiJ: RNA sequencing reveals the role of splicing polymorphisms in regulating human gene expression. *Genome Res*.2011;21(4) : 10.1101/gr.111211.110 545-54 10.1101/gr.111211.110 21173033PMC3065702

[4] SupekFMiñanaBValcárcelJGabaldónTLehnerB: Synonymous mutations frequently act as driver mutations in human cancers. *Cell*.2014;156(6) : 10.1016/j.cell.2014.01.051 1324-35 10.1016/j.cell.2014.01.051 24630730

# Peer review: Referee response for version 1

**DOI:** 10.5256/f1000research.9577.r14580

**Published:** 2016-07-06

**Authors:** Robert Castelo

**Affiliations:** 1 Department of Experimental and Health Sciences, Pompeu Fabra University, Barcelona, Spain

This article introduces a new statistical method, called DRIMSeq and implemented in a R/Bioconductor package of the same name, to detect isoform expression changes between two conditions from RNA-seq data. The same method can be used to search for significant associations between SNPs and isoform quantifications obtained also from RNA-seq data (sQTLs).  The main novelty of this method with respect to the existing literature on this problem, is the joint modelling of transcript quantification values derived from isoforms of the same gene, by using a Dirichlet-multinomial model. This allows the method to account of the intrinsic dependency between quantification values of these isoforms.

 The assessment of DRIMSeq on differential isoform usage provides a comparison of its performance with DEXSeq ^1^, a statistical method for differential exon inclusion from RNA-seq data, as function of two different "isoform" quantification strategies: exonic-bin (not really "isoform") count values calculated with HTSeq and transcript-quantification values calculated with kallisto ^2^.

 The experimental results make perfect sense, DRIMSeq works better than DEXSeq with transcript-quantification values and DEXSeq works better than DRIMSeq with exonic-bin count values. However, while both methods, and both types of "isoform" quantification input data, allow one to study the post-transcriptional processing of RNA transcripts, the kind of questions that can be addressed with each of them are different. Exonic-bin count values and DEXSeq can be used to investigate differential exon inclusion across conditions, which is a consequence of differential isoform usage, while transcript-quantification values and DRIMSeq can be used to directly investigate differential isoform usage.

 A potentially interesting outcome of this comparison in the paper could be some sort of guidelines about when is it more sensible to investigate differential exon inclusion or differential isoform usage, depending on factors such as the biological question at hand, sequencing depth or number of biological replicates. However, this is apparently beyond the scope of this paper and the experimental results are in principle geared towards convincing the reader that DRIMSeq improves on existing approaches to discover changes in isoform usage, as suggested in the abstract. In my view, the experimental results do not address this question and I would suggest the authors to compare DRIMSeq with methods that also work with transcript-quantification values and assess differential isoform usage such as, for instance, Cuffdiff ^3^ or sleuth ^4^.

 The experimental results on searching for sQTLs compare favourably DRIMSeq with an existing tool for that purpose, sQTLseekR ^5^. Evaluating performance in this context is challenging and the idea of assessing enrichment with respect to splicing-related features is a good one. However, the (two) presented features in Table 1 could be made more precise. It is unclear that a SNP close to a GWAS hit should be necessarily related to splicing and it is also unclear why one should expect splicing-related enrichment more than a few hundred nucleotides away from the intervening exon. While it is technically interesting to see a method being used to address two completely different research questions, in my view, mixing both types of analyses makes the article less focused. I would argue that both questions deserve separate papers, and that would allow the authors to investigate in depth critical aspects of both types of analysis that are currently not addressed in the current article.

 In summary, this article provides an interesting new methodology for the analysis of differential isoform usage from RNA-seq data, it is well-written and the implemented software runs smoothly and is well documented. However, in my view, the current experimental results of the article are not that informative for the reader to learn what advantages DRIMSeq provides over other tools for differential isoform usage analysis, and to decide whether he/she should be doing a differential isoform usage, or a differential exon inclusion analysis, if this were a goal of the comparison with DEXSeq.

 Minor comments:

1. I would replace the term "edgeR ideology" in page 5 by "edgeR strategy".
2. In page 9 it is described that the distributions of raw p-values shown in Supplementary Figures S28 and S29 fit "better" when derived from transcript quantification values than from exonic-bin count values, but in fact in both cases the distributions are non-uniform for p-values distributed under the null hypothesis. This can be easily shown with the data from vignette of the DRIMSeq package when skipping the step that reduces the transcript set to analyze to speed up the building time of the vignette. This is not openly discussed in the paper but I would argue that it is quite critical to know under what technical assumptions the proposed hypothesis test leads to uniform raw p-values under the null, as this has a direct consequence on the control of the probability of the type-I error.
3. The sQTL analysis described in pages 9, 10 and 11 uses transcript-quantification values from FluxCapacitor. If the entire first part of the paper shows the performance metrics of DRIMSeq using kallisto, in my view, it would make more sense to use kallisto for this analysis as well.
4. With regard to the implementation in the R/Bioconductor software package DRIMSeq, the authors have implemented a specialized S4 object class called 'dmDSdata' to act as a container for counts and information about samples.  Since the package forms part of the Bioconductor project, I think it would better for both, the end-user and the developer authors, that the package re-uses the 'SummarizedExperiment' class as container for counts and sample information. This would facilitate the integration of DRIMSeq into existing or new workflows for the analysis of RNA-seq data. As an example of the limitations derived from providing a completely new specialized object class, the dimensions of a 'dmDSdata' object in terms of number of features and number of samples cannot be figured out using the expected call to the 'dim()' accessor method. Of course the authors may add that method to the 'dmDSdata' object class but, in general, there are obvious advantages derived from enabling data interoperability through the use of common data structures across Bioconductor software packages ^6^.

I have read this submission. I believe that I have an appropriate level of expertise to confirm that it is of an acceptable scientific standard, however I have significant reservations, as outlined above.

## References

[1] AndersSReyesAHuberW: Detecting differential usage of exons from RNA-seq data. *Genome Res*.2012;22(10) : 10.1101/gr.133744.111 2008-17 10.1101/gr.133744.111 22722343PMC3460195

[2] BrayNLPimentelHMelstedPPachterL: Near-optimal probabilistic RNA-seq quantification. *Nat Biotechnol*.2016;34(5) : 10.1038/nbt.3519 525-7 10.1038/nbt.3519 27043002

[3] TrapnellCHendricksonDGSauvageauMGoffLRinnJLPachterL: Differential analysis of gene regulation at transcript resolution with RNA-seq. *Nat Biotechnol*.2013;31(1) : 10.1038/nbt.2450 46-53 10.1038/nbt.2450 23222703PMC3869392

[4] PimentelH: Differential analysis of RNA-Seq incorporating quantification uncertainty. *bioRxiv*.2016;058164: 10.1101/058164 10.1101/058164 Reference source 28581496

[5] MonlongJCalvoMFerreiraPGGuigóR: Identification of genetic variants associated with alternative splicing using sQTLseekeR. *Nat Commun*.2014;5: 10.1038/ncomms5698 4698 10.1038/ncomms5698 25140736PMC4143934

[6] HuberWCareyVJGentlemanRAndersSCarlsonMCarvalhoBSBravoHCDavisSGattoLGirkeTGottardoRHahneFHansenKDIrizarryRALawrenceMLoveMIMacDonaldJObenchainVOleśAKPagèsHReyesAShannonPSmythGKTenenbaumDWaldronLMorganM: Orchestrating high-throughput genomic analysis with Bioconductor. *Nat Methods*.2015;12(2) : 10.1038/nmeth.3252 115-21 10.1038/nmeth.3252 25633503PMC4509590

# Response

**Published:** 2016-11-25

**Authors:** Mark Robinson

**Affiliations:** University of Zurich, Switzerland

Thank you for taking the time to read and review our paper.

*DEXSeq* is a package designed for the differential exon usage (DEU) and returns exon-level p-values, which can be also summarized to the gene level. In principle, *DEXSeq*’s implementation could be used to address the question of differential isoform/transcript usage (DTU) as well, which was done, for example, in the simulation study by Soneson *et al.* [1]. They use different counting strategies, among them transcript quantifications from *kallisto* [2], coupled with *DEXSeq*’s differential engine to detect differential transcript usage. *DRIMSeq*, based on the Dirichlet-multinomial model, was developed to detect differential usage of any kind of multivariate genomic features at the gene-level. Thus potentially, both *DEXSeq* and *DRIMSeq* can be applied to exon counts and to transcript quantifications. However, from our comparisons, which were performed at the gene-level, the performance of *DEXSeq* and *DRIMSeq* is different on these different types of counts. *DEXSeq* performs better on exon counts and *DRIMSeq* on transcript counts.

 We have not used *Cuffdiff* [3] in our comparisons here because in the study by Soneson *et al.* [1], it performed poorly compared to *DEXSeq*. In particular, *Cuffdiff* was very conservative having low false discovery rate (FDR) at the cost of very low power for detecting DTU. The conservative nature of *Cuffdiff* for differential transcript expression, was also pointed out by Frazee *et al.* [4]. We decided to compare *DRIMSeq* only to the top performing method, *DEXSeq*. The other tool proposed by the Reviewer, sleuth [5], is meant for differential transcript expression analyses, not DTU.

 The scope of this paper was not to justify exon or transcript level analysis, for that one could refer to the comparison paper by Hooper [6], but to propose a methodologically-sound tool for differential isoform usage analysis or detect transcript usage QTLs based on transcript quantifications. We propose to use *DRIMSeq* since it outperformed *DEXSeq* in this type of analysis and there are no other tools for differential transcript usage that were intended for transcript level quantifications from the latest generation of fast quantification tools, such as *kallisto* [2] or *Salmon* [7].

 Importantly, *DEXSeq* returns p-values per feature (exon or transcript), which can be also summarized to the gene level. *DRIMSeq* performs gene-level tests and returns p-values per gene only. When the interest is in detecting specific exons or isoforms that change, one should use *DEXSeq* because currently *DRIMSeq* does not provide any post hoc analysis (although in many cases, the relevant information can be deduced from looking at the relative transcript expression from *DRIMSeq*’s plots). We have not investigated the differences in performance due to sequencing depth or number of biological replicates, but we believe that the requirements would be basically the same in these terms for both of the methods. What matters is the completeness of annotation. Detecting DTU based on exon counts is generally more robust than that based on transcript quantifications when the annotation is incomplete, which was investigated in detail by Soneson *et al.* [1].

 To compare the performance of *DRIMSeq* and *sQTLseekeR*, we use the splicing-related features that were also used in the *sQTLseekeR* paper [8] to compare *sQTLseekeR* against other methods. The Reviewer suggested to consider other splicing-related features, such as exonic splicing enhancers (ESEs), exonic splicing silencers (ESSs) and splice sites. We have added the frequency of tuQTL overlapping with the splice sites to Table 1. However, we have not performed analyses on ESEs and ESSs since Lalonde *et al.* [9] concluded from their study that "ESE predictions themselves are a poor indicator of the effect of SNPs on splicing patterns".

 By addressing differential splicing and sQTLs in one paper, our aim was to show that methods used for these analyses are based on statistical approaches that in the end tackle ultimately the same question: differential splicing between conditions. Both analyses employ the same methods for gene feature quantification and potentially one main differential engine could be used with slight analysis- specific adjustments, such as information sharing between genes for small sample size data or using genotypes as grouping factor, which is done in *DRIMSeq*. We believe we have addressed in sufficient depth aspects of both of these analyses providing comparisons on simulated and real data.

 Addressing the minor comments:

- We have replaced the term "edgeR ideology" in page 5 by "edgeR strategy".
- As suggested, we have investigated in more depth, based on simulations from the DM model, the *DRIMSeq *p-value distributions under the null hypothesis of no differential transcript usage (Figures 1, S4, S6, S11, S14). Overall, using the Cox-Reid adjusted profile likelihood and the dispersion moderation leads to p-value distributions that in most cases are closer to the uniform distribution (Figures 1D, S4 and S11). The better fit of the DM model to transcript counts in comparison to exon counts can be seen in Figure S14, where the p-value distributions are more uniform for simulations that mimic *kallisto* counts than for simulations that mimic *HTseq* counts.
- Yes, using *kallisto* counts would be more consistent with the rest of our manuscript. Nevertheless, we decided to to use the Flux Capacitor counts because they were already available on the GEUVADIS project website and have been used extensively in other projects, for example, in the sQTLseekeR paper. Moreover, we think that using other counts should not affect the comparison between DRIMSeq and sQTLseekeR.
- We had already considered the SummarizedExperiment class while developing the DRIMSeq package. However, it does not provide features and functionality that we need for storing the count data and DRIMSeq results. In particular, the dimensions of Assays in SummarizedExperiment must be the same. That is not the case for us for two reasons. Firstly, each gene has multiple transcripts and, for example, the table with proportion estimates per transcript is larger than a table with dispersion estimates which are available per gene. Second, in the QTL analysis, table with transcript counts has different dimensions than table with genotypes. Additionally, we use matrices instead of data frames to store our data because the former occupies less space. Specifically, we have created a class called MatrixList, which is adjusted to store data where each gene has multiple features quantified and allows a quick access to these counts in per gene basis. We have not implemented the dim() method on dmDSdata or dmSQTLdata because we want to keep consistency between them and, for example, dmSQTLdata contains transcript counts and genotypes which have different dimensions. Thus we decided to make the dim() methods available for the counts and genotypes slots in these classes but not for the classes themselves.

References

 [1]  Charlotte Soneson, Katarina L Matthes, Malgorzata Nowicka, Charity W Law, and Mark D Robinson. Isoform prefiltering improves performance of count-based methods for analysis of differential transcript usage. Genome Biology, 17(1):1–15, 2016.

 [2]  Nicolas L Bray, Harold Pimentel, Pall Melsted, and Lior Pachter. Near-optimal probabilistic RNA-seq quantification. Nat Biotech, advance on, Apr 2016.

 [3]  Cole Trapnell, David G Hendrickson, Martin Sauvageau, Loyal Goff, John L Rinn, and Lior Pachter. Differential analysis of gene regulation at transcript resolution with RNA-seq. Nature biotechnology, 31(1):46–53, 2013.

 [4]  A. C. Frazee, G. Pertea, a. E. Jaffe, B. Langmead, S. L. Salzberg, and J. T. Leek. Flexible isoform-level differential expression analysis with Ballgown. bioRxiv, pages 0–13, 2014.

 [5]  Harold J Pimentel, Nicolas Bray, Suzette Puente, Páll Melsted, and Lior Pachter. Differential analysis of RNA-Seq incorporating quantification uncertainty. bioRxiv, Jun 2016.

 [6]  Joan E Hooper. A survey of software for genome-wide discovery of differential splicing in RNA-Seq data. Human genomics, 8:3, 2014.

 [7]  Rob Patro, Geet Duggal, and Carl Kingsford. Salmon: Accurate, Versatile and Ultrafast Quantification from RNA-seq Data using Lightweight-Alignment. bioRxiv, page 021592, 2015.

 [8]  Jean Monlong, Miquel Calvo, Pedro G. Ferreira, and Roderic Guigó. Identification of genetic variants associated with alternative splicing using sQTLseekeR. Nature Communications, 5(May):4698, Aug 2014.

 [9]  Emilie Lalonde, Kevin C H Ha, Zibo Wang, Amandine Bemmo, Claudia L Kleinman, Tony Kwan, Tomi Pastinen, and Jacek Majewski. RNA sequencing reveals the role of splicing polymorphisms in regulating human gene expression. Genome Research, 21(4):545–554, Apr 2011.

# Peer review: Referee response for version 1

**DOI:** 10.5256/f1000research.9577.r14338

**Published:** 2016-06-24

**Authors:** Alejandro Reyes

**Affiliations:** 1 Genome Biology Unit, European Molecular Biology Laboratory, Heidelberg, Germany

Nowicka and Robinson propose a novel method, called DRIMSeq, to test for differential transcript usage between groups of samples using RNA-seq. The method is based on the Dirichlet-multinomial distribution.

 The authors evaluate different existing approaches to estimate the parameters of their model using simulated experiments with a small number of replicates, which is a common scenario of high-throughput sequencing experiments. Furthermore, Nowicka *et al*. provide a proof of principle of their method by applying it to both simulated and real RNA-seq data. They also compare the performance of DRIMSeq with DEXSeq and sQTLseekeR in detecting differential transcript usage and splicing quantitative trait loci (sQTLs), respectively. DRIMSeq shows high concordance with DEXSeq. Furthermore, the authors demonstrate that DRIMSeq performs better than DEXSeq when using transcript-level counts. DRIMSeq and sQTLseekeR were also highly concordant. Nevertheless, sQTL genes detected by DRIMSeq were expressed higher than those detected by sQTLseekeR, and sQTLs detected by DRIMSeq were in closer proximity to exons compared to sQTLs detected by sQTLseeker. DRIMSeq is implemented as an R/Bioconductor package.

 Overall, the manuscript is well presented and is scientifically sound. The description of the method is clear, the comparisons are fair, and the conclusions are supported by data and analyses.

 Below some minor comments:

  

1. Transcription of multiple isoforms from a single gene can be the consequence of differences in the following molecular mechanisms: transcription start sites, splicing, and termination of transcription.  The terms “differential splicing” and “splicing QTLs”, which are used throughout the manuscript and the package vignette, focus only on splicing. Consider a hypothetical example of an isoform switch between conditions in which the two isoforms only diverge by the transcription start site of the first exon. DRIMSeq should also detect this difference, and this would not be due to differential splicing. Thus, the authors could use more generic terminology that describes all possible interpretations of the outcome of their test. Perhaps “differential transcript usage” or “transcript usage QTLs”?
2. In equations 6-11, *PL* and *APL* are understandable from the context but are not defined in the text. 
3. It would be useful for the reader to include more information of the simulated data from Sonseson *et al*. (2016) in the main text of this manuscript (for example, number of replicates per condition).
4. The authors describe how DEXSeq can account for additional covariates in complex experimental designs. This paragraph, as well as the figures and supplementary material associated to it, could be understood as if DEXSeq fits GLMs only for complex experimental designs. In reality, DEXSeq always fits GLMs, even for simple two-group comparisons.
5. There are some panels from the supplementary figures where data are missing. Specifically, Fig. S13 has 3 empty panels and Fig. S21 the left panels are missing the data for “dexseq.prefilter5” and “drimseq_genewise_grid_trended.filter5”.
6. The list of software for splicing event quantification is already very extensive, however a citation to the Bioconductor package SGSeq (Goldstein *et al.*, 2016) could also be added.
7. As for the readability of the supplementary information, some abbreviations are not defined in each supplementary figure caption. For example, in Fig. S5, n, m, DM, FP and nr_features are not defined in its caption (some of them, however, are defined in previous captions).  Since many abbreviations repeat several times through the supplementary information, it would be useful to include a glossary of all abbreviations at the beginning of all supplementary figures. 

I have read this submission. I believe that I have an appropriate level of expertise to confirm that it is of an acceptable scientific standard.

## References

[1] SonesonCMatthesKLNowickaMLawCWRobinsonMD: Isoform prefiltering improves performance of count-based methods for analysis of differential transcript usage. *Genome Biol*.2016;17: 10.1186/s13059-015-0862-3 12 10.1186/s13059-015-0862-3 26813113PMC4729156

[2] GoldsteinLDCaoYPauGLawrenceMWuTDSeshagiriSGentlemanR: Prediction and Quantification of Splice Events from RNA-Seq Data. *PLoS One*.2016;11(5) : 10.1371/journal.pone.0156132 e0156132 10.1371/journal.pone.0156132 27218464PMC4878813

# Response

**Published:** 2016-11-25

**Authors:** Mark Robinson

**Affiliations:** University of Zurich, Switzerland

Thank you for taking the time to read and review our paper.

 As per your suggestion, we have now stressed that DRIMSeq can be applied to differential transcript usage (DTU), which accounts for not only differential splicing but also the differences in transcription start sites and differential transcript termination. In the QTL analysis, as we test for associations between genotypes and transcript usage and not only splicing, following your suggestion, we have also changed the term from splicing QTLs (sQTLs) to transcript usage QTLs (tuQTLs).

 We have addressed all the other minor comments which include:

- defining the abbreviations of profile likelihood (PL) and adjusted profile likelihood (APL),
- adding the sample size information about the simulations from Soneson *et al.* [1],
- in order to remove the misleading suggestion that DEXSeq fits GLMs only in the complex designs, we have changed the names of the models used in real data analysis from "model full glm" to "model full 2" and paraphrased the corresponding manuscript sections,
- we have included results for the panels with missing data in the Supplementary Figures S15, S16 and S24,
- we have included the citation to SGSeq [2] - the Bioconductor package for analyzing splice events from RNA-seq data,
- in the Supplementary Materials, we have prepared a section explaining abbreviations used in the subsequent Supplementary Figures.

References

 [1]  Charlotte Soneson, Katarina L Matthes, Malgorzata Nowicka, Charity W Law, and Mark D Robinson. Isoform prefiltering improves performance of count-based methods for analysis of differential transcript usage. Genome Biology, 17(1):1–15, 2016.

 [2]  Leonard D Goldstein, Yi Cao, Gregoire Pau, Michael Lawrence, Thomas D Wu, Somasekar Seshagiri, and Robert Gentleman. Prediction and Quantification of Splice Events from RNA-Seq Data. PLoS ONE, 11(5):e0156132, may 2016.
